# 17β-Estradiol Suppresses Gastric Inflammatory and Apoptotic Stress Responses and Restores nNOS-Mediated Gastric Emptying in Streptozotocin (STZ)-Induced Diabetic Female Mice

**DOI:** 10.3390/antiox12030758

**Published:** 2023-03-20

**Authors:** Jeremy Sprouse, Chethan Sampath, Pandu Gangula

**Affiliations:** 1 Department of Oral Diagnostic Sciences and Research, School of Dentistry, Meharry Medical College, Nashville, TN 37208, USA; 2 Department of Endodontics, School of Dentistry, Meharry Medical College, Nashville, TN 37208, USA

**Keywords:** nitric oxide, estradiol, estrogen receptors, oxidative stress, Nrf2, NFκB, gastroparesis, nitrergic relaxation, MAPK, inflammation, apoptosis

## Abstract

Gastroparesis (Gp) is a severe complication of diabetes mellitus (DM) observed predominantly in women. It is characterized by abnormal gastric emptying (GE) without mechanical obstruction in the stomach. Nitric oxide (NO) is an inhibitory neurotransmitter produced by neuronal nitric oxide synthase (nNOS). It plays a critical role in gastrointestinal (GI) motility and stomach emptying. Here, we wanted to demonstrate the protective effects of supplemental 17β-estradiol (E_2_) on NO-mediated gastric function. We showed E_2_ supplementation to alleviate oxidative and inflammatory stress in streptozotocin (STZ)-induced diabetic female mice. Our findings suggest that daily administration of E_2_ at therapeutic doses is beneficial for metabolic homeostasis. This restoration occurs via regulating and modulating the expression/function of glycogen synthase kinase-3β (GSK-3β), nuclear factor-erythroid 2 p45-related factor 2 (Nrf2), Phase II enzymes, MAPK- and nuclear factor kappa-light-chain-enhancer of activated B cells (NFkB)-mediated inflammatory cytokines (IL-1β, IL-6, TNFα, IGF-1), and gastric apoptotic regulators. We also showed E_2_ supplementation to elevate GCH-1 protein levels in female diabetic mice. Since GCH-1 facilitates the production of tetrahydrobiopterin (BH_4_, cofactor for nNOS), an increase in GCH-1 protein levels in diabetic mice may improve their GE and nitrergic function. Our findings provide new insights into the impact of estrogen on gastric oxidative stress and intracellular inflammatory cascades in the context of Gp.

## 1. Introduction

Diabetic gastroparesis (DGp) is a gut motility disorder associated with abnormal gastric emptying (GE) when there is no mechanical obstruction along the gastrointestinal (GI) tract [1,2]. DGp is characterized by severe nausea, vomiting, bloating, early satiety, abdominal pains, nutritional deficiencies, and poor glycemic control [3]. DGp is a multifactorial condition caused by malfunction in the enteric nervous system (ENS) as well as the excitatory and inhibitory neurons [4,5]. Findings from recent studies suggest that patients with DGp lack functional coordination between these cell types [2,3,6]. Nitric oxide (NO) is an inhibitory non-adrenergic, non-cholinergic (NANC) neurotransmitter in the gut produced by neuronal nitric oxide synthase (nNOS) and secreted by inhibitory enteric neurons. It plays an important role in controlling gastrointestinal motility and transit time [4]. Therefore, DGp is associated with the dysregulation of gut–brain interaction.

Sex-related differences in the presentation of gastric motility disorders are well documented in both human and animal studies. Women and female rodents tend to experience more severe symptoms of Gp compared to their male counterparts due to naturally slower GE rates in females [7,8,9,10]. Young, healthy women are also more prone to experience Gp than age-matched men [7,8,9,10,11]. Various studies have demonstrated a positive correlation between Gp symptoms and menstruation, use of hormonal contraceptives, and use of hormone replacement in postmenopausal women [12,13]. Recent data from animal studies suggest that diabetes is more deleterious to the female ENS plexus and thus GI dysmotility [9,14,15]. Furthermore, our laboratory has demonstrated the role of ovarian hormone receptors and antioxidant activators in gastric motility in rodent models of type 2 diabetes mellitus (DM) [16,17,18,19]. These studies were key in identifying several roles that estrogen receptors and nuclear factor-erythroid 2 p45-related factor 2 (Nrf2) activators play in overcoming the insults of hyperglycemia, obesity, and inflammation on the oxidative stress response and gastric homeostasis. Particularly, we examined the systemic and gastrointestinal effects of a diet-induced hyperglycemia model in causing delayed and accelerated GE in the presence and absence of ovaries, respectively [16,17]. Through these models, we demonstrated gastric estrogen receptor influence in ovary-intact and ovariectomized rodent models that were exhibiting elevated circulatory estrogen. This study provides novel insight in evaluating the physiologic and therapeutic effects of estrogens on restoring gastric fitness in an STZ-induced diabetic mouse model mimicking type 1 DM. Here, we provide encouraging evidence of estrogenic effects on (1) Nrf2-mediated antioxidant protection and (2) gastric apoptotic markers, while further exploring (3) nuclear factor kappa B subunit 1 (NFκB) and 10 gut inflammatory cytokines in the context of alleviating the pathogenesis of gastric motility. Several studies have also identified elevated levels of estrogen and NO as the primary contributors to the observed slower GE in both pregnant and non-pregnant healthy rodents [8,20,21]. Although therapeutic options for Gp exist, many currently available drugs cause debilitating side effects and may not improve overall quality of life [6,22]. The influence of estrogens on the NO-mediated component of the gastric neuromuscular plexus is poorly understood. Understanding the role of estrogen in this aspect may be the key to explaining the sexual dimorphism observed in Gp and GE rates. This study further elaborates on the understanding of how supplemental estrogen, at physiologic and therapeutic levels, can also influence a disrupted nitrergic component of the gastric motility apparatus in an STZ-induced diabetic mouse model.

Animal studies have provided tremendous insight into the role of sex hormones in GE. Estrogens are known to mediate genomic and non-genomic biological actions through nuclear, cytoplasmic, and membrane-bound receptors [21,23]. Two estrogen receptors (ERs) are classically defined as ligand-activated transcription factors: Erα (alpha) and Erβ (beta). ERα and ERβ are expressed from two different genes and have various actions in different tissue systems of the gut. While ERs exist in the gastric and small intestinal mucosa, their presence is poorly documented in the gastrointestinal smooth muscle or ENS [24]. We have recently shown that ER activation restores glucose homeostasis, NO-mediated nitrergic relaxation, GE, and antioxidant and inflammatory responses to appropriate levels in high-fat diet-induced diabetic female mice [16].

Physiological estrogen levels fluctuate widely in humans and female rodents. Normal circulatory estrogen levels can range from very low in the diestrus stage to very high in the proestrus and estrus stages of the mouse reproductive cycle [25]. Estradiol (E_2_) has a myriad of effects on different organ systems when supplemented at different doses. It has been shown to exhibit dose-dependent responses at the cellular, tissue, organ, and whole-body levels [23,26,27,28]. In this study, we sought to demonstrate the benefits of supplemental E_2_ on NO-mediated gastric function, oxidative stress response machinery, and gastric apoptosis in diabetic female mice. We chose four different supplemental E_2_ doses that have been previously shown to mimic physiological, supraphysiological, and therapeutic concentrations in mice. The purpose of this study was to uncover the effects of E_2_ on diabetic Gp, NO-mediated gastric function, and the oxidative stress and inflammatory responses in female mice with STZ-induced diabetes.

## 2. Materials and Methods

### 2.1. Animals

All experiments were approved by the Institutional Animal Care and Use Committee (e-protocol no. 17-09-764 dtd 03/05/2018) at Meharry Medical College (MMC). Adult female C57BL/6J mice aged 8–9 weeks were purchased from The Jackson Laboratory (Bar Harbor, ME, USA). All animals were housed in a vivarium under standard conditions and allowed access to food and water ad libitum.

### 2.2. Experimental Design and STZ-Induced Diabetes

All mice were randomized and divided into two groups: control mice and mice treated with streptozotocin (STZ) to induce DM [26]. After calculating crude power analysis for study significance, we employed a commonly used methodology to induce diabetes with STZ [29,30]. The control mice were injected with sodium citrate vehicle buffer (pH 4.5). Mice in the STZ group were injected intraperitoneally with 0.1 mmol/L STZ in sodium citrate buffer, pH 4.5 (S0130, Sigma Chemical Co., St. Louis, MO, USA) at 50 mg/kg once a day for five consecutive days to induce persistent hyperglycemia, as described previously [31]. Body weight and circulatory glucose levels were assessed weekly to confirm diabetes induction via tail vein blood and a standard glucometer. After diabetes had been established, the diabetic mice were randomly assigned to four E_2_ treatment groups: 0.001 mg/kg *b.w*. (*n* = 6), 0.005 mg/kg *b.w*. (*n* = 6), 0.25 mg/kg *b.w*. (*n* = 6), or 1.0 mg/kg *b.w*. (*n* = 6). These doses have been shown to mimic physiological (0.001 mg/kg *b.w*.), peak physiological (0.005 mg/kg *b.w*.), and therapeutic (0.25 mg/kg *b.w*. and or 1.0 mg/kg *b.w*.) doses of E_2_ [16,28,32,33]. Dosing injections were administered intraperitoneally once a day at the same time each day for six weeks. GE and organ bath studies were conducted the day after the final dosing regimen.

### 2.3. Measurement of Gastric Emptying

Solid GE studies were executed as previously described [16,17,34]. Briefly, each group of mice was fasted overnight and then fed the next morning with a known amount of food with water for 3 h. At the termination of the feeding period, the mice were placed in a fresh cage and fasted for 2 hr. The leftover food was weighed to calculate the amount of food consumed. After the 2 h fasting zone, the mice were sacrificed, and stomach weights were recorded prior to emptying the stomach contents and again after. The rate of GE was calculated as follows: GE (% in 2 h) = (1 − gastric content/food intake) × 100. Gastric antrum tissues were harvested, snap frozen, and stored at −80 °C.

### 2.4. Neuromuscular Recording with Electric Field Stimulation

NO-mediated gastric relaxation was measured as reported previously [35]. Circular muscle sections of the gastric antrum were suspended between L-shaped tissue hooks in 5 mL organ baths containing Krebs buffer (pH 7.4) at 37 °C and continuously bubbled with 95% O_2_, 5% CO_2_ (DMT-USA, Inc., Ann Arbor, MI, USA). Tension of the neuromuscular strip was monitored with an isometric force transducer and analyzed with a digital recording software. A total of 2 g of passive tension was applied to the strip over an hour-long equilibration period through incremental increases of 0.5 g at 15 min intervals. The gastric neuromuscular strips were then treated with atropine, phentolamine, and propranolol (10 µM each) for 30 min to inhibit adrenergic and cholinergic transmission. Following exposure and contraction with 5-HT (100 µM), the muscle strips were stimulated with EFS (1 ms pulses for 1 min at 2 Hz) to elicit NANC relaxation. The changes in muscle activity were measured. For confirmation, this response was mediated by NO, the relaxation was measured after incubation with NO inhibitor, Nω-Nitro-L-arginine methyl ester (L-NAME, 30 min; 100 µM, N5751, Sigma, St. Louis, MO, USA). Analysis of area under the curve (AUC) of EFS-induced relaxation (AUCR) for 1 min and the baseline for 1 min (AUCB) was used to compare groups using the equation: (AUCR-AUCB)/weight of the tissue (mg) = AUC/mg tissue.

### 2.5. Evaluation of 17β-Estradiol, Insulin, MDA, IL-6, TNFα, IGF-1, and Total Nitrite Concentrations in Mouse Serum

Blood was collected from euthanized mice via cardiac puncture. The serum was isolated and stored at −80 °C until use. ELISA kits for 17β-estradiol (K3830, BioVision, Inc., Milpitas, CA, USA), insulin (90080, Crystal Chem, Elk Grove Village, IL, USA), malondialdehyde (MDA), and serum cytokines (tumor necrosis factor (TNF)-α, interleukin (IL)-6, and insulin-like growth factor 1 (IGF-1)) (EA-1091, Signosis, Santa Clara, CA, USA) were used to measure the serum levels of these compounds. Systemic total serum nitrite was assessed using a standard colorimetric assay per manufacturer’s recommended methodology (K262, BioVision, Inc., Milpitas, CA, USA).

### 2.6. Quantitative Real-Time Polymerase Chain Reaction (qRT-PCR) Analysis

Gastric antral neuromuscular tissues were harvested from mice and frozen in liquid nitrogen. Total RNA was extracted using TRIzol (Thermo Fisher Scientific, Waltham, MA, USA) per manufacturer’s protocol. The quality of RNA was determined by NanoDrop (Thermo Fisher Scientific), and the quantity was estimated by an Agilent 2100 bioanalyzer (Agilent Technologies, Houston, TX, USA). To eliminate any contaminating DNA, RNA was treated with RNase-free DNase (Invitrogen). One microgram of DNase-treated RNA was used for cDNA synthesis. The iScript cDNA synthesis kit (Bio-Rad, Hercules, CA, USA) was used to synthesize cDNA. One microliter of cDNA was used for each reaction together with the corresponding target primers. Primer sequences for target genes are listed in Table 1. Quantitative RT-PCR (qRT-PCR) was performed using the SYBR Green (Bio-Rad, Hercules, CA, USA) method. Cycling conditions were 95 °C for 3 min followed by 45 cycles of 95 °C for 30 s and 55 °C for 1 min. mRNA levels for target genes were normalized to mRNA levels for the β-actin gene, and threshold cycle (CT) numbers were calculated (i.e., 2^−ΔΔ^CT, the Ct method) according to manufacturer’s instructions. All studies were performed in the MMC Molecular Core Laboratory.

### 2.7. Subcellular Fractionation

The tissue lysates were suspended in fractionation buffer (10 mM HEPES (pH 7.9), 10 mM KC1, 1.5 mM MgCl_2_, 0.1% NP-40, 0.5 mM NaF, 200 mM Na_3_V0_4_, and 1× protease inhibitor cocktail). The cells were incubated on ice for 15 min with shaking. Lysates were centrifuged at 2600× *g* at 4 °C, and supernatants representing cytosolic fraction were collected. The precipitates were then resuspended with the modified RIPA buffer containing 1× protease inhibitor cocktail and incubated on ice for 20 min with periodic vortexing. The lysates were then cleared by centrifugation at 10,000× *g* at 4 °C, and supernatants were used as the nuclear fractions.

### 2.8. Gel Electrophoresis and Western Blot Analysis

Gastric antrum samples were homogenized, and total protein level was estimated for each lysate via bicinchoninic acid (BCA) assay. Equal amounts of protein (30 µg) from each sample were separated on 6% and 12% SDS polyacrylamide gels. The gel was then transferred to nitrocellulose membranes in cold environment. Each membrane was incubated in 5% dried non-fat milk in TBST for 1 h and then incubated overnight with respective primary polyclonal antibodies including: ERα, (sc-8005,1:500), ERβ (sc-390243, 1:500), GCH-1 (sc-271482, 1:500), GCLC (sc-390811, 1:1000), Nrf2 (sc-365949, 1:1000), GCLM (sc-55586, 1:1000), MAPK (sc-81621, 1:1000), NFκB (sc-8414, 1:1000), and NQO-1 (sc-376023, 1:1000) (each from Santa Cruz Biotechnology, Santa Cruz, CA, USA). nNOSα (N-terminal, ab76067, 1:1000) was purchased from Abcam (Cambridge, MA, USA). Following incubation, the membranes were washed three times for 5 min each time in 0.1% TBS-Tween and then exposed to horseradish peroxidase-conjugated secondary antibody (Santa Cruz Biotechnology, Santa Cruz, CA, USA) (1:1000) for 1 hr at room temperature. The membranes were visualized using an ECL Western blotting detection reagent (GE Healthcare Bio-Sciences Corp., Piscataway, NJ, USA), and the optimal reactive bands were analyzed using ImageQuant 500 (GE Healthcare Bio-Sciences Corp., Piscataway, NJ, USA). Optical densitometry was measured using Image Lab software (BioRad, Hercules, CA, USA). Blots were stripped and blocked overnight in 5% milk in TBST. Stripped blots were re-probed with β-actin polyclonal antibody (Sigma Chemical, St. Louis, MO, USA) for 30 min to enable normalization of luminescence signals between samples.

### 2.9. Statistical Analysis

All data are presented as mean ± standard error (SE). Statistical significance among groups was measured using Student’s *t*-test for homogeneity and the Tukey test after one-way analysis of variance (ANOVA). A *p* value of less than 0.05 was considered statistically significant.

## 3. Results

### 3.1. E_2_ Supplementation Normalized Body Weight, Blood Glucose Levels, Oxidative Stress Response, and Levels of Circulatory Inflammation Markers in STZ-Induced Diabetic Female Mice

We measured the blood glucose levels and body weights of the mice weekly to first confirm the onset of STZ-induced DM and then to examine the response to E_2_ therapy during the six-week treatment regimen (Table 2). We also measured the circulatory concentrations of insulin, nitrite, E_2_, MDA, and inflammatory cytokines using commercially available ELISA kits (Table 2). We observed that STZ-treated female mice were highly susceptible to weight loss and hyperglycemia (STZ vs. CON) (*p* < 0.05). Interestingly, while E_2_ administration did not yield significant weight gain, mice that received therapeutic E_2_ dosages were marginally heavier than mice in the STZ-only group. Therapeutic doses of E_2_ also significantly decreased glycemia (*p* < 0.05), though not to pre-diabetic or healthy levels.

We found circulatory estrogen levels to be significantly reduced (*p* < 0.05) upon STZ treatment (CON: 33.4 ± 3.3 vs. STZ: 20.7 ± 2.8 ng/L). In addition, we also found E_2_ supplementation to significantly elevate serum estrogen levels in a dose-dependent manner. These observations suggest that E_2_ dosages used in this study could restore serum estrogen levels in diabetic mice up to levels comparable to those closer levels in control mice (Table 2).

The insulin levels in STZ-induced diabetic female mice were also decreased. Increasing doses of E_2_ supplementation did not significantly restore their insulin levels. Total serum nitrite levels are indicative of NO production in systemic circulation, whereas MDA levels are indicative of levels of reactive oxygen species and oxidative load. As shown in Table 2, total serum nitrate levels were diminished in STZ-induced diabetic mice. E_2_ supplementation at therapeutic doses (0.25 mg/kg *b.w*. and 1.0 mg/kg *b.w*.) substantially elevated their systemic nitrite levels. MDA concentrations were significantly elevated in STZ-induced diabetic mice. Similarly, E_2_ supplementation at therapeutic doses restored their MDA levels to levels comparable to those in healthy mice. Inflammation and oxidative stress are common sequela in DM [14]. We observed a substantial level increase for pro-inflammatory cytokines IL-6, TNFα, and IGF-1 in the diabetic mice. Daily E_2_ supplementation at the two highest doses (0.25 mg/Kg and 1.0 mg/Kg) resulted in a statistically significant reduction in the levels of these cytokines (Table 2).

Taken together, our data suggest that daily E_2_ supplementation influenced the blood glucose, as well as the inflammation and oxidative stress load in STZ-induced diabetic female mice.

### 3.2. E_2_ Supplementation Restored Gastric Emptying and Nitrergic Relaxation in STZ-Induced Diabetic Female Mice

**Figure 1:**
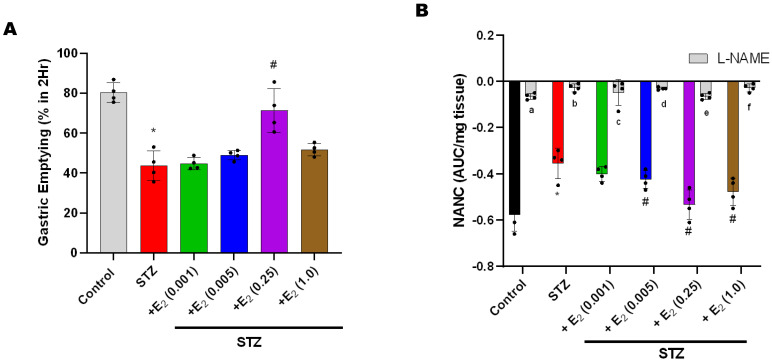
Effect of 17β-estradiol (E_2_) on solid gastric emptying and gastric antral nitrergic relaxation in STZ-induced diabetic female mice. The rate of GE was measured as follows: 1 − [(Gastric Content/Food Intake) × 100 (**A**). Full and empty stomach weights were recorded to estimate the remaining gastric content after a 2 h fast. Nitrergic relaxation was measured in gastric antral circular muscle strips at 2 Hz in an organ bath under physiological conditions (**B**). Nitric oxide (NO) dependence of NANC relaxation was confirmed by pre-incubation (30 min) with NO inhibitor nitro-L-arginine methyl ester (L-NAME; 100 μM). Area under the curve (AUC) per mg of tissue is presented. Data were analyzed with one-way ANOVA using GraphPad Prism. Data are means ± SEM (*n* = 6). * denotes *p* < 0.05; significantly different from healthy mice (Con). ^#^ denotes *p* < 0.05; significantly different from STZ-induced diabetic mice (STZ). ^a^ denotes *p* < 0.05 to control with L-NAME, ^b,c,d,e,f^ denotes *p* < 0.05 to respective groups with L-NAME. Legends: gastric emptying (GE), non-adrenergic, noncholinergic (NANC), nitric oxide (NO), nitro-L-arginine methyl ester (L-NAME), area under curve (AUC), analysis of variance (ANOVA), standard error of the mean (SEM), streptozotocin (STZ).

As shown in Figure 1A, STZ-induced diabetic female mice displayed significantly delayed solid GE compared to control mice (79% for CON vs. 42% for STZ, *p* < 0.05). E_2_ supplementation at 0.25 mg/kg restored GE to rates comparable to those in control mice (*p* < 0.05). E_2_ supplementation at other doses did not result in significant changes in the GE rates of the diabetic mice.

NO is the primary neurotransmitter fueling muscle relaxation in the gut. Low-frequency EFS (2 Hz) in an organ bath elicits inhibitory relaxation in the gastric antral neuro-musculature [35]. NANC inhibitory relaxation was severely impaired in STZ-induced diabetic mice, consistent with delayed solid GE. E_2_ supplementation restored NO-mediated relaxation in the gastric antrum of STZ-induced diabetic mice to levels comparable to those in healthy mice (*p* < 0.05, Figure 1B). Blockade of nNOS with L-NAME drastically diminished gastric relaxation in all mice (Figure 1B, grey bars). These results support the involvement of NO in this process. Taken together, our results show that E_2_ supplementation restored nitrergic function and GE in diabetic female mice to levels comparable to those in healthy mice.

### 3.3. E_2_ Supplementation Affected ERs and MAPK mRNA and Protein Levels in STZ-Induced Diabetic Female Mice

**Figure 2:**
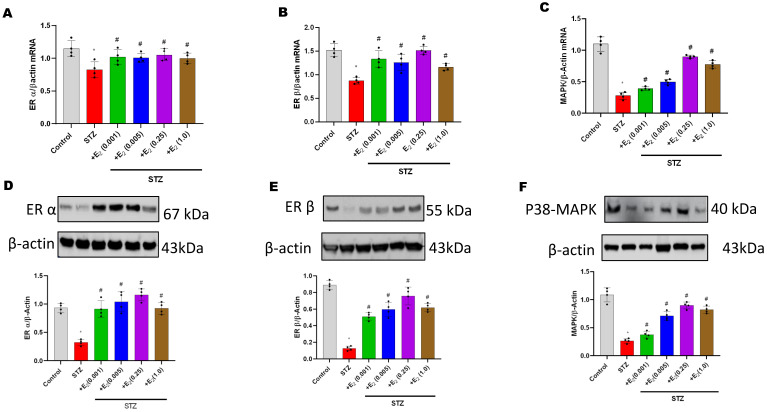
Effect of 17β-estradiol (E_2_) on mRNA and protein levels of estrogen receptor alpha (ERα), estrogen receptor beta (ERβ), and phospho-MAPK in gastric neuromuscular tissues of STZ-induced diabetic mice. Total RNA was isolated from the gastric antral tissues. qRT-PCR was used to measure the level of each indicated mRNA (**A**–**C**). Data presented were normalized to mRNA levels of the housekeeping gene β-actin. Representative immunoblot and densitometry analysis for ERα (**D**), ERβ (**E**), and *p*-MAPK (**F**) protein levels in gastric neuromuscular tissue samples are presented. Stripped blots were re-probed for β-actin. Densitometry data for target proteins were normalized to the protein levels of β-actin. Bar graphs depict the ratios between levels of target gene or protein to those of β-actin. Data were analyzed with one-way ANOVA using GraphPad Prism. Data are means ± SEM (*n* = 4). * denotes *p* < 0.05; significantly different from healthy mice (Con). ^#^ denotes *p* < 0.05; significantly different from STZ-induced diabetic mice (STZ). Legends: estrogen receptor alpha (ERα), estrogen receptor beta (Erβ, mitogen activated protein kinases (MAPK), streptozotocin (STZ), ribonucleic acid (RNA), real-time quantitative reverse transcription PCR (qRT-PCR), messenger ribonucleic acid (mRNA), analysis of variance (ANOVA), standard error of the mean (SEM).

It is widely accepted that estrogen receptor signaling regulates many biological effects primarily via two receptor subtypes, ERα and Erβ [36]. These receptors function as ligand-activated transcription factors and intracellular signaling agents that perform the actions of estrogens [36]. We observed a reduction in the mRNA and protein levels for both receptors in STZ-induced diabetic mice. E_2_ supplementation in the diabetic mice at all doses used restored their mRNA and protein levels for these receptors to levels comparable to those in control mice (Figure 2A,B,D,E).

We have previously shown diabetic conditions and E_2_ to influence mitogen-activated protein kinase (MAPK) activation and signaling [33,34]. Here, our findings demonstrate marked decreases (*p* < 0.05) in p38/MAPK mRNA and protein levels in STZ-treated female mice (Figure 2C,F). E_2_ supplementation upregulated p38/MAPK mRNA and protein levels in the diabetic mice in a dose-dependent trend, with the highest doses exhibiting expression levels comparable to those observed in healthy control mice (Figure 2C,F).

### 3.4. E_2_ Supplementation Restored the Levels of GSK-3β, Cytosolic and Nuclear Nrf2, and Phase II Antioxidant Enzymes to Normal Levels in STZ-Treated Diabetic Female Mice

**Figure 3:**
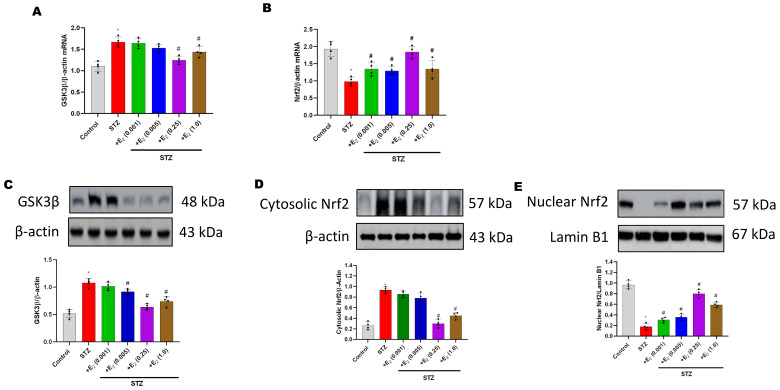

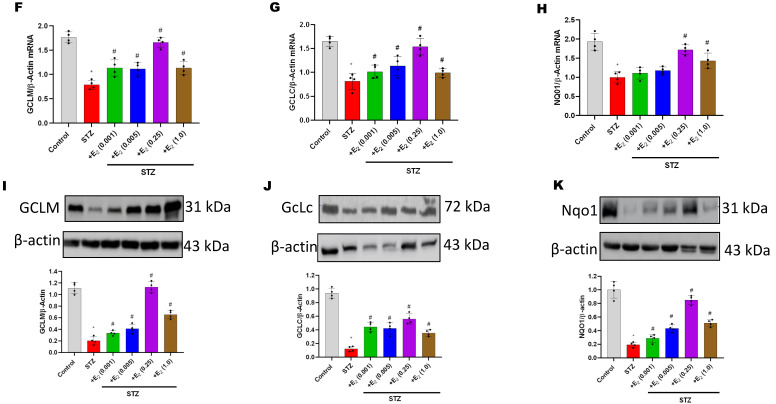
Effect of 17β-estradiol (E_2_) on levels of GSK-3β, cytosolic Nrf2, nuclear Nrf2, and Phase II enzymes in STZ-induced diabetic mice. Total RNA was isolated from the gastric antral tissues. qRT-PCR was used to measure the level each indicated mRNA. Data presented were normalized to the mRNA level of the β-actin gene (**A**,**B**,**F**–**H**). Representative immunoblot and densitometry analysis for protein levels of GSK-3β (**C**), cytosolic Nrf2 (**D**), nuclear Nrf2 (**E**), and Phase II enzymes (GCLC, GCLM, NQO1) (**I**–**K**) in gastric neuromuscular tissue. Stripped blots were re-probed for β-actin. Densitometry data for target proteins were normalized to the protein levels of β-actin. Bar graphs depict the ratios of levels of target genes or proteins to those of β-actin. Data were analyzed with one-way ANOVA using GraphPad Prism. Data are means ± SEM (*n* = 4). * denotes *p* < 0.05; significantly different from healthy mice (Con). ^#^ denotes *p* < 0.05; significantly different from STZ-induced diabetic mice (STZ). Legends: glycogen synthase kinase 3 beta (GSK-3β), nuclear factor erythroid 2-related factor 2 (Nrf2), streptozotocin (STZ), glutamate-cysteine ligase (GCL (catalytic subunit (**C**) and modifier subunit (M)), NAD(*p*)H quinone dehydrogenase 1 (NQO1) ribonucleic acid (RNA), real-time quantitative reverse transcription PCR (qRT-PCR), messenger ribonucleic acid (mRNA), analysis of variance (ANOVA), standard error of the mean (SEM).

Emerging research suggests that glycogen synthase kinase-3β (GSK-3β) participates in oxidative stress homeostasis through its interaction with nuclear factor erythroid 2 p45-related factor 2 (Nrf2) [37,38]. Here, we observed elevated levels of GSK-3β in STZ-treated mice when compared to control mice (Figure 3A,C). E_2_ supplementation reduced GSK-3β levels in STZ-treated mice (Figure 3A,C). Nrf2 activation and its downstream effect on Phase II enzymes are crucial to combating cellular oxidative stress. Cytosolic Nrf2 is typically bound to Keap-1 in an inactive state. Active Nrf2 traverses the nucleus in a redox-rich state [39]. Here, we found the levels of cytosolic and nuclear Nrf2 in gastric antrum samples to be inversely proportional (Figure 3D,E). Supplementing STZ-treated mice with therapeutic doses of E_2_ normalized the Nrf2 levels in both cellular compartments, implying at least a partial restoration of Nrf2 activity in these mice.

Hyperglycemia is associated with increased oxidative stress. Although estrogens have been shown to possess antioxidant properties, not much is known about their role in co-regulating Phase II enzymes with Nrf2. Figure 3F–K depict the effects of estrogens on Phase II enzymes GCLC, GCLM, and NQO1 in diabetic mice. E_2_ supplementation increased the levels of gastric Phase II enzymes GCLC, GCLM, and NQO1 to various extents, bringing their levels closer to those in healthy mice (Figure 3F–K).

### 3.5. E_2_ Supplementation Normalized the Levels of Gastric nNOSα and GCH-1 Proteins in Diabetic Mice

**Figure 4:**
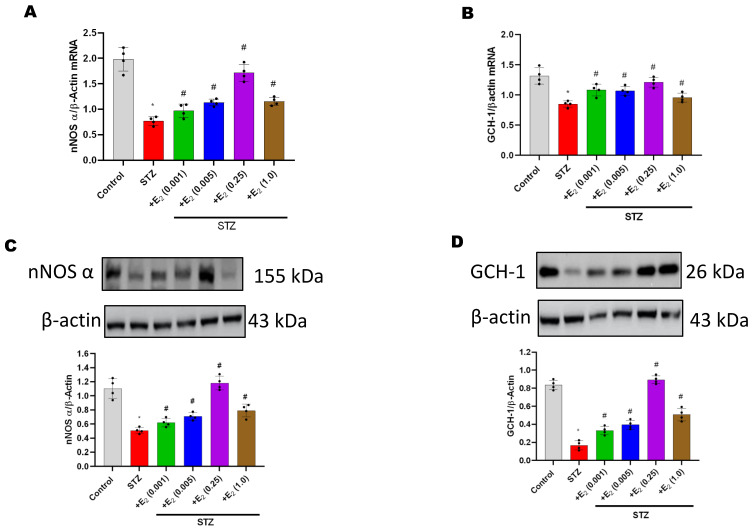
Effect of 17β-estradiol (E_2_) on levels of gastric nNOSα and GCH-1 in STZ-induced diabetic mice. Normalized mRNA levels (**A**,**B**), representative immunoblot, and densitometry analysis for nNOSα (**C**) and GCH-1 (**D**) are presented. Stripped blots were re-probed for β-actin. Data for levels of target genes or proteins were normalized to those of β-actin. Bar graphs depict ratios of levels of target genes or proteins to those of β-actin. Data were analyzed with one-way ANOVA using GraphPad Prism. Data are means ± SEM (*n* = 4). * denotes *p* < 0.05; significantly different from healthy mice (Con). ^#^ denotes *p* < 0.05; significantly different from STZ-induced diabetic mice (STZ). Legends: neuronal nitric oxide synthase (nNOS), GTP cyclohydrolase 1 (GCH-1) streptozotocin (STZ), analysis of variance (ANOVA), standard error of the mean (SEM).

NO-mediated relaxation has been reported as the predominant component that is severely compromised in female rodent models of diabetic Gp, specifically within the proximal regions of the stomach, colon, and intestinal smooth muscle [4,40,41]. Human and animal studies suggest that NO depletion due to nNOS dysfunction and expression may lead to delayed GE in diabetes and high oxidative stress conditions [4]. Since STZ-treated mice presented with depressed nitrergic relaxation in the gastric antrum, we measured their nNOS𝛼 levels in the presence and absence of E_2_ supplementation. We observed a marked decrease in nNOSα mRNA and protein levels in STZ-treated mice compared to control mice (Figure 4A,C). Interestingly, while nNOSα mRNA levels were elevated in the diabetic mice at all doses of E_2_ supplementation, nNOSα protein levels were significantly elevated (*p* < 0.05) only in diabetic mice receiving therapeutic doses of E_2_ (0.25 mg/kg and 1.0 mg/kg).

Tetrahydrobiopterin (BH_4_) is an essential cofactor for nNOS activity [42]. BH_4_ biosynthesis is chiefly regulated by two enzymes in converging pathways: GCH-1 (de novo) and DHFR (salvage). Our data demonstrate a decrease in GCH-1 expression in gastric antrum samples from STZ-induced diabetic female mice compared to control mice (Figure 4B,D). E_2_ supplementation significantly elevated GCH-1 protein expression (*p* < 0.05) in the diabetic mice, implying at least a partial restoration of BH_4_ biosynthesis (Figure 4D).

### 3.6. E_2_ supplementation Restored Levels of Nuclear NFκB and Gastric Pro-Inflammatory Cytokines in STZ-Treated Diabetic Mice to Levels Comparable to Those in Healthy Mice

**Figure 5:**
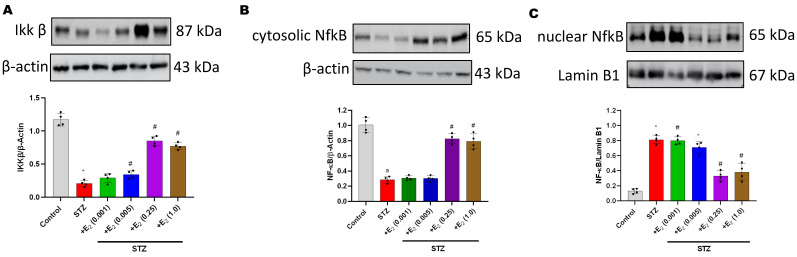

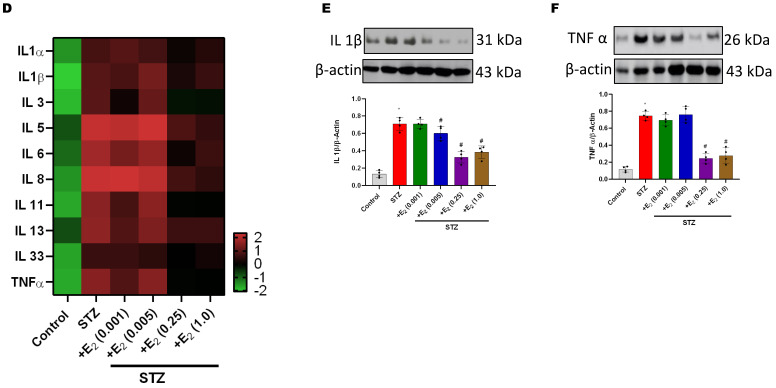
Effect of 17β-estradiol (E_2_) on levels of IkkB, NF-κB, and pro-inflammatory cytokines in STZ-induced diabetic mice. Representative immunoblot and densitometry analysis for IkkB (**A**), cytosolic and nuclear NF-κB (**B**,**C**), Heat map of differentially expressed inflammatory genes in control, STZ, and STZ mice receiving various doses of 17β-estradiol (E_2_) supplementation (**D**), IL-1β (**E**), and TNF α (**F**) in gastric neuromuscular tissues are presented. Stripped blots were re-probed for β-actin. Data for target proteins were normalized to protein levels of β-actin. Bar graphs depict ratios of levels of target proteins to those of β-actin. Data are presented as the average fold change of four mice per group at 9-week time point analyzed. Data were analyzed with one-way ANOVA using GraphPad Prism. Data are means ± SEM (*n* = 4). *^,a^ denotes *p* < 0.05; significantly different from healthy mice (Con). ^#^ denotes *p* < 0.05; significantly different from STZ-induced diabetic mice (STZ). Legends: inhibitor of nuclear factor kappa B kinase subunit beta (IkkB), nuclear factor kappa-light-chain-enhancer of activated B cells (NF-κB), tumor necrosis factor alpha (TNF)-α, interleukin 1 beta (IL)-1β, streptozotocin (STZ), analysis of variance (ANOVA), standard error of the mean (SEM).

Emerging evidence suggests that nuclear factor κB (NFκB) regulates the expression of various pro-inflammatory cytokines that influence gut function [43,44]. We observed a decrease in gastric IkkB and cytosolic NFκB levels in STZ-induced diabetic mice compared to healthy mice (Figure 5A,B). E_2_ treatment at 0.25 and 1.00 mg/kg restored the levels of gastric IkkB and cytosolic NFκB to levels comparable to those in healthy mice (Figure 5A–C). Conversely, nuclear NFκB levels were elevated in the gastric antrum of diabetic mice (Figure 5C). E_2_ treatment at therapeutic doses significantly reduced nuclear NFκB levels in diabetic mice to levels comparable to those in healthy mice. Furthermore, E_2_ restored the levels of several cytokines in diabetic female mice (Figure 5D) to levels comparable to those in healthy mice. Taken together, our observations suggest that E_2_ supplementation improved NFκB signaling and function in diabetic mice.

Next, we analyzed the levels of IL-1β and TNFα in the gastric antrum samples. Consistent with the increase in levels of nuclear NFκB, pro-inflammatory cytokines such as IL-1β and TNFα were also elevated in diabetic mice. E_2_ supplementation at therapeutic doses restored the levels of these cytokines in the diabetic mice to levels comparable to those in healthy mice (Figure 5E,F).

### 3.7. E_2_ Supplementation Affected the Expression of Apoptotic Markers Bax, BCL-2, and Caspase 3 in STZ-Induced Diabetic Female Mice

**Figure 6:**
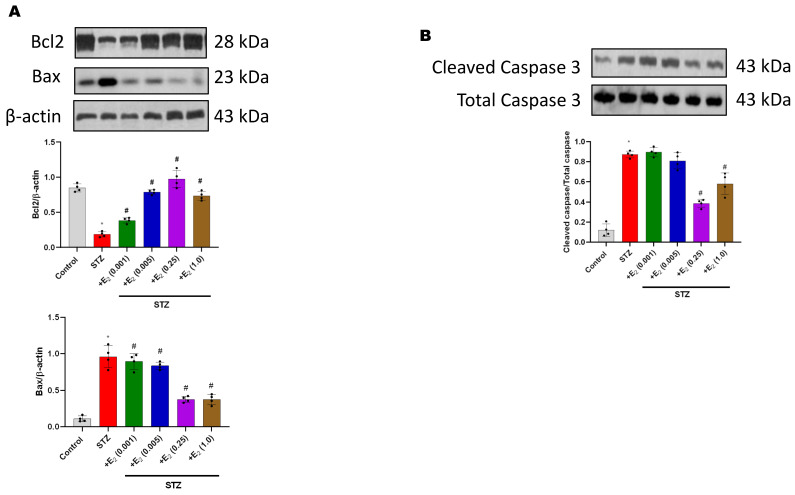
Effect of 17β-estradiol (E_2_) on levels of gastric BCL-2, BAX, and cleaved caspase 3 in STZ-induced diabetic mice. Representative immunoblot and densitometry analysis for BCL2 (**A**, **top**), BAX (**A**, **bottom**), and cleaved caspase 3 (**B**) in gastric neuromuscular tissues are presented. Data for protein levels of cleaved caspase 3 were normalized to protein levels of total caspase 3. Stripped blots were re-probed for β-actin. Data for BCL2 and BAX were normalized to β-actin protein levels. Bar graphs for BCL-2 and BAX depict the ratios of levels of these proteins to protein levels of β-actin protein. Bar graph for cleaved caspase 3 depicts ratios of cleaved caspase 3 protein levels to total caspase 3 protein levels. Data were analyzed with one-way ANOVA using GraphPad Prism. Data are means ± SEM (*n* = 4). * denotes *p* < 0.05; significantly different from healthy mice (Con). ^#^ denotes *p* < 0.05; significantly different from STZ-induced diabetic mice (STZ). Legends: B-cell lymphoma 2 (BCL-2), Bcl-2-associated X protein (BAX), streptozotocin (STZ), analysis of variance (ANOVA), standard error of the mean (SEM).

Excessive apoptosis of gastric cells is common in Gp patients with diabetic enteropathy [45]. B-cell lymphoma 2 (BCL-2) and Bcl-2-associated X protein (BAX) have been reported as pro- and anti-inflammatory regulators, respectively [46]. Here, we examined the protein levels of BCL-2, BAX, and caspase 3 in mouse gastric tissues. We observed a diminished level of BCL2 protein, the anti-apoptotic marker, in diabetic mice compared to healthy controls. E_2_ supplementation reversed this effect (Figure 6A). Conversely, the level for BAX protein, the pro-apoptotic marker, was elevated in diabetic mice compared to healthy controls. E_2_ supplementation at therapeutic doses reversed this effect (Figure 6A). Figure 6B illustrates the protein levels of gastric caspase 3. The level of cleaved caspase 3 was elevated in diabetic mice; E_2_ supplementation reversed this effect. Taken together, these observations indicate an increase in apoptosis in the gastric tissues of diabetic mice and that E_2_ supplementation at least partially reversed this effect.

## 4. Discussion

Gp is a chronic stomach motility disorder and common chief complaint among diabetic women [7]. It constitutes abnormal GE of solids and/or liquids in the absence of mechanical obstruction. Patients often suffer from debilitating symptoms, nutrition deficiencies, and overall decreased quality of life.

Diabetic women make up roughly 80% of the diabetic Gp patient population [47]. Several proposed reasons for this phenomenon include intrinsically slower GE rates in females, elevated levels of sex steroid hormones, and the loss of nNOS expression and interstitial cells of Cajal (ICC); however, the molecular mechanism of disease occurrence and progression remains a mystery. The purpose of our present study was to elucidate the effects of E_2_ on nNOS-mediated gastric motility as well as the inflammation and apoptotic cascades in a mouse model of diabetic Gp. We demonstrated that daily E_2_ supplementation can improve metabolic and oxidative stress homeostasis as well as nNOS-mediated gastric motility in diabetic mice. Our findings suggest that E_2_ plays a protective role in diabetic gastric motility in female rodents.

DM is a metabolic disease associated with several intestinal disorders [48]. Rodent STZ-induced diabetic models have proven extremely useful in studying the DM pathology in mice and the potential effects of hormone supplementation [31,49,50,51]. STZ is an antibiotic that causes pancreatic islet β-cell destruction and is widely used experimentally to induce type 1 DM in rodents. These animals have been used extensively to demonstrate the effects of diabetes in several organ systems, including the stomach and GI tract [31]. In our study, we treated female mice with STZ to recapitulate several known metabolic characteristics in diabetes, including hyperglycemia, weight loss, depletion in insulin and estrogen levels, and elevation in the level of oxidative stress marker MDA [49,51,52,53]. Interestingly, women report several disruptions in sex hormone balance in diabetes [54,55]. Despite numerous findings that diabetes and hyperglycemia may influence estrogen levels in type 1 DM patients, very few studies evaluate circulating levels in rodent research models utilizing STZ. Our findings in female C57/BL6 mice suggest a significant decline in serum estradiol levels after diabetes induction with STZ, correlating with another study of diabetic nephropathy in Sprague-Dawley rats [56]. However, conflicting reports exist on the effects of STZ-induced diabetes on serum estradiol levels in rodents. Here, we provide our findings of this data in C57/BL6 mice that STZ-induced diabetes is consistent with a diminished serum estradiol level in mice, similar to the findings in human data [57]. Future studies geared towards nullifying the effects of ovarian hormones, estrogen and progesterone, and subsequent re-supplementation in the type 1 diabetic mouse gastric system will be important to our field.

Diabetic enteropathy causes GI autonomic nerve dysfunction, consequently disrupting ENS activity and function [45]. GE requires synchronous excitatory contractions and NO-mediated inhibitory relaxation of gastric musculature along the GI apparatus. NO is a crucial neurotransmitter that regulates the inhibitory plexus of gastrointestinal motility and transit time [58]. Here, we observed decreased NO-mediated nitrergic relaxation in gastric antrum specimens from diabetic mice that corresponded to delayed GE. Additionally, the levels of nNOSα and GCH-1 were diminished in STZ-treated mice compared to healthy mice. E_2_ supplementation restored levels of nNOSα and GCH-1 as well as nitrergic relaxation and GE in the diabetic mice. These observations align with our previous findings in mice with high-fat diet-induced type 2 diabetes [16]. Taken together, our results suggest that E_2_ can improve nNOS function via BH_4_ synthesis in both type 1 and 2 DM female mice.

Estrogen and its chief receptors are known to impact processes of the gastrointestinal tract in experimental animal models and in humans [21,36]. In the gastric and small intestinal mucosa, estrogen receptors facilitate genomic and non-genomic intracellular activity. In vitro studies have shown that estrogen can affect the contractile response and myoelectric activity of gastrointestinal smooth muscle [24]. Furthermore, E_2_ exerts both genomic and non-genomic effects on nNOS/NO abundance in vascular endothelium dilation [26,59,60]. The mechanisms responsible for these effects are not completely understood, and conflicting reports exist in the literature. Several studies have implicated increased pyloric ERβ in the development of GP in STZ-induced male diabetic rats [51]. Here, we demonstrated the effects of E_2_ supplementation on GE in STZ-induced diabetic female mice with Gp. Supplemental E_2_ at various doses improved GE in these mice by improving nitrergic relaxation. It is postulated that this system is often the most severely compromised in diabetic human patients.

We previously showed E_2_ to crosstalk with MAPK. The MAPK pathway bridges the switch from the receipt of extracellular signals to the initiation and progression of intracellular responses under both healthy and inflammatory conditions; the latter includes diabetes [61,62]. Previous studies have shown that p38/MAPK may interact with ER upon ligand binding to influence intracellular function. Here, we found the levels of gastric MAPK, ERα, and ERβ to be suppressed in diabetic mice, and E_2_ supplementation restored their levels to levels comparable to those in healthy mice. Furthermore, enhanced MAPK signaling has been shown to positively correlate with a depletion in inflammatory cytokines with elevated antioxidants, resulting in reduction of oxidative stress [18]. Our results corroborate with this finding because we found E_2_ supplementation to normalize the levels of oxidative stress marker MDA, Nrf2, Phase II enzymes (GCLC, GCLM, and NQO1), and serum and gastric inflammatory markers. It is noteworthy that E_2_ can also protect against oxidative stress in tissues expressing ERβ due to induction of GCLM. While the mechanism of the MAPK/E_2_ crosstalk is unclear, we have demonstrated their relationship in two animal models of diabetes [16]. Therefore, the potential role for both MAPK and E_2_ in regulating oxidative stress and inflammation warrants further investigation.

Oxidative stress is caused by imbalance between reactive oxygen species (ROS) and the antioxidant response system. It is a plausible etiologic factor that underlies the loss of nitrergic function in diabetic patients [17,39]. DM induces a state of high oxidative stress that affects various tissues. MDA is considered an indirect marker of oxidative stress [63]. Here, we observed the MDA levels to be significantly increased in diabetic mice, which was consistent with previously reported findings [64]. Nrf2 is an oxidative stress-sensitive transcription factor that regulates cellular protection against oxidative stress by activating an array of antioxidant response genes [65]. GSK-3β and Nrf2 have emerged as promising therapeutic targets for treating chronic diseases including several nervous system disorders and diabetes, due to their role in the cellular response to oxidative stress [37]. We have previously reported that Nrf2 loss in female mice resulted in elevated gastric GSK-3β levels, decreased tetrahydrobiopterin (BH_4_) levels, inhibition of neuronal NO (nNOSα, nitrergic neuron)-mediated gastric relaxation, and reduction in nitrite levels, subsequently leading to delayed GE [34]. Similarly, we found GSK-3β upregulation to cause delayed GE by reducing levels/activity of gastric PI3K/Akt/Nrf2, Phase II enzymes, and BH_4_-nNOSα in an obesity-induced diabetes mouse model [19]. It has been proposed that GSK-3β regulates Nrf2 translocation to the nucleus. Nrf2 phosphorylation by GSK-3β leads to nuclear exclusion and degradation, consequently derailing antioxidant stress response and Phase II enzyme induction. Studies from Pandy and colleagues suggest that estrogen supplementation inhibits GSK3 activity in the hippocampal neurons of ovariectomized rodents [63,66]. Interestingly, GSK-3β has also been shown to have a dual inhibition and activation effects on Nrf2 via NFκB [38,43]. Though several studies have reported diverging findings on the influence of estrogens on Nrf2 in various cell types, information on their manifestations in diabetic female mouse models is scant. Here, we observed an elevation in GSK-3β levels in diabetic female mice, which we could reverse with therapeutic doses of E_2_ supplementation. When GSK-3β levels were reduced in diabetic mice, so were the levels of cytosolic Nrf2 levels. We also found E_2_ supplementation to restore expression of Nrf2 and increase the levels of NQO1, GCLC, and GCLM in diabetic mice. NQO1 is a Nrf2-dependent quinone reductase enzyme that alleviates oxidative stress by acting as a superoxide reductase to modulate redox balance [67]. Another Nrf2-dependent Phase II enzyme is glutamate-cysteine ligase (GCL), a rate-limiting enzyme in glutathione synthesis composed of a catalytic subunit (GCLC) and a modifier subunit (GCLM) [68]. Our findings show the increase in nuclear Nrf2 levels upon E_2_ supplementation to positively correlate with the increase in the levels of these Phase II enzymes.

Importantly, our data show that E_2_ can repress hyperglycemia (HG), Nrf2 loss, and induction of apoptosis by decreasing levels of pro-inflammatory cytokines and reactive oxygen species (ROS). Recent studies have proposed the activation of NFκB signaling to be important in apoptosis of ICC in GI transit disorders [69,70,71,72]. NFκB represents a family of transcription factors that, when activated, regulates more than 400 various genes such as enzymes, cytokines, cell-cycle regulatory molecules, angiogenic factors, etc. These important transcription elements are normally held dormant in the cytoplasm by inhibitory molecules of the IκB family [43]. NFκB is activated by a wide variety of agents, including oxidative stress, inflammatory stimuli, cytokines, and endotoxins. When activated, NFκB regulates more than 400 various genes such as enzymes, cytokines, cell-cycle regulatory molecules, angiogenic factors, etc. [43,73]. NFκB has been linked to numerous human diseases, most notably diabetes. NF-κB is activated by IKK kinase, which phosphorylates and inhibits IKB-α, the endogenous inhibitor of NF-κB. Levels of IkkB and cytosolic NFκB were reduced in STZ-induced diabetic mice, whereas nuclear NFkB level was upregulated. E_2_ supplementation reversed these effects, corroborating data observed in starved MLO-Y4 cells [74]. Recent studies suggest Nrf2 and Phase II enzymes play a key part in inhibiting NFκB signaling. The Nrf2 and NFκB pathways have also been proposed to exert an inhibitory effect on each other at the transcription level [65,73]. Nuclear translocation of NFκB results in the secretion of pro-inflammatory cytokines, including tumor necrosis factor-a (TNF-α), interleukin 6 (IL-6), interleukin 10 (IL-10), and interleukin-1beta (IL-1β) [73,75,76]. Here, we found therapeutic doses of E_2_ supplementation restored the gastric and systemic levels of pro-inflammatory markers (IL-1β and TNFα) to healthy, pre-stress levels.

Inflammation overload, combined with oxidative stress, can activate intrinsic and extrinsic apoptotic pathways in various tissues and organ systems [77]. Diabetic autonomic neuropathy (DAN) is well-documented in the progression of long-term diabetes. This complication manifests in multiple symptoms that involve the gastrointestinal tract, including DGp, gall bladder atony, diabetic enteropathy, and colonic hypomotility [45]. The hallmark of DAN is rampant apoptosis in neurons throughout the body. The BCL-2 family consists of proteins that regulate apoptosis. Particularly, BCL-2 suppresses cell death while BAX promotes cell death [46]. Here, we observed diminished BCL-2 and exaggerated BAX expression levels in gastric antrum samples from diabetic female mice. Conversely, we observed higher BCL-2 expression and lower BAX expression in healthy mice and diabetic mice receiving pharmacological doses of E_2_. Our findings suggest a connection between ERs and the apoptotic machinery. We also found E_2_ supplementation to restore TNF-α levels in diabetic mice to healthy, pre-stress levels. TNF-α is involved in caspase-mediated apoptosis in ICC [78,79]. Therefore, while several mechanisms may govern the apoptotic response within the GI apparatus in our mouse model of diabetic GP, our findings suggest that E_2_ supplementation can modulate and at least partially restore the function of these mechanisms to healthy, pre-stress conditions.

The pathogenesis of DGp in men and women appears to be similar. However, women are consistently prone to be symptomatic due to the lower baseline kinetics of their stomachs, perhaps due to elevated levels of sex steroid hormones and inhibitory nitric oxide [10,13]. In this study, we showed E_2_ to influence apoptosis and oxidative stress in female mice with DGp.

Estrogen levels fluctuate in females, with physiological elevations and depressions occurring throughout the estrus cycle [25]. Furthermore, many hormones have tissue-specific functions and are most efficient at their optimal concentrations. In this study, we chose the supplemental E_2_ doses based on previous reports of circulating E_2_ levels in healthy mice [28,32,33,80]. We found the effect of physiological E_2_ doses (0.001 mg/kg *b.w*. and 0.005 mg/kg *b.w*.) to be largely minimal in restoring gastric function in diabetic mice. The depletion of endogenous estrogen was prevalent in STZ-induced diabetic mice, and physiological doses of E_2_ supplementation could not restore their estrogen levels (Table 2). On the other hand, pharmacologic doses of E_2_ (0.25 mg/kg *b.w*. and 1.0 mg/kg) normalized circulating estrogen levels in the diabetic mice to those observed in healthy mice. Thus, these E_2_ doses could alleviate some effects of oxidative stress, gastric apoptosis, and inflammation in order to improve GE in the diabetic mice. Interestingly, the pharmacologic doses of estradiol can be extrapolated to a human equivalent dose such as those contained in transdermal, oral, and ultra-low dose vaginal preparations [81,82,83]. Collectively, our findings indicate E_2_ supplementation at appropriate doses as a potential treatment regimen for diabetes-induced gastric dysmotility in women.

## 5. Conclusions

DGp is a debilitating condition with limited therapeutic options [15,80]. Current treatments for Gp include extreme dietary modifications, costly oral drug therapy, and invasive surgery [6]. As women are four times more likely to experience the debilitating symptoms of diabetic Gp, we hypothesize that this sex-related difference is due to the influence of female sex hormones on the gastro-motility milieu. Our current study uncovered new information that links the molecular underpinnings of inflammation, oxidative stress, and apoptotic response in Gp to E_2_-mediated activation of intracellular ER/MAPK/Nrf2/nNOSα signaling to improve GE. We conclude that changes in ER abundance and the downstream influence of target genes may precipitate the advancement and prognosis of Gp in female mice with type 1 DM. Due to the complexity of gut motility signaling in males versus females, further studies are needed to tease out the role of ERs, Nrf2, and inflammatory cytokines in experimental models of Gp.

## Figures and Tables

**Table 1:** Primer sequences for qRT-PCR.

Gene	Forward	Reverse	Accession Number
nNOS α	5′-CCCAACGTCATTTCTGTCCGT-3′	5′-TCTACCAGGGGCCGATCATT-3′	NM_008712
ER α	5′-CCCGCCTTCTACAGGTCTAAT-3′	5′-CTTTCTCGTTACTGCTGGACAG-3′	NM_007956
ER β	5′-CTGTGATGAACTACAGTGTTCCC-3′	5′-CACATTTGGGCTTGCAGTCTG-3′	NM_207707
GCH-1	5′-GAGCATCACCTTGTTCCATTTG-3′	5′-GCCAAGTTTACTGAGACCAAGGA-3′	NM_008102
β-actin	5′TGGAATCCTGTGGCATCCATGAAAC-3′	5′-TAAAACGCAGCTCAGTAACAGTCCG-3′	NM_007393
Nrf2	5′-TCTCCTCGCTGGAAAAAGAA-3′	5′-TAAAGCACAGCCAGCACATT-3′	NM_010902
GCLM	5′-GCCCGCTCGCCATCTCTC-3′	5′-GTTGAGCAGGTTCCCGGTCT-3′	NM_008129
GCLC	5′-ATGTGGACACCCGATGCAGTATT-3′	5′-TGTCTTGCTTGTAGTCAGGATGGTTT-3′	NM_010295
Nqo1	5′-GCCGAACACAAGAAGCTGGAAG-3′	5′-GGCAAATCCTGCTACGAGCACT-3′	NM_008706
P38MAPK	5′-AGGGCGATGTGACGTTT-3′	5′-CTGGCAGGGTGAAGTTGG-3′	NM_001168508
GSK-3β	5′-GCATTTATCATTAACCTAGCACCC-3′	5′-ATTTTCTTTCCAAACGTGACC-3′	NM_019827
IL 1α	5′-ACGGCTGAGTTTCAGTGAGACC-3′	5′-CACTCTGGTAGGTGTAAGGTGC-3′	NM_010554
IL 1β	5′-TGGACCTTCCAGGATGAGGACA-3′	5′-GTTCATCTCGGAGCCTGTAGTG-3′	NM_008361
IL 3	5′-CCTGCCTACATCTGCGAATGAC-3′	5′-GAGGTTAGCACTGTCTCCAGATC-3′	NM_010556
IL 5	5′-GATGAGGCTTCCTGTCCCTACT-3′	5′-TGACAGGTTTTGGAATAGCATTTCC-3′	NM_010558
IL 6	5′-TACCACTTCACAAGTCGGAGGC-3′	5′-CTGCAAGTGCATCATCGTTGTTC-3′	NM_031168
IL 8	5′-GAGAGTGATTGAGAGTGGACCAC-3	5′-CACAACCCTCTGCACCCAGTTT-3′	NM_000584
IL 11	5′-CTGACGGAGATCACAGTCTGGA-3′	5′-GGACATCAAGTCTACTCGAAGCC-3′	NM_001290423
IL 13	5′-AACGGCAGCATGGTATGGAGTG-3′	5′-TGGGTCCTGTAGATGGCATTGC-3′	NM_008355
IL 33	5′-CTACTGCATGAGACTCCGTTCTG-3′	5′-AGAATCCCGTGGATAGGCAGAG-3′	NM_001164724
TNF α	5′-GGTGCCTATGTCTCAGCCTCTT-3′	5′-GCCATAGAACTGATGAGAGGGAG-3′	NM_001278601

**Table 2:** Serum biochemical parameters analyzed in female mice at 9 weeks.

	Control	STZ	STZ + E_2_ (0.001 mg/Kg)	STZ + E_2_ (0.005 mg/Kg)	STZ + E_2_ (0.25 mg/Kg)	STZ + E_2_ (1.0 mg/Kg)
Body weight (g)	22.9 ± 0.5	18.8 ± 0.9	18.3 ± 0.8	19.0 ± 0.6	20.3 ± 0.8	19.7 ± 1.0
Blood glucose (mg/DL)	106 ± 07	441 ± 38 ^a^	446 ± 38	421 ± 24	324 ± 17 ^b^	389 ± 42 ^b^
Serum Insulin (ng/mL)	0.44 ± 0.04	0.24 ± 0.03	0.25 ±0.05	0.28 ± 0.04	0.31 ± 0.04	0.28 ± 0.05
Serum nitrate (µM)	31.5 ± 4.1	20.1 ± 3.3 ^a^	20.5 ± 3.1	20.8 ± 2.2	28.5 ± 3.5 ^b^	25.6 ± 2.1 ^b^
Serum Estradiol (ng/L)	33.4 ± 3.3	20.7 ± 2.8 ^a^	23.2 ± 3.4	25.6 ± 4.5 ^b^	34.8 ± 2.7 ^b^	43.6 ± 5.1 ^b^
Serum MDA (nmol/mg protein)	14 ± 2.4	42 ± 5.5 ^a^	40 ± 4.8	41 ± 5.1	20 ± 3.7 ^b^	28 ± 5.1 ^b^
Serum IL-6 (ng/mL)	88 ± 6	435 ± 32 ^a^	414 ± 28	404 ± 35	136 ± 15 ^b^	221 ± 44 ^b^
Serum TNF (ng/mL)	6 ± 0.6	22 ± 3 ^a^	21 ± 1.5	18 ± 2.4	10 ± 2.8 ^b^	14 ± 1.6 ^b^
Serum IGF-1 (ng/mL)	12 ± 1.2	33 ± 3.5 ^a^	30 ± 2.2	26 ± 3.1	15 ± 1.8 ^b^	22 ± 2.7 ^b^

^a^ *p* < 0.05 compared with control mice; ^b^ *p* < 0.05 compared with STZ-treated mice.

## Data Availability

All data sets presented in this study are available in the Supplementary Materials.

## Supplementary Materials

The following supporting information can be downloaded at: https://www.mdpi.com/article/10.3390/antiox12030758/s1.
