# Supplementary material for: 17β-Estradiol Suppresses Gastric Inflammatory and Apoptotic Stress Responses and Restores nNOS-Mediated Gastric Emptying in Streptozotocin (STZ)-Induced Diabetic Female Mice
Source: Antioxidants (Basel). 2023 Mar 20;12(3):758. doi: 10.3390/antiox12030758 (PMC10045314; doi:10.3390/antiox12030758)
Supplement: Supplementary file 1 [file antioxidants-12-00758-s001.zip › antioxidants-2228347-supplementary.pdf]

Supplementary files

Sprouse et al

Figure S2D
ER α

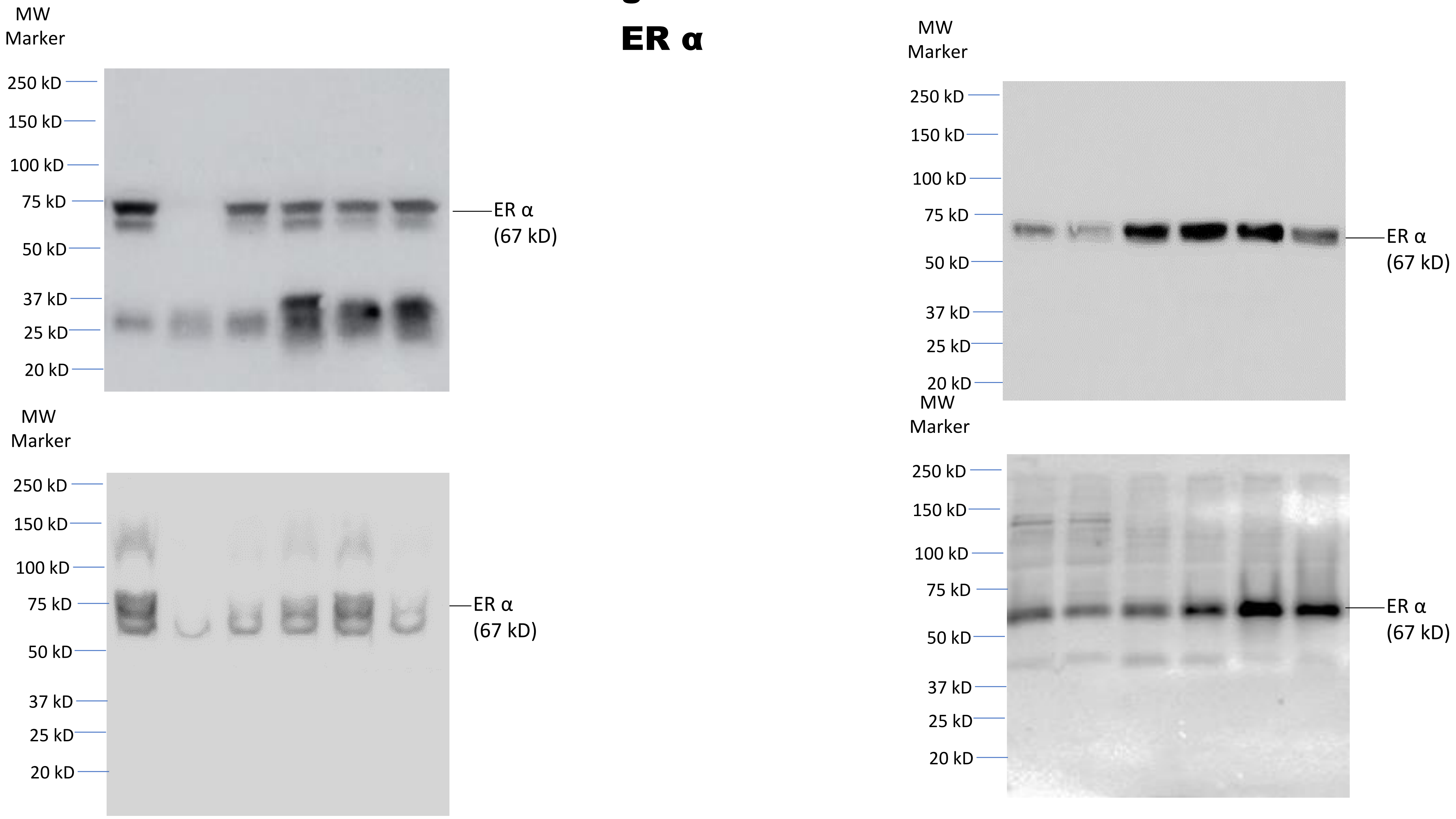

Figure S2D

β-Actin for Er α

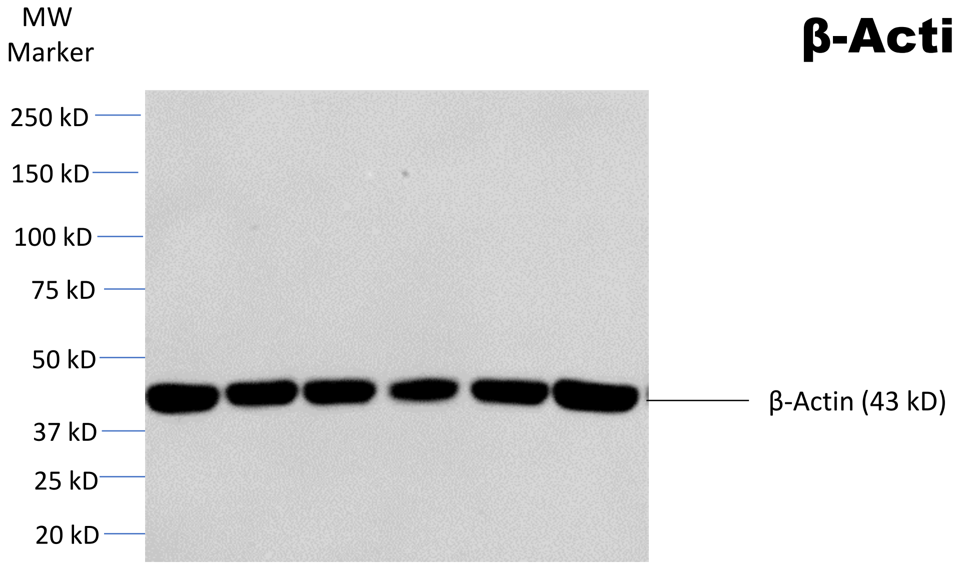

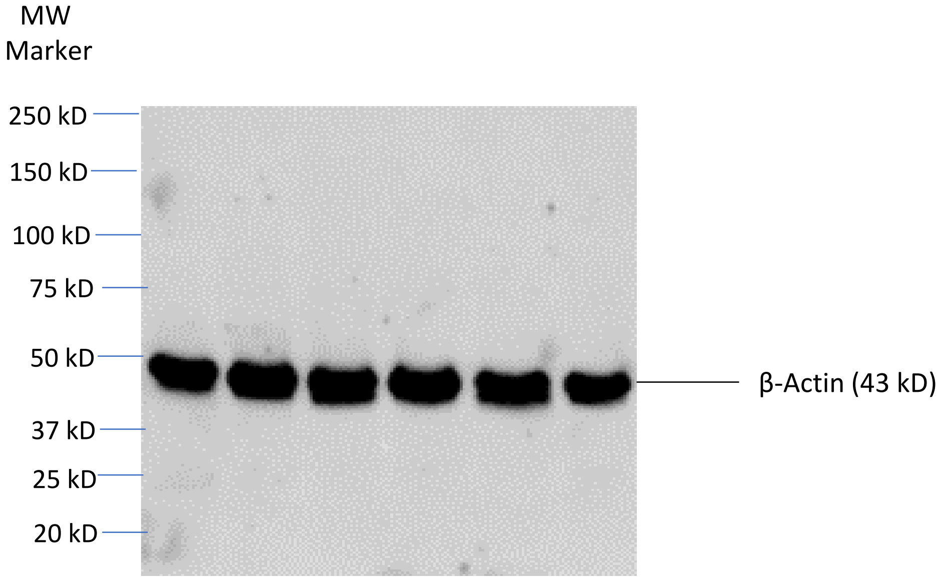

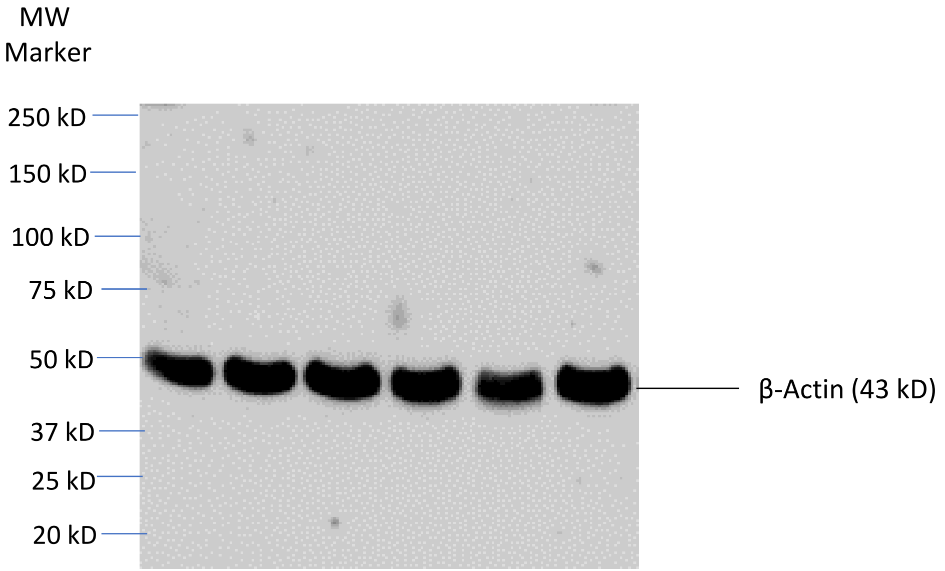

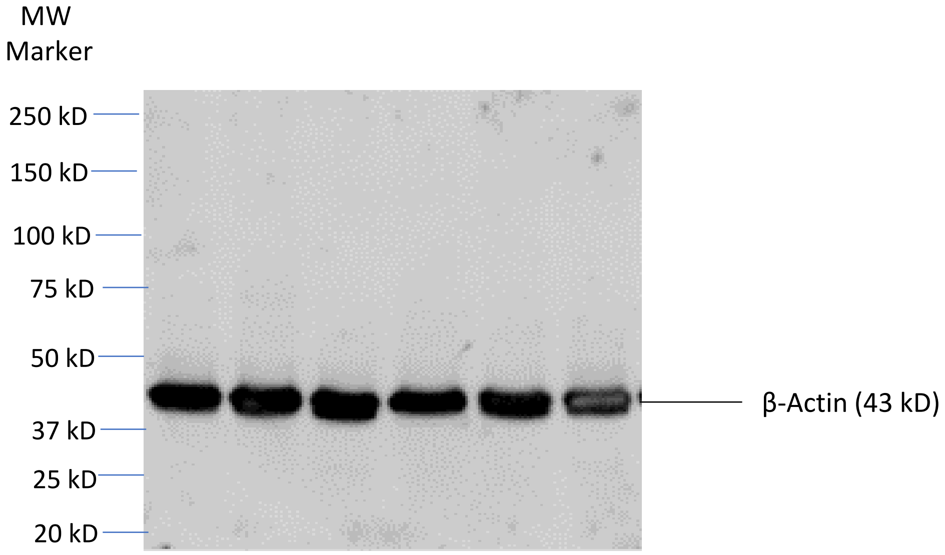

Figure S2E

ER β

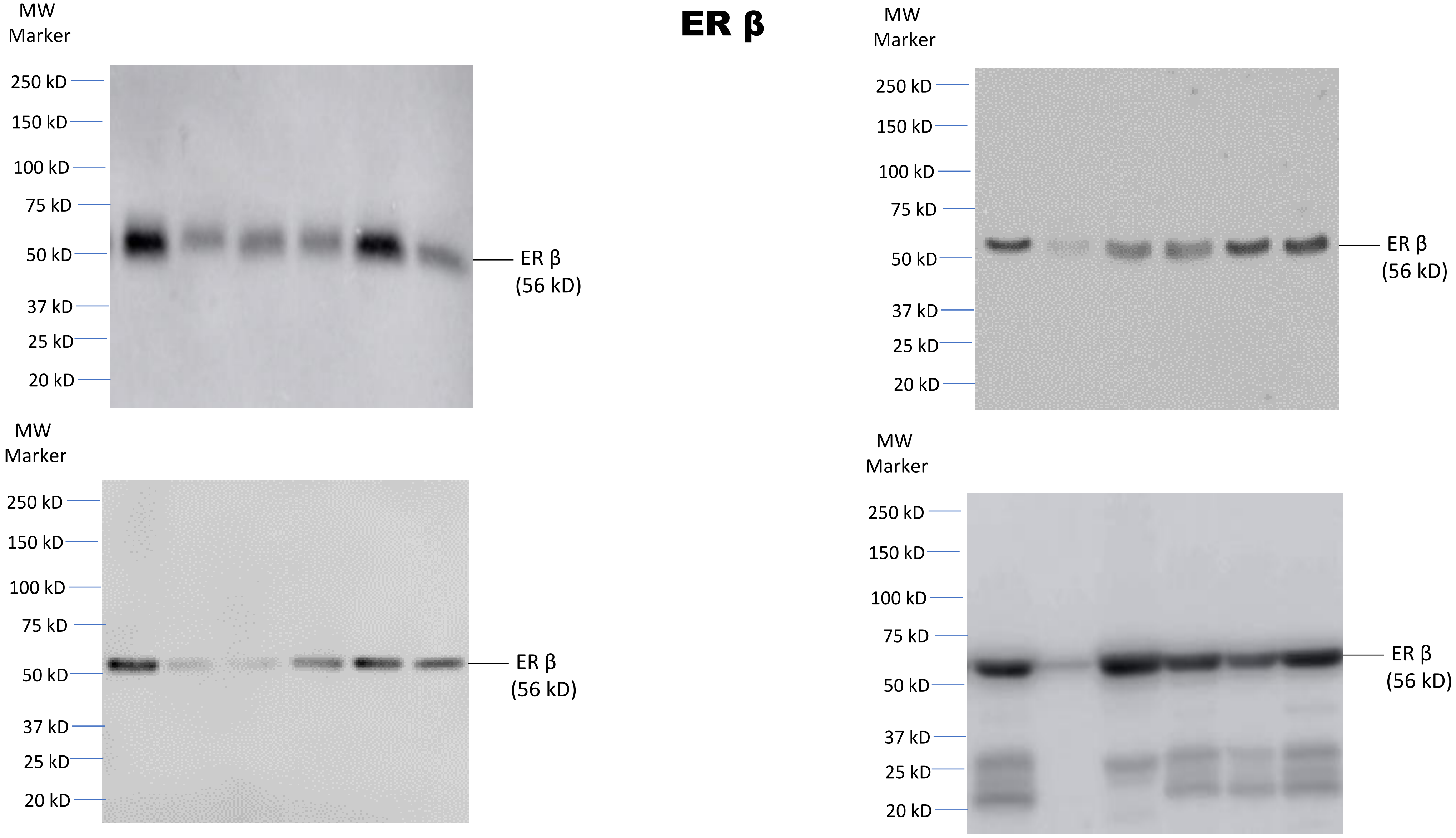

Figure S2E

β-Actin for Er β

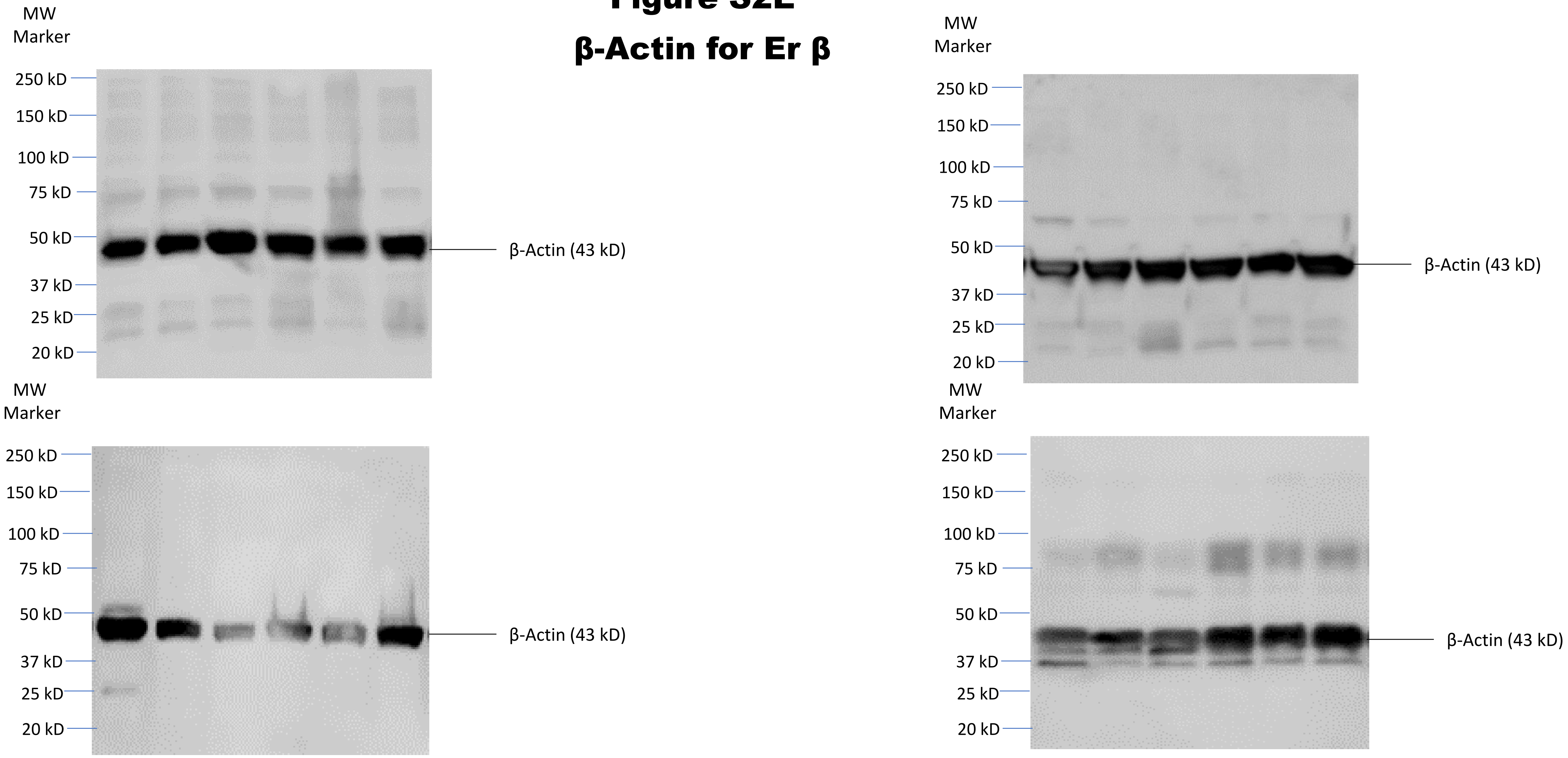

Figure S2F

p^{38}MAPK

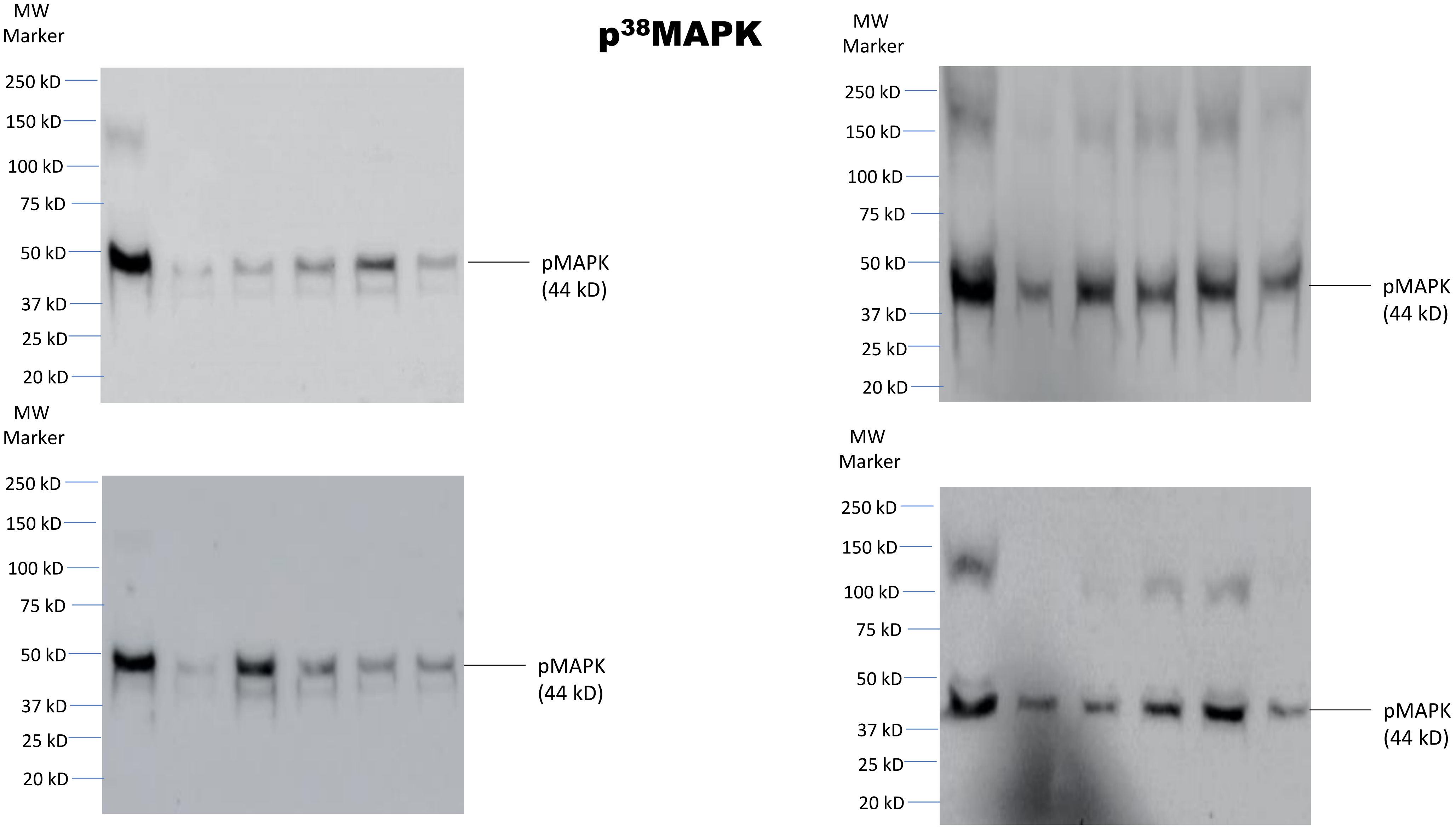

Figure S2F
β-Actin for p^{38}MAPK

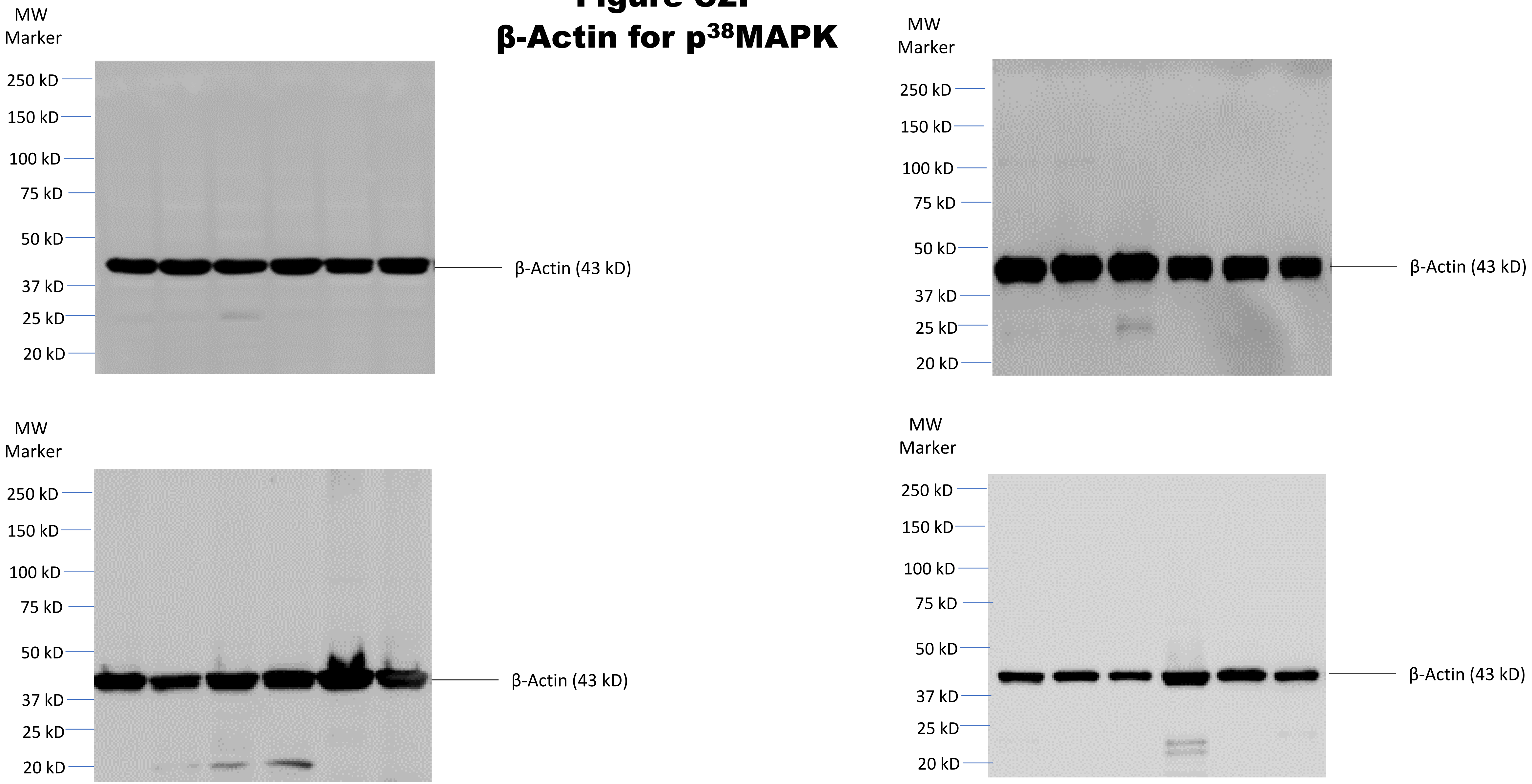

Figure S3C

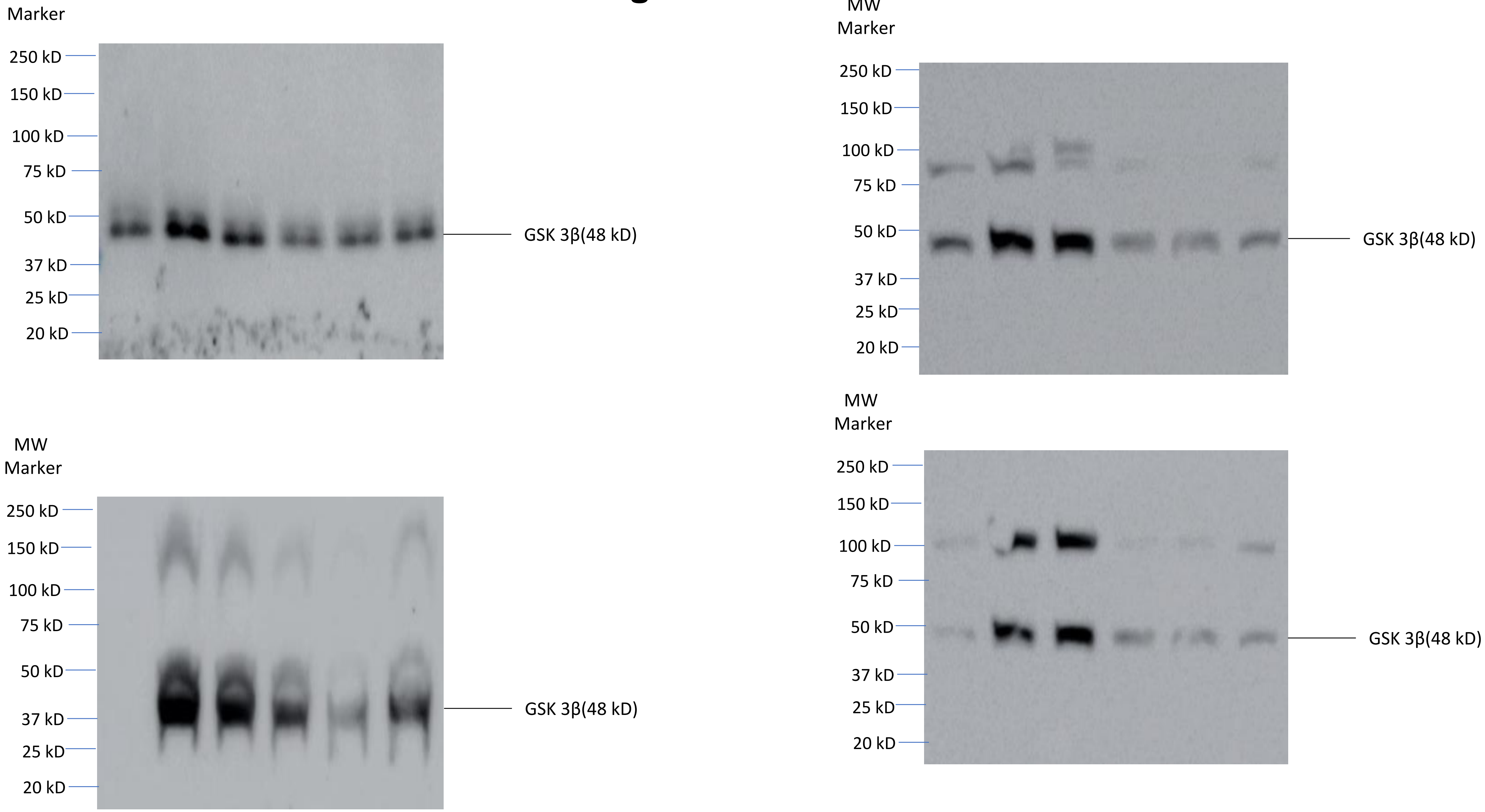
MW Marker
250 kD
150 kD
100 kD
75 kD
50 kD
37 kD
25 kD
20 kD
GSK 3β(48 kD)
MW Marker
250 kD
150 kD
100 kD
75 kD
50 kD
37 kD
25 kD
20 kD
GSK 3β(48 kD)
MW Marker
250 kD
150 kD
100 kD
75 kD
50 kD
37 kD
25 kD
20 kD
GSK 3β(48 kD)
MW Marker
250 kD
150 kD
100 kD
75 kD
50 kD
37 kD
25 kD
20 kD
GSK 3β(48 kD)

Figure S3C
β-Actin for GSK-3β

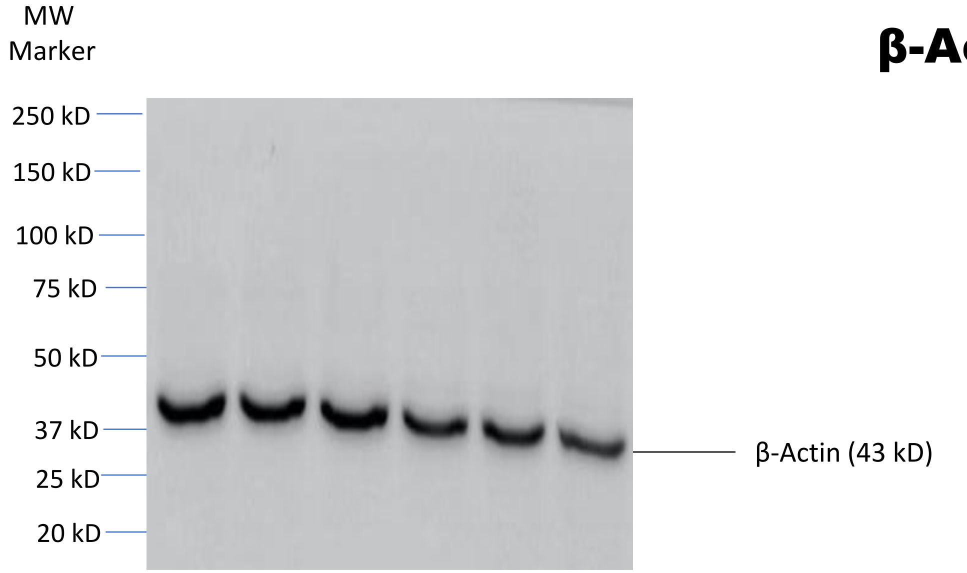

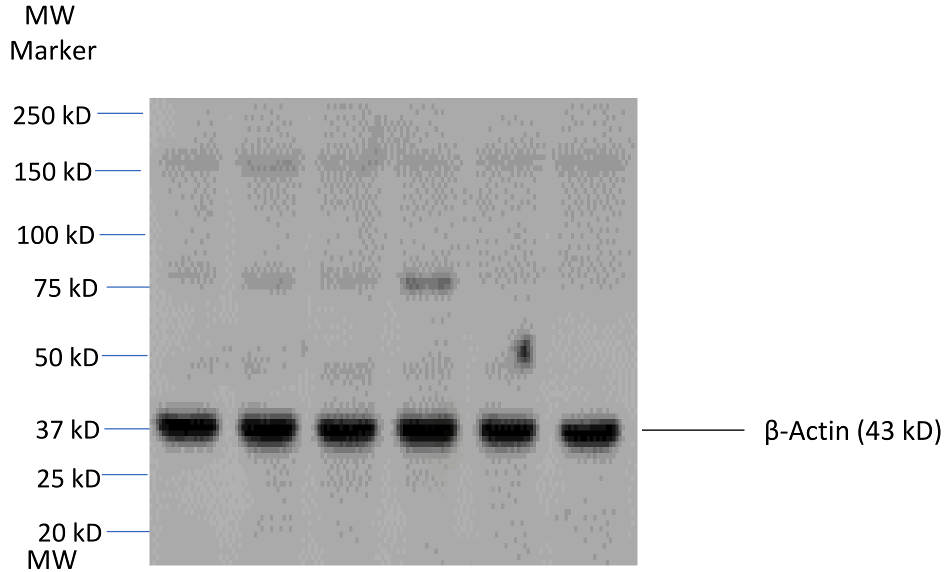

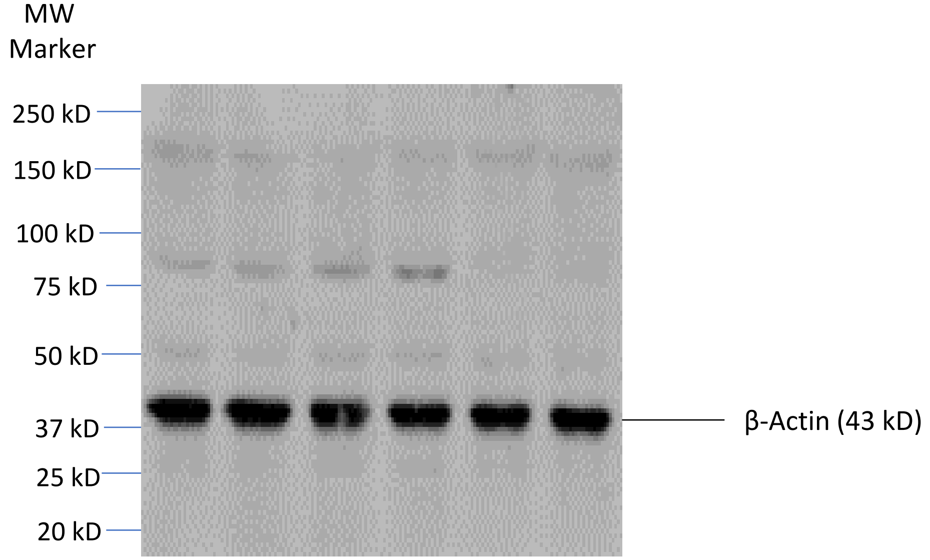

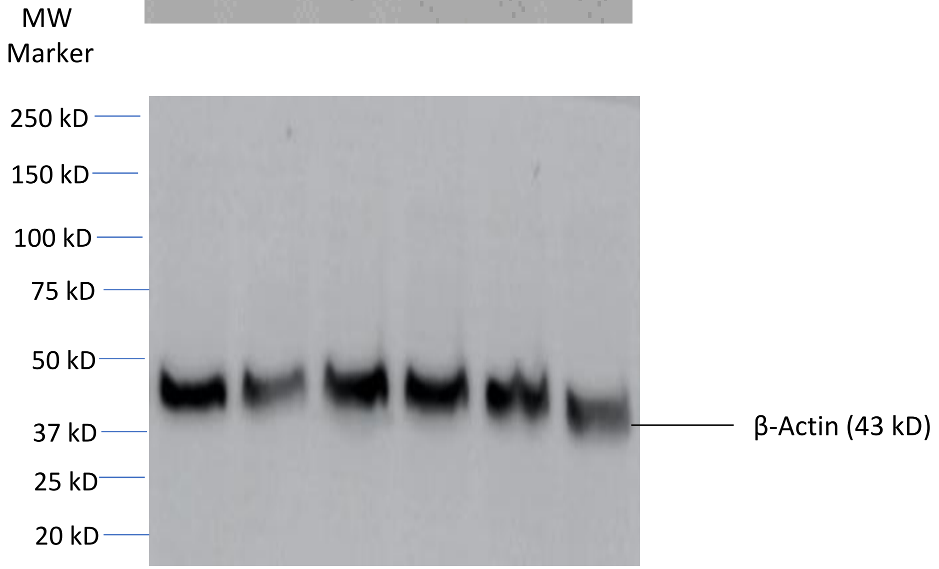

Figure S3D

Cytosolic Nrf2

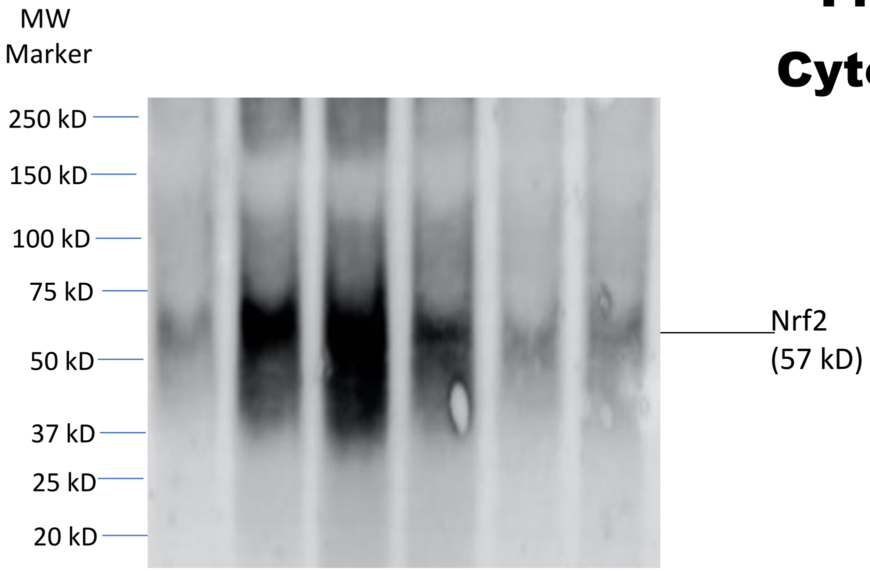

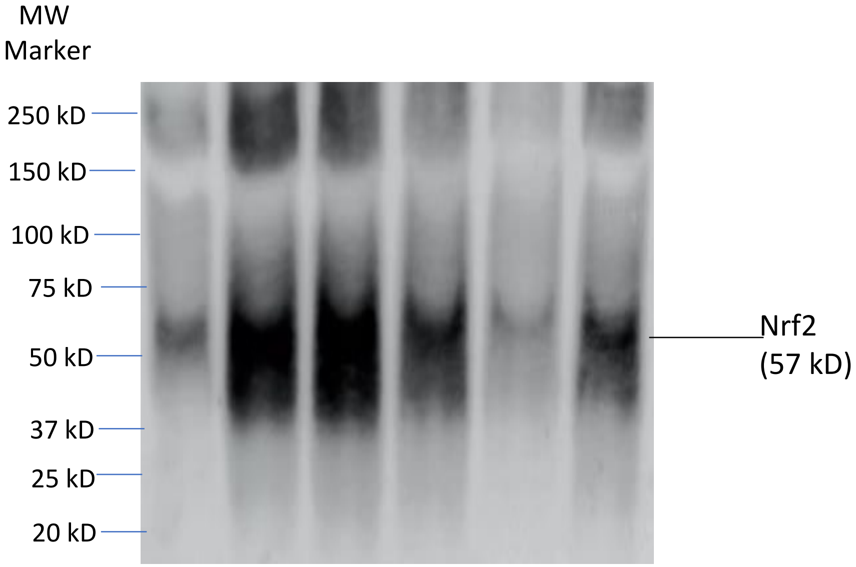

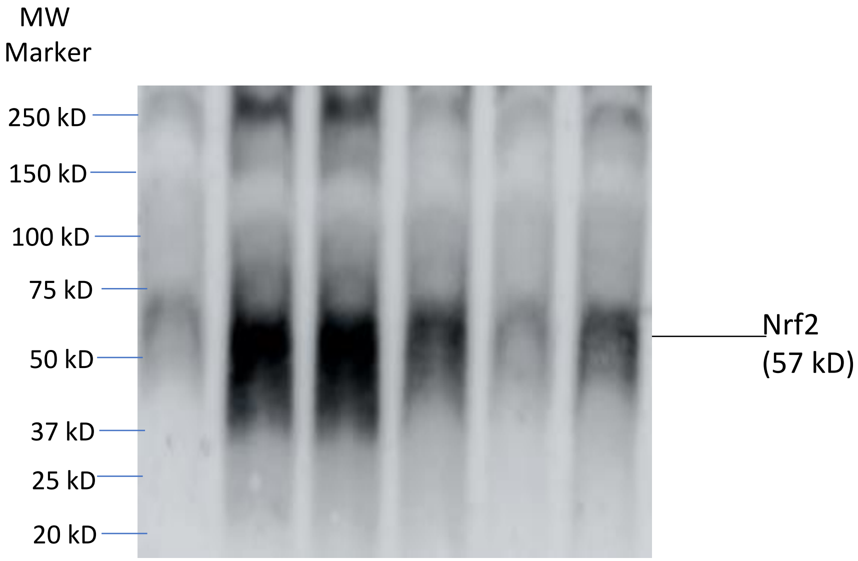

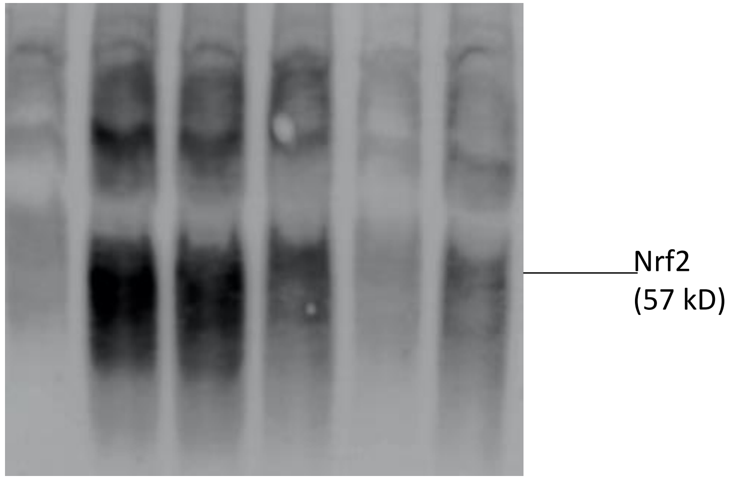

Figure S3D

β-Actin for Cytosolic Nrf2

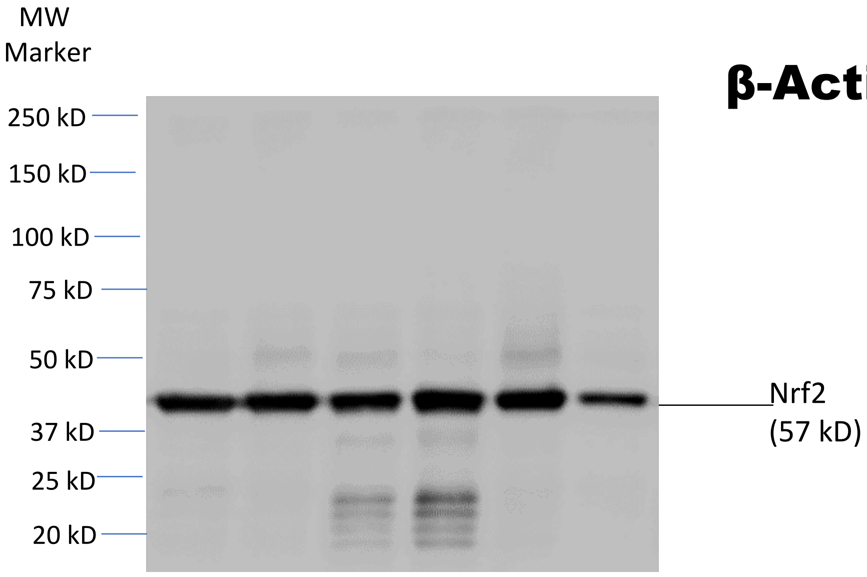

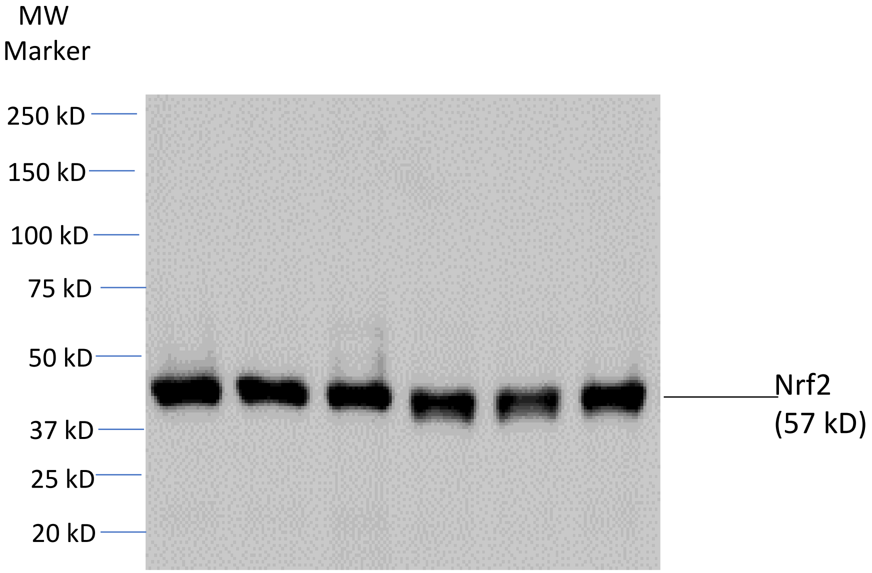

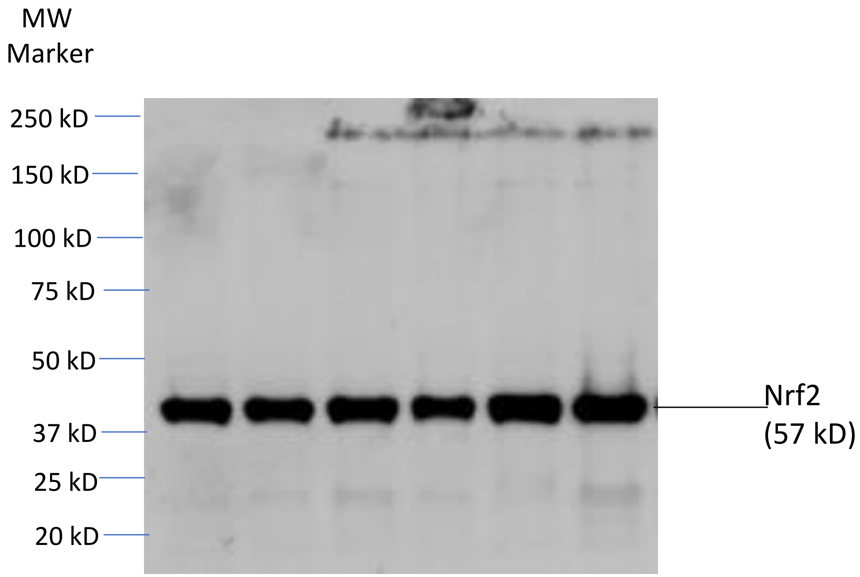

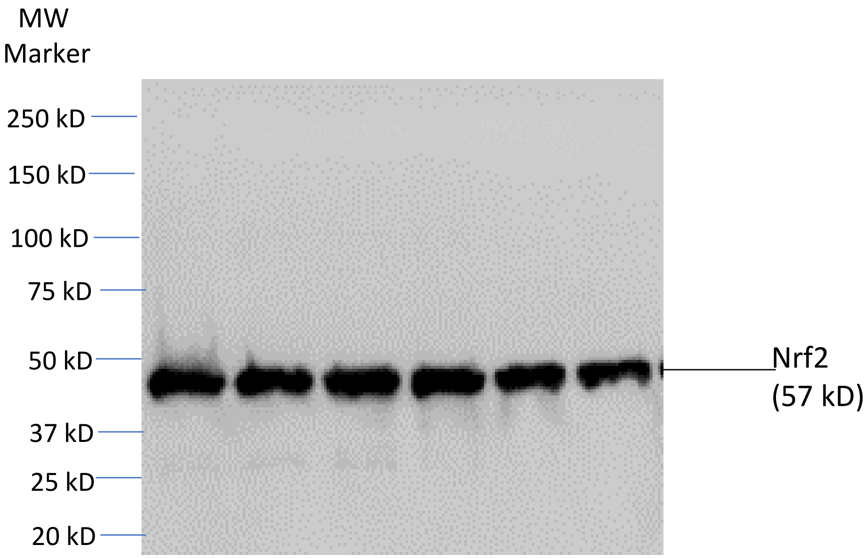

Figure S3E

Nuclear Nrf2

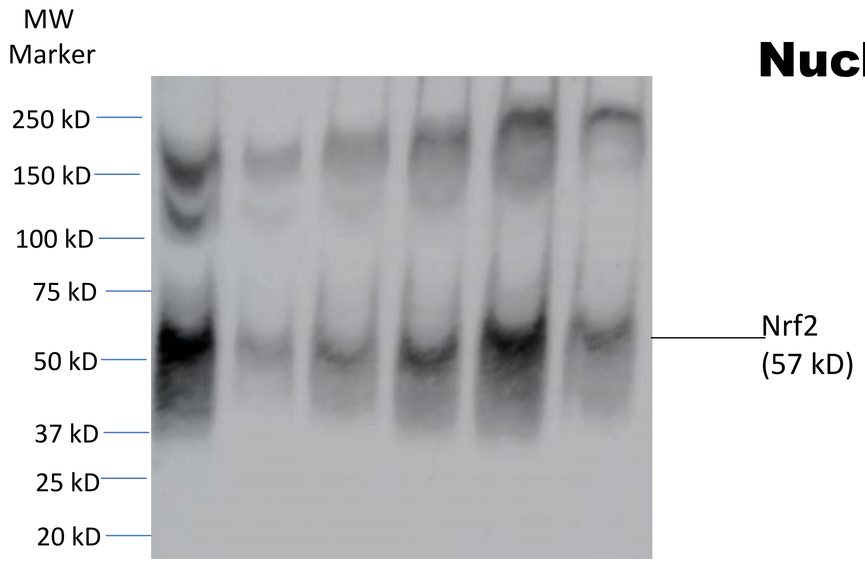

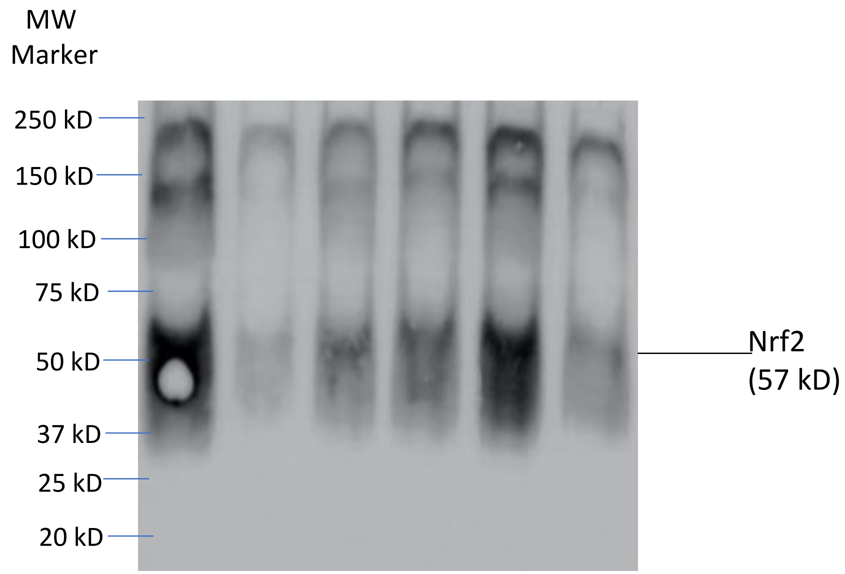

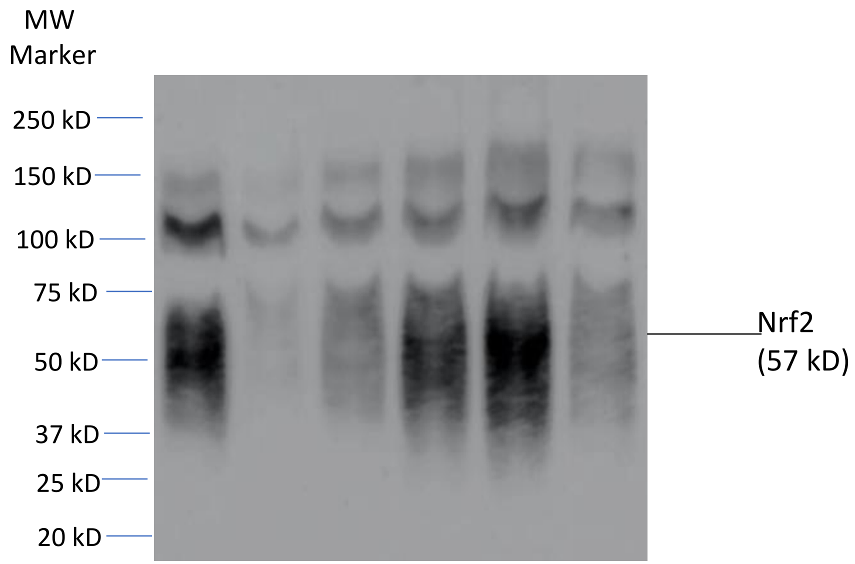

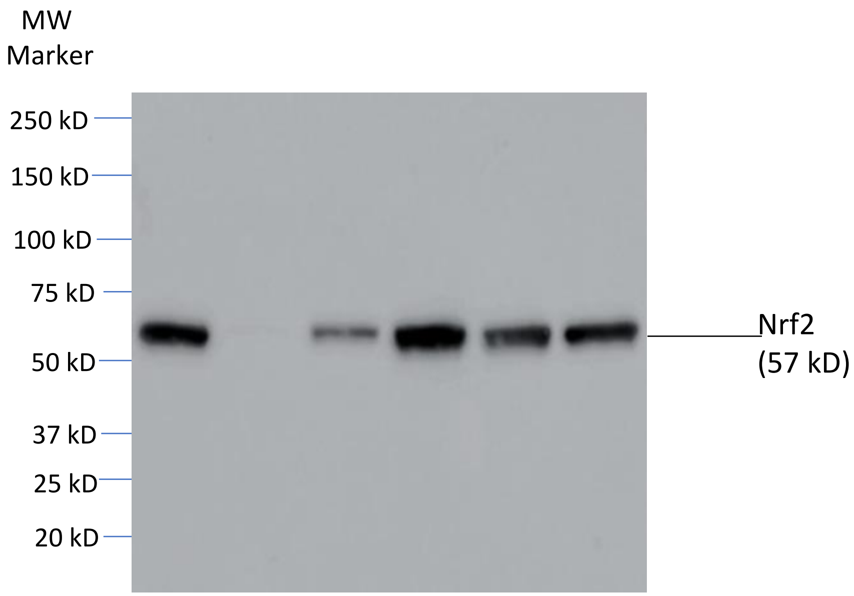

Figure S3E
Lamin B1 for Nuclear Nrf2

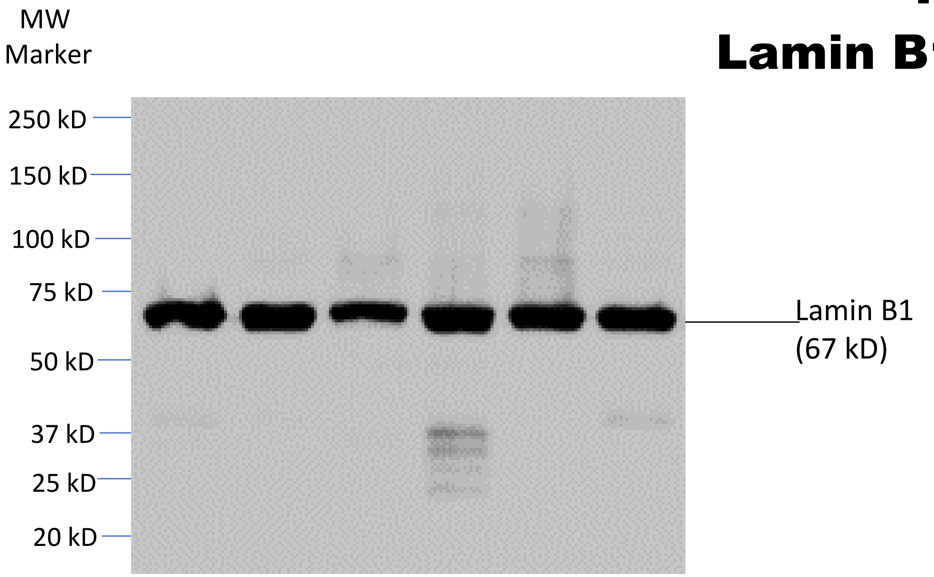

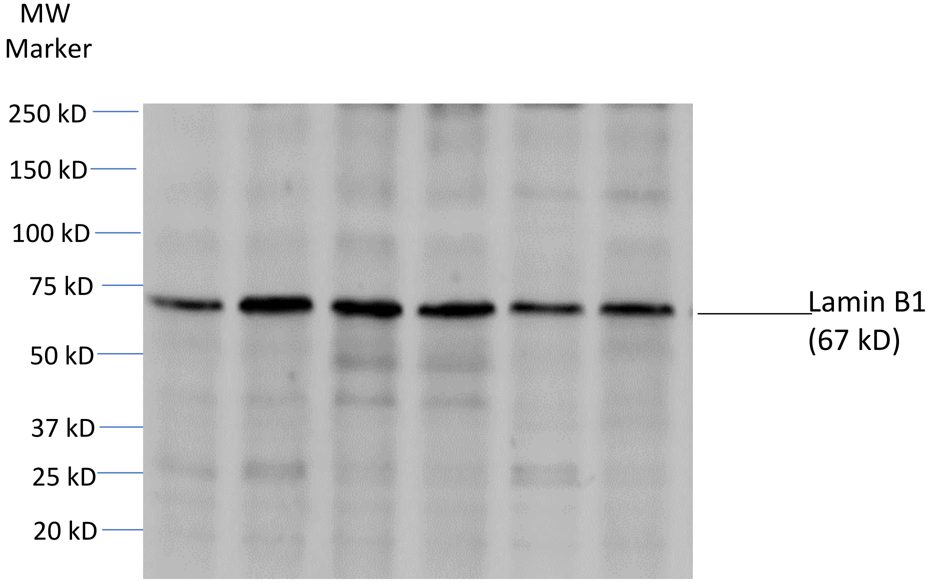

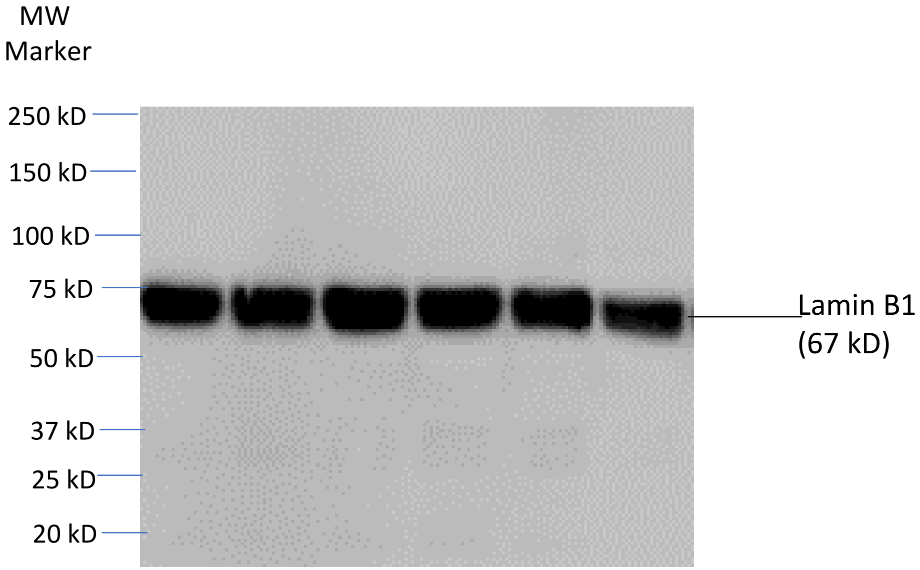

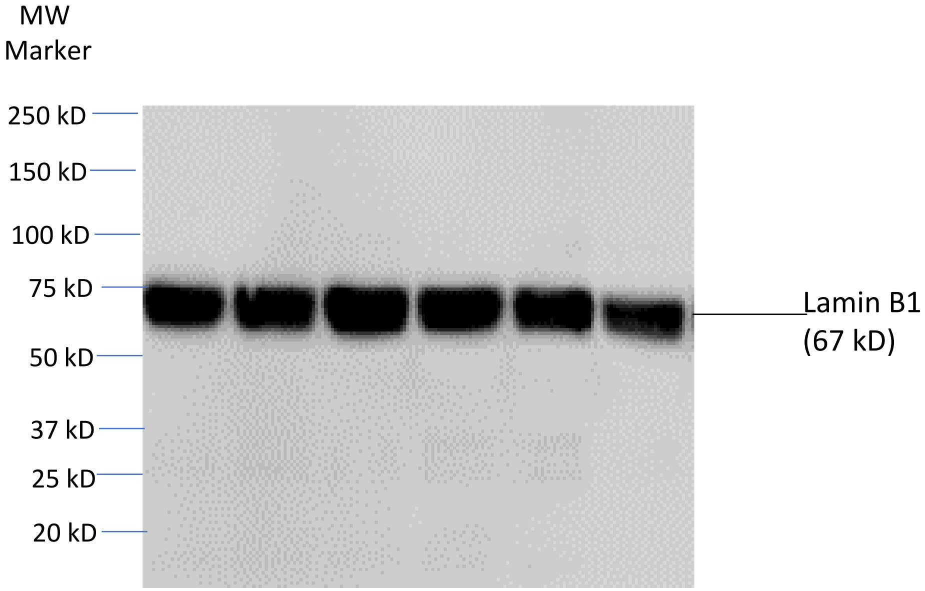

Figure S3E

GCLM

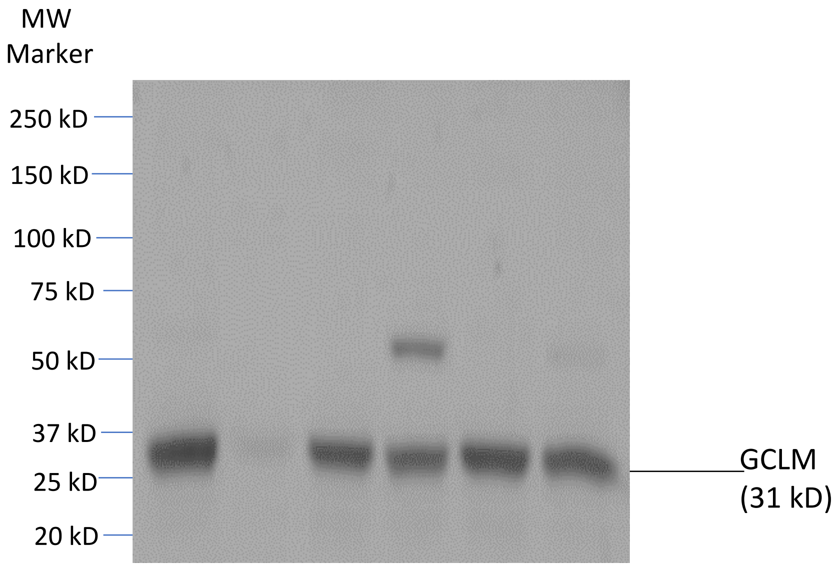

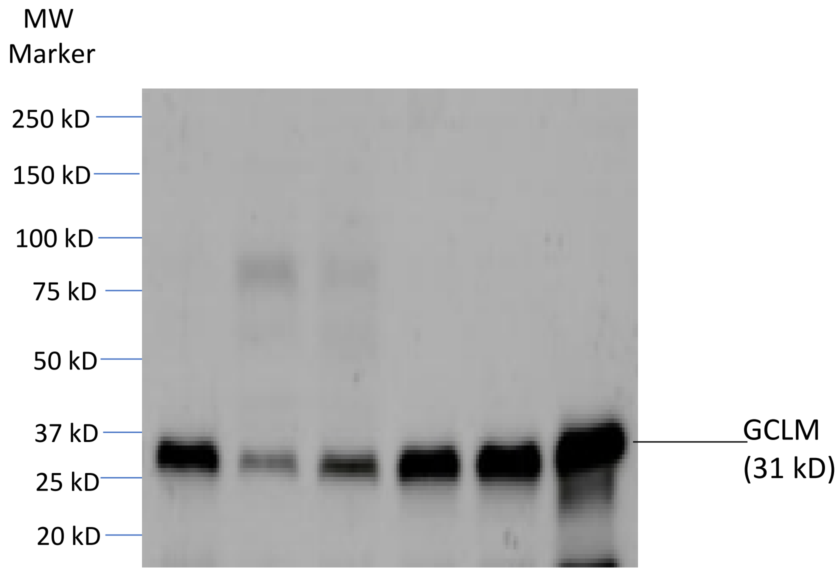

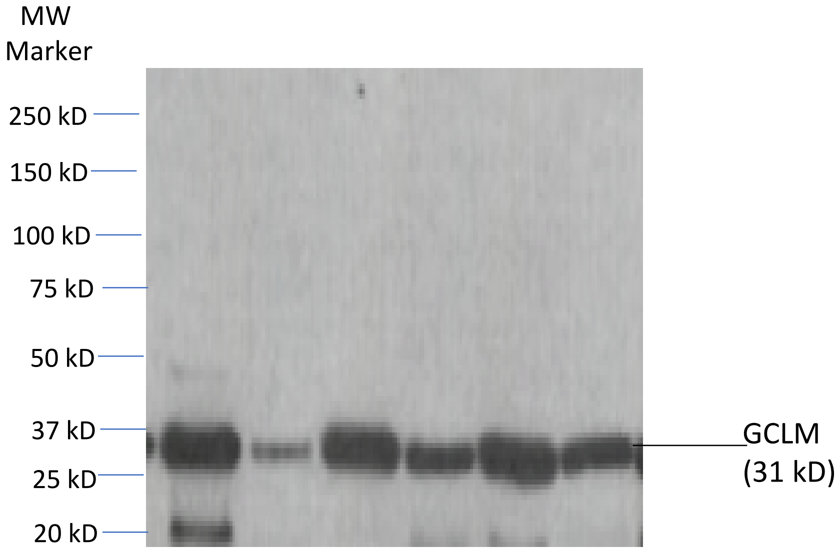

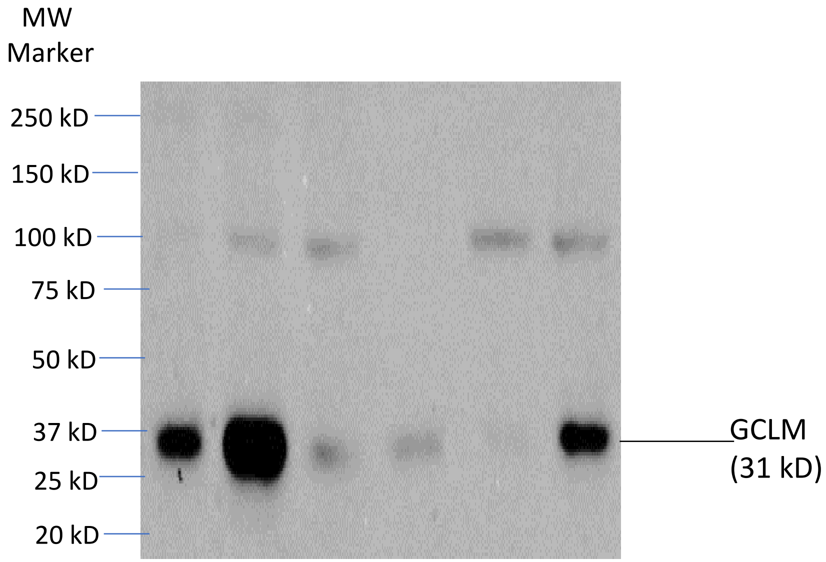

Figure S3E

β-Actin for GCLM

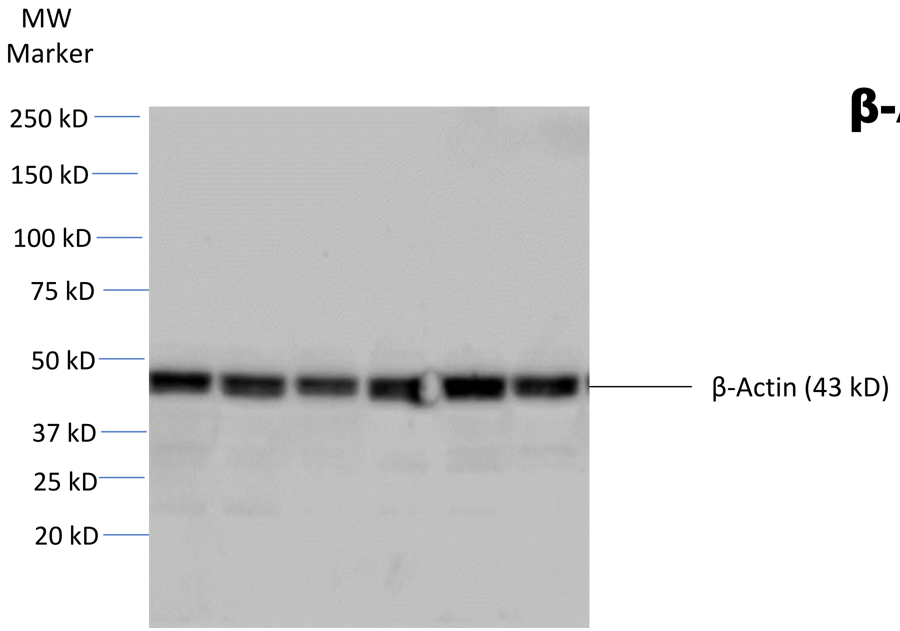

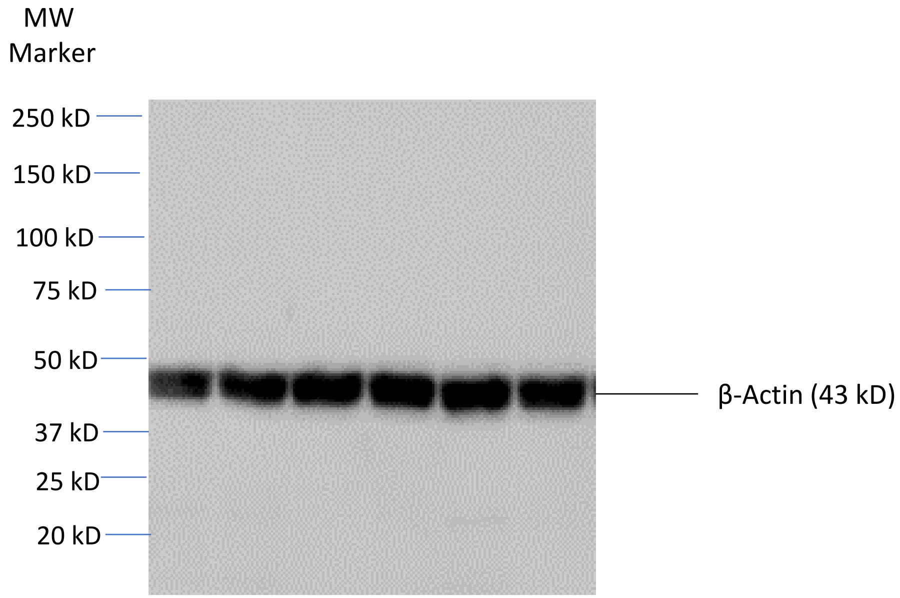

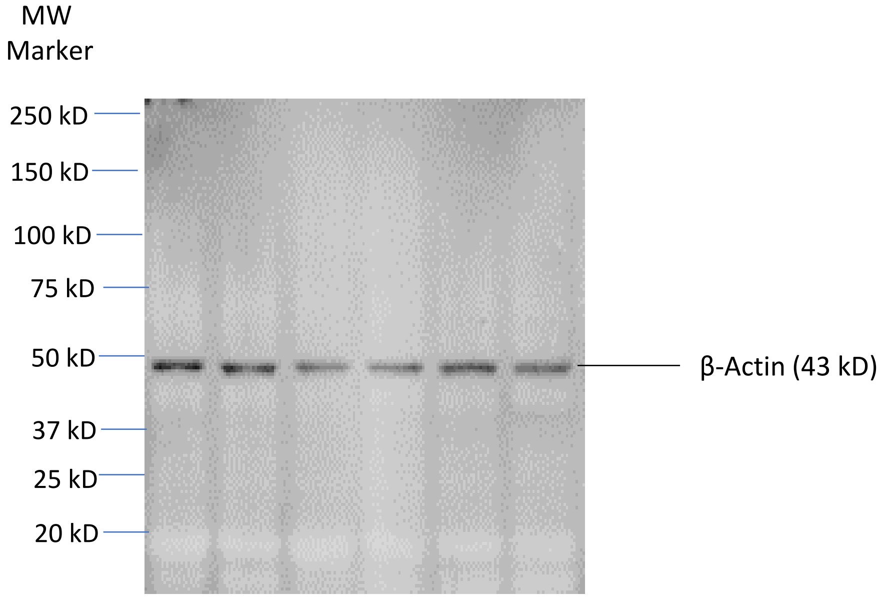

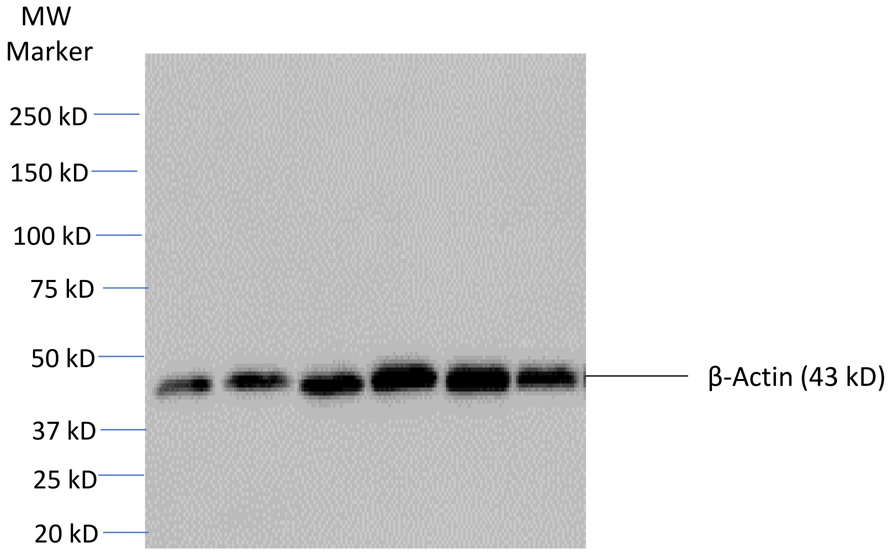

Figure S3J

GCLC

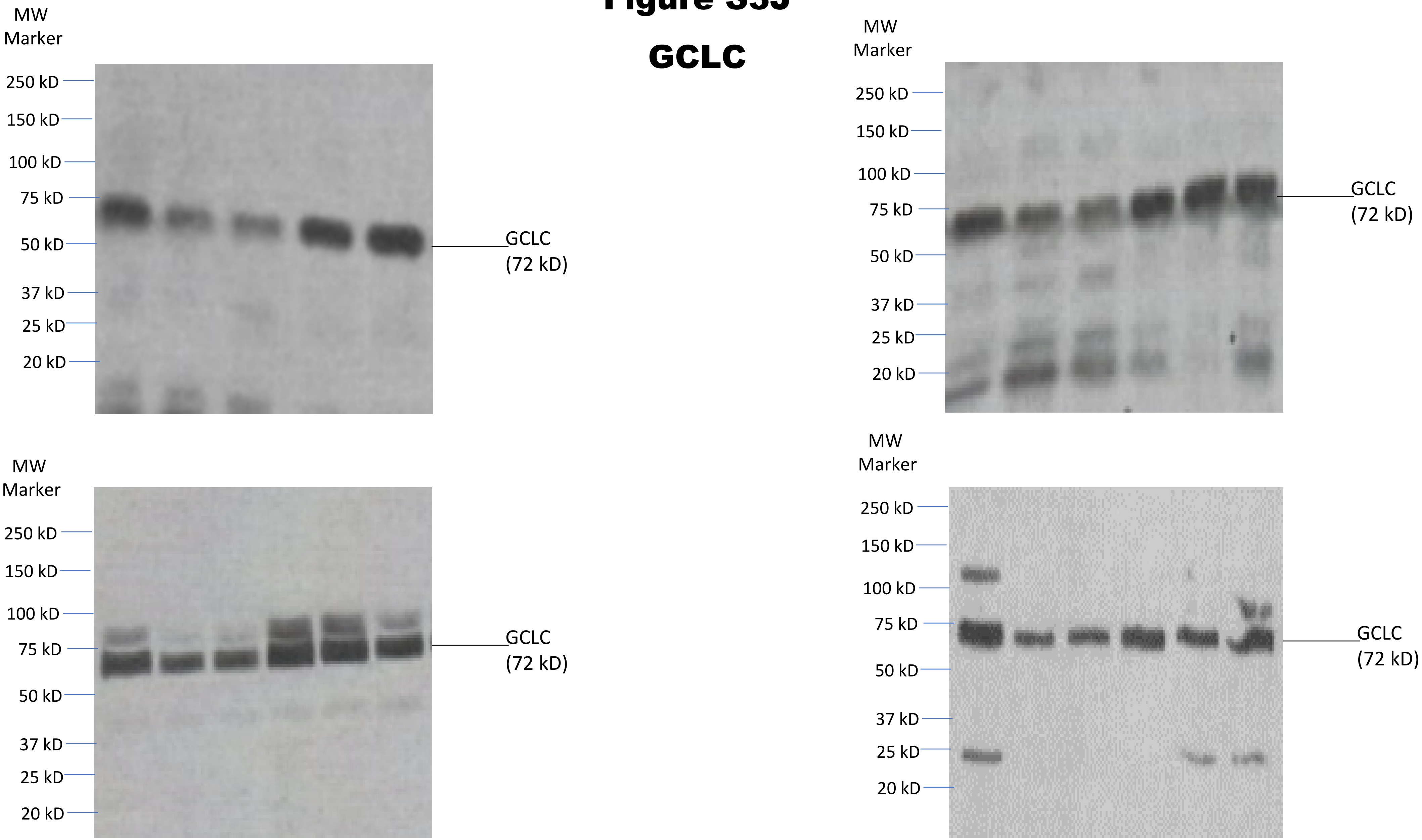

Figure S3J
β-Actin for GCLC

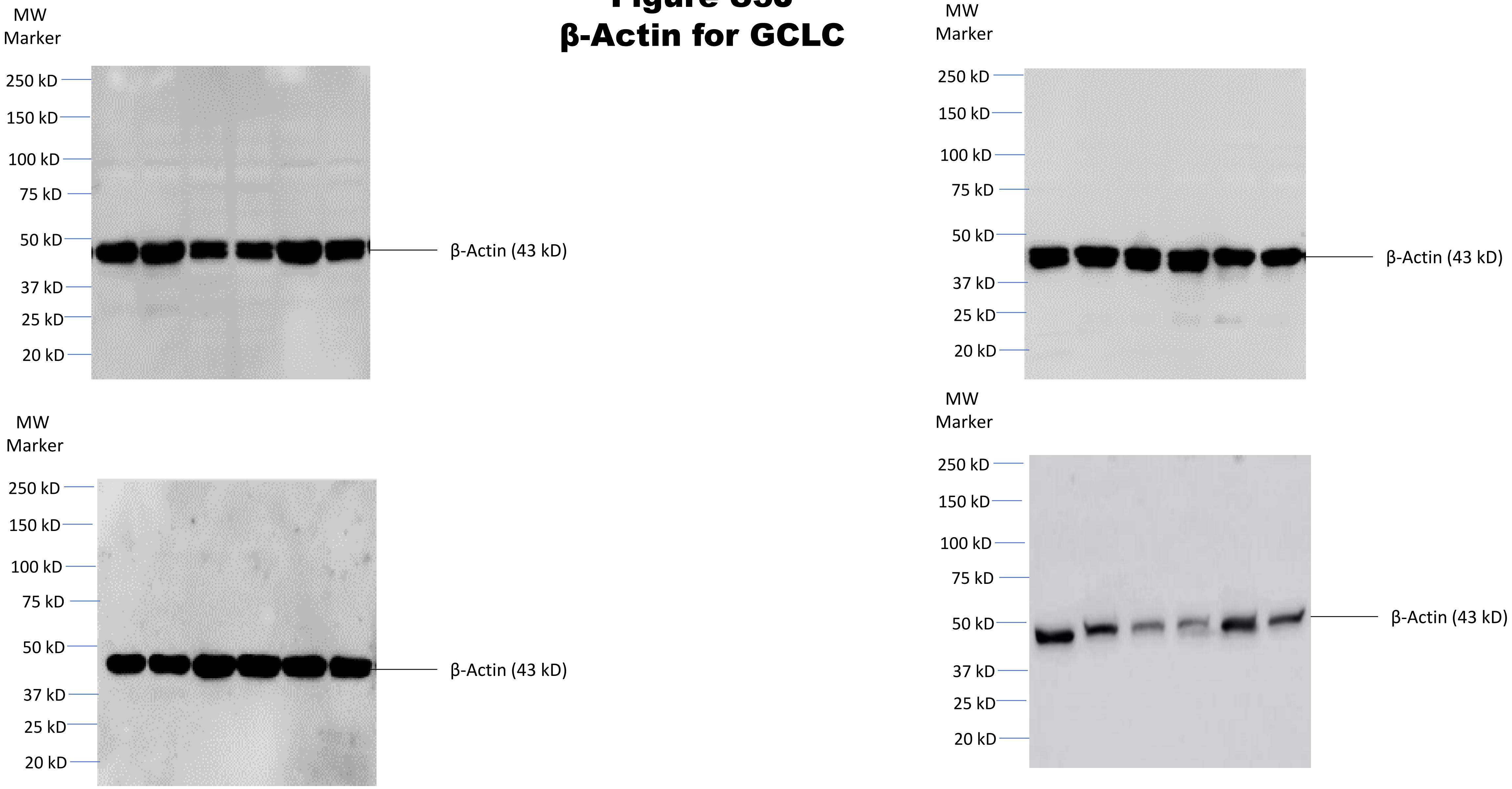

Figure S3K

Nqo1

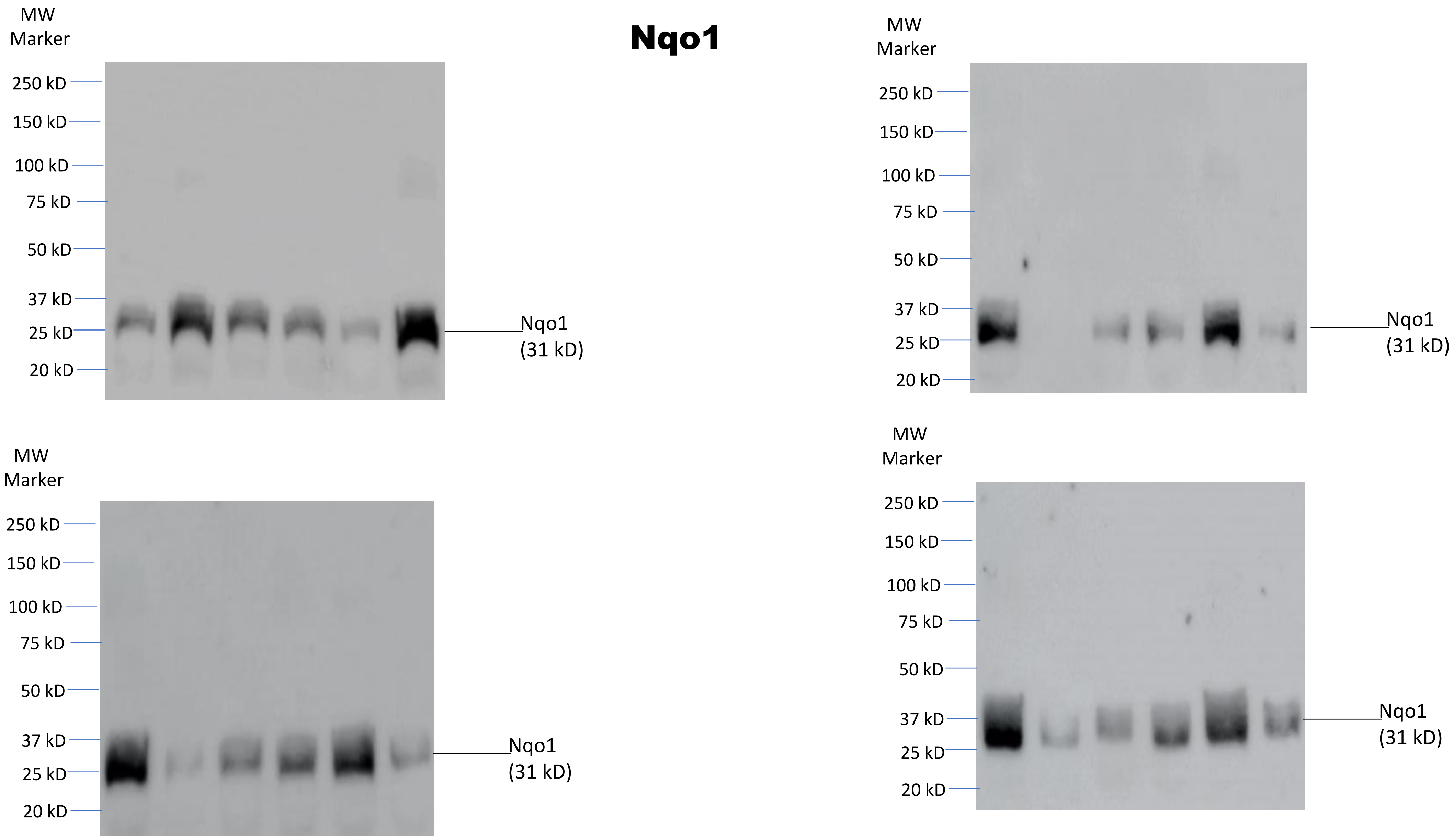

Figure S3K

β-Actin for Nqo1

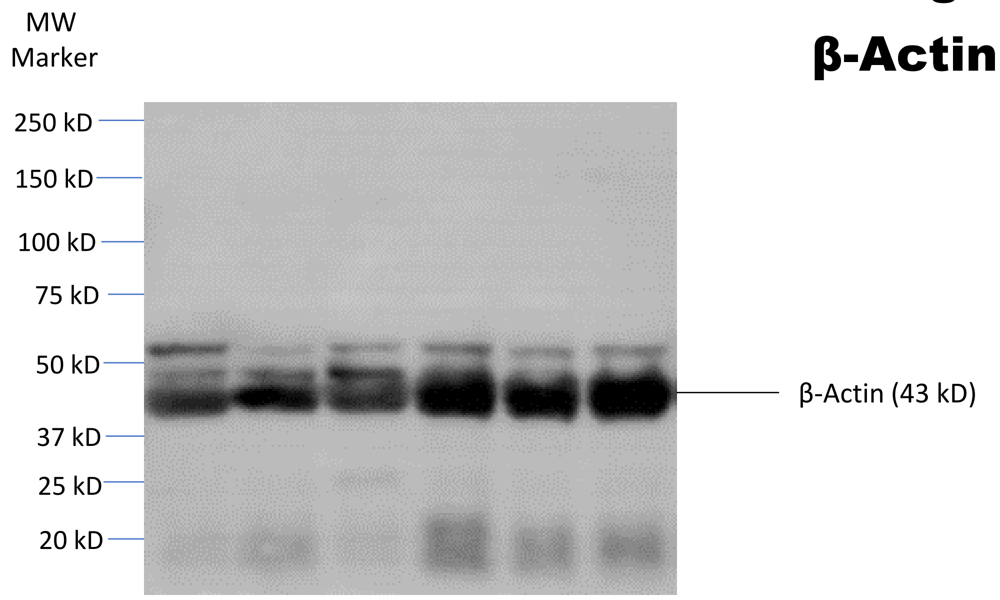

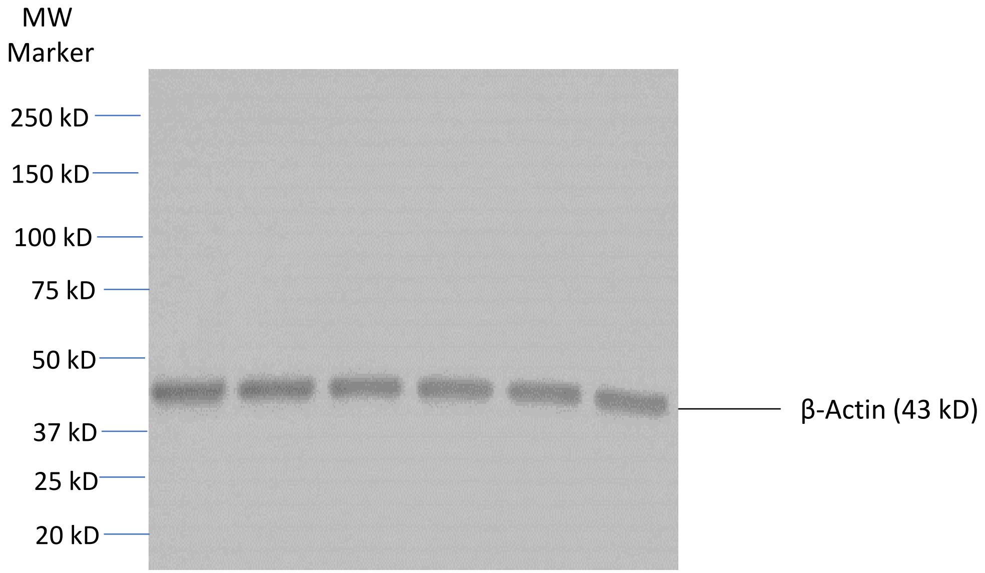

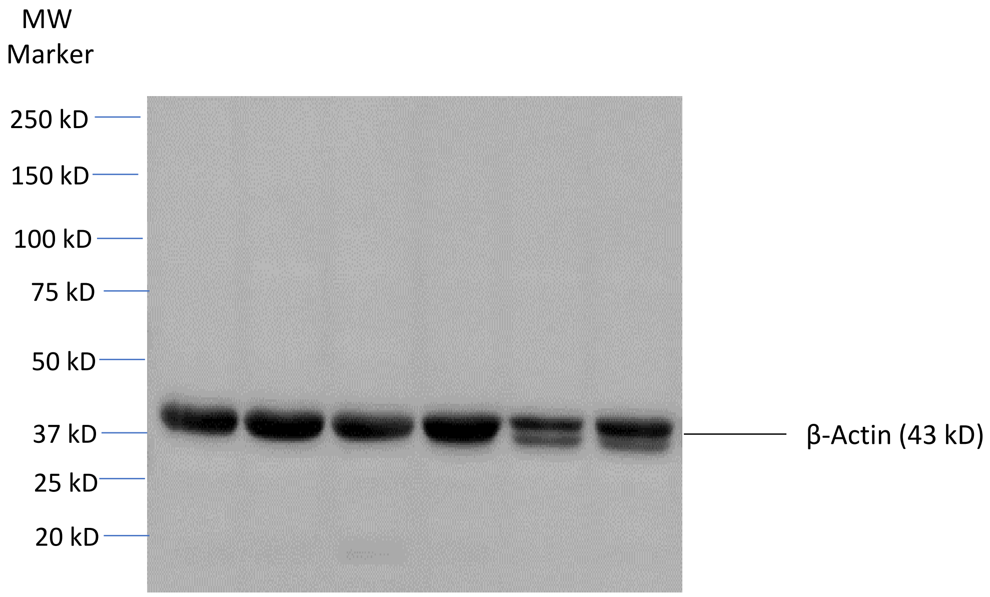

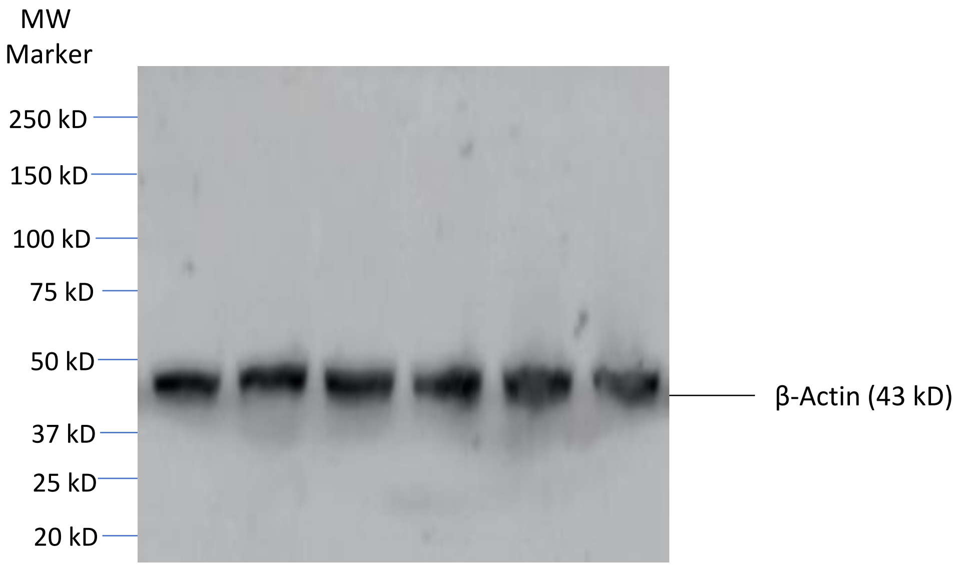

Figure S4C
nNOS α

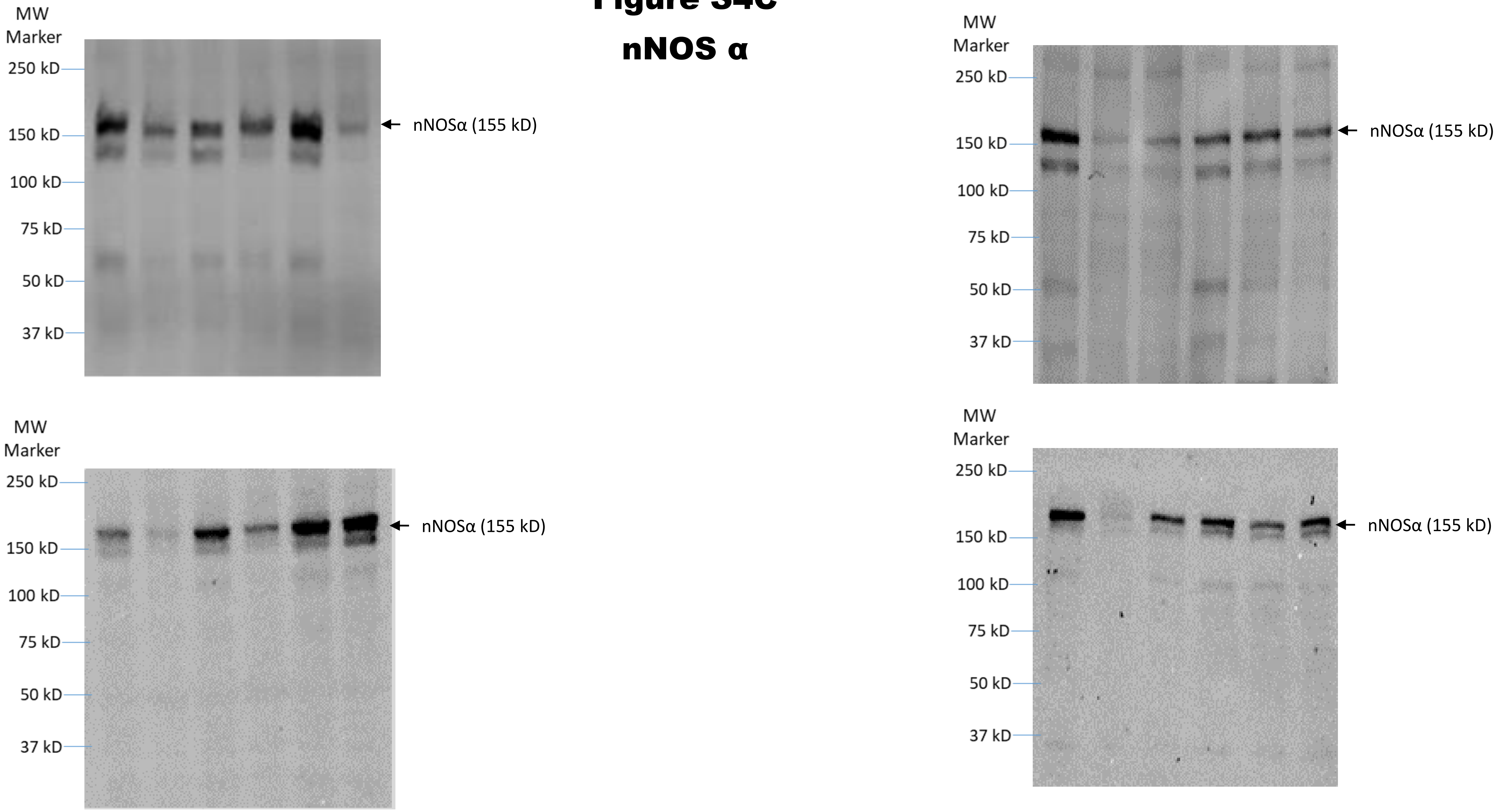

Figure S4C

β-Actin for nNOS α

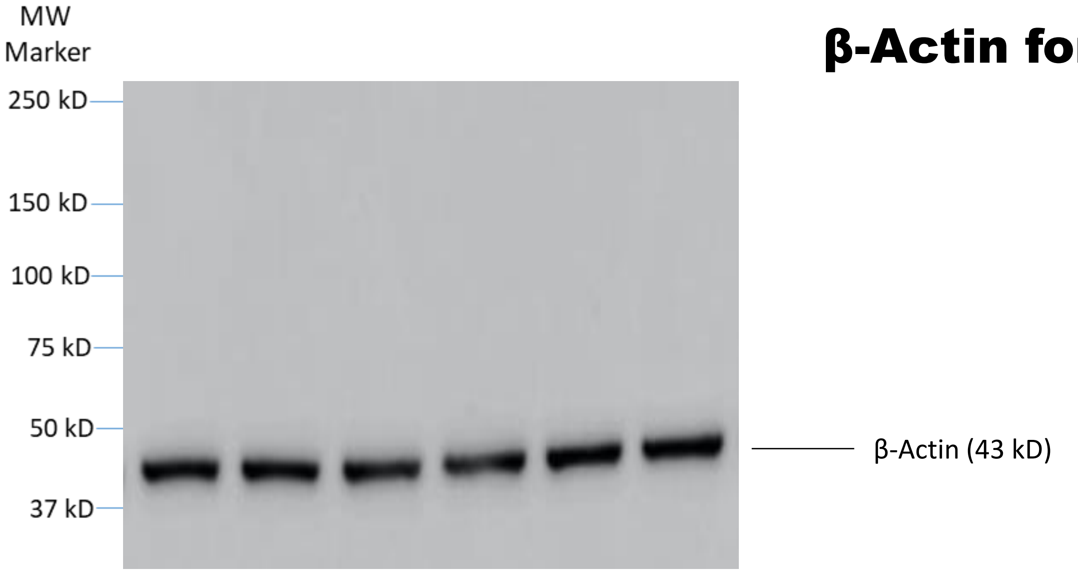

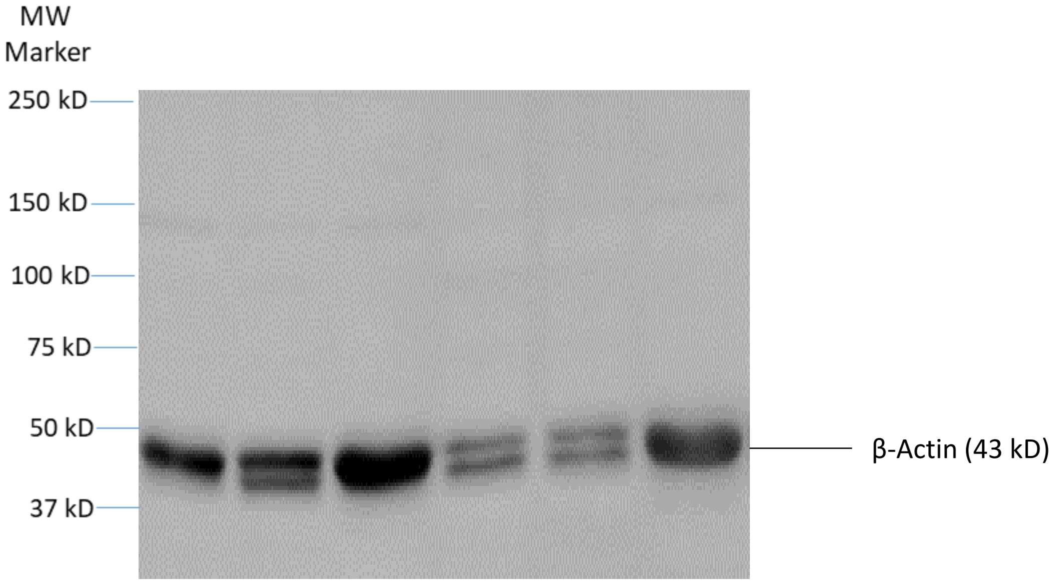

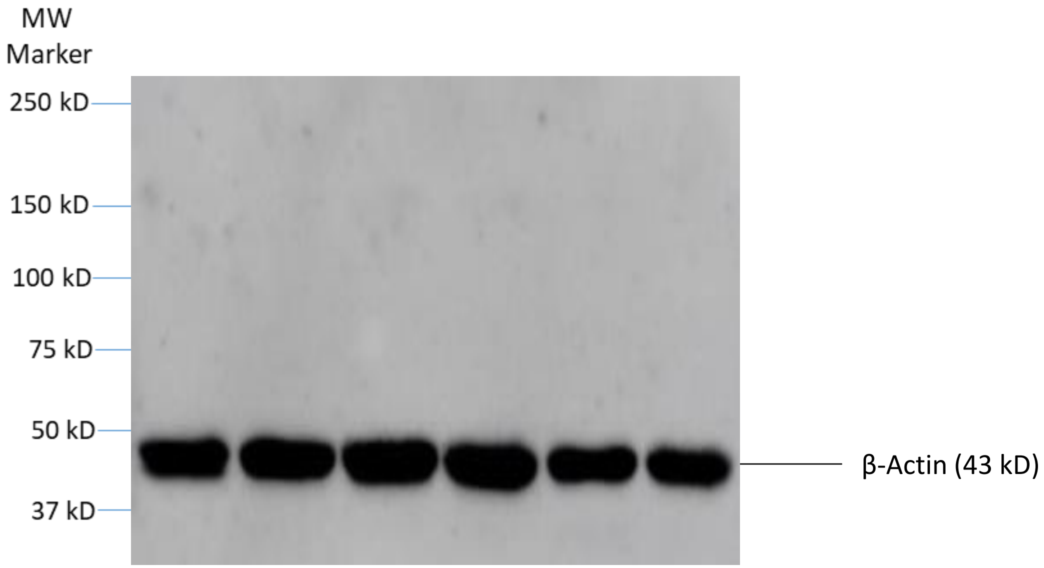

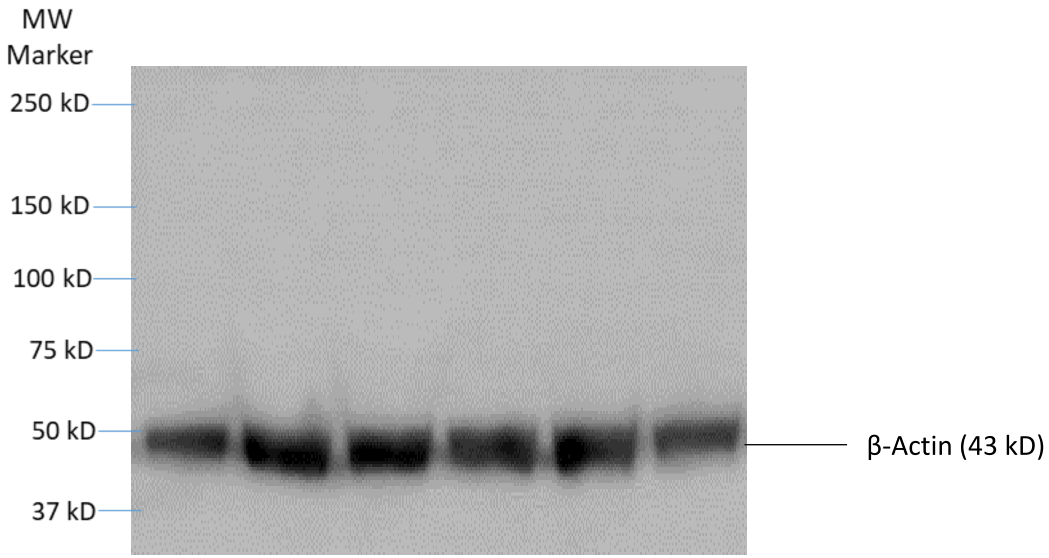

Figure S4D
GCH-1

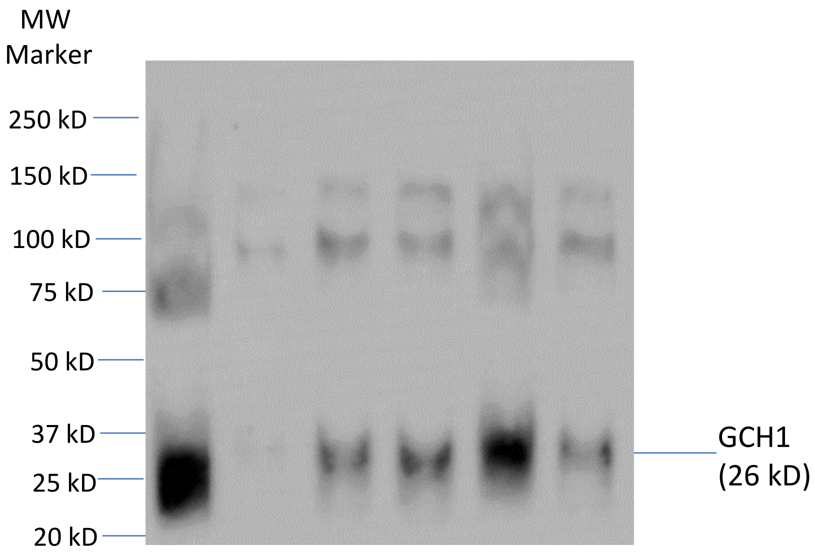

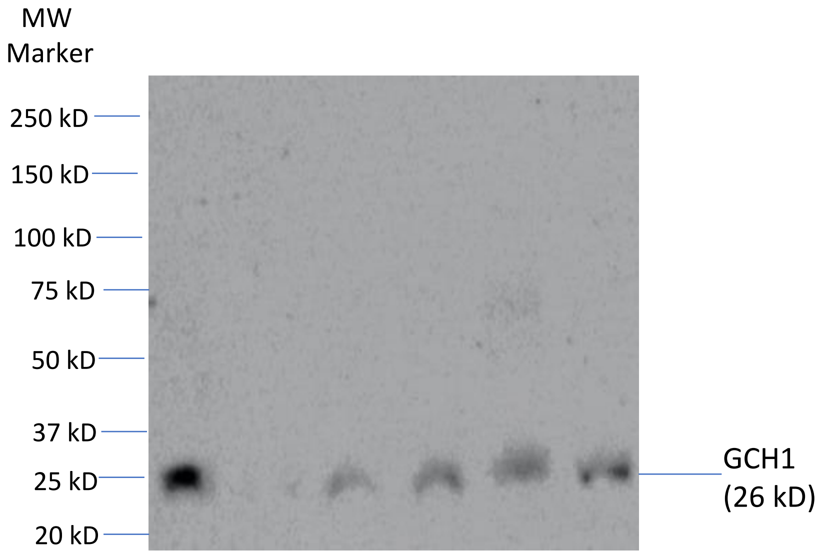

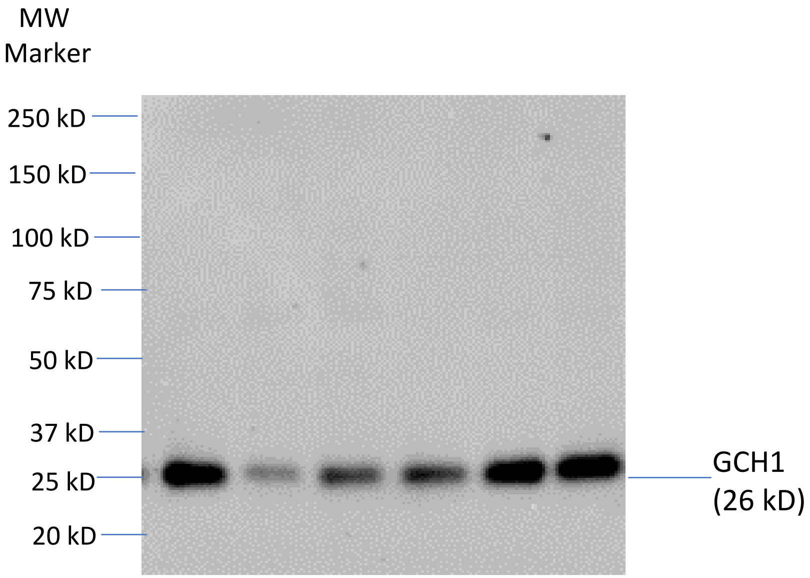

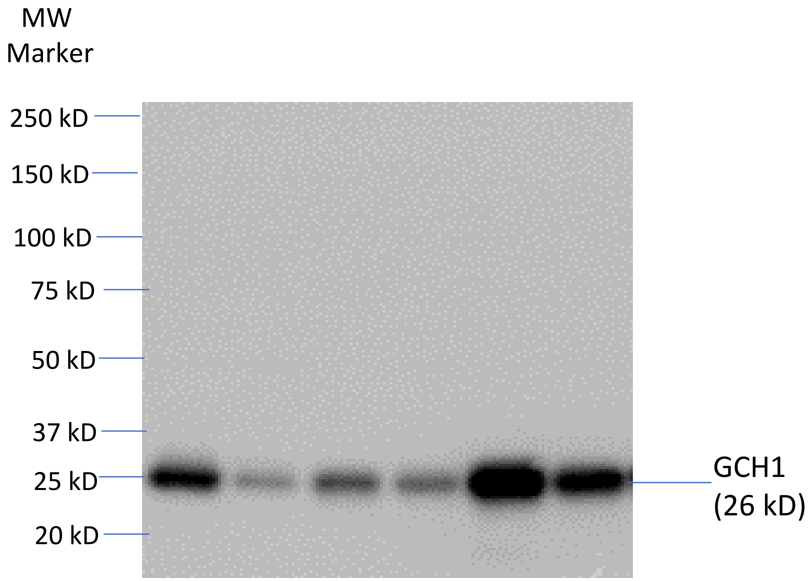

Figure S4D
β-Actin for GCH-1

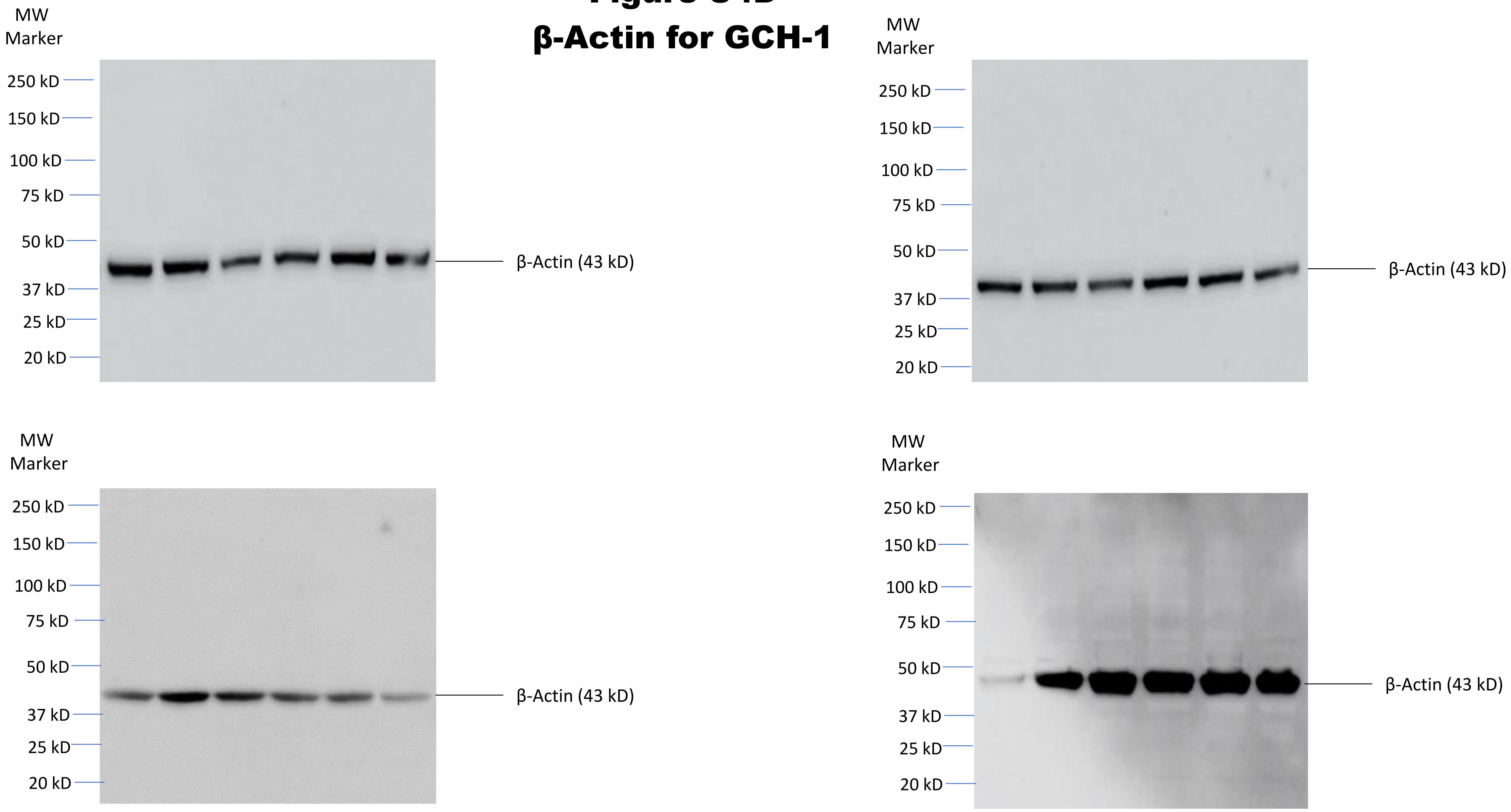

Figure S5A
Ikk β

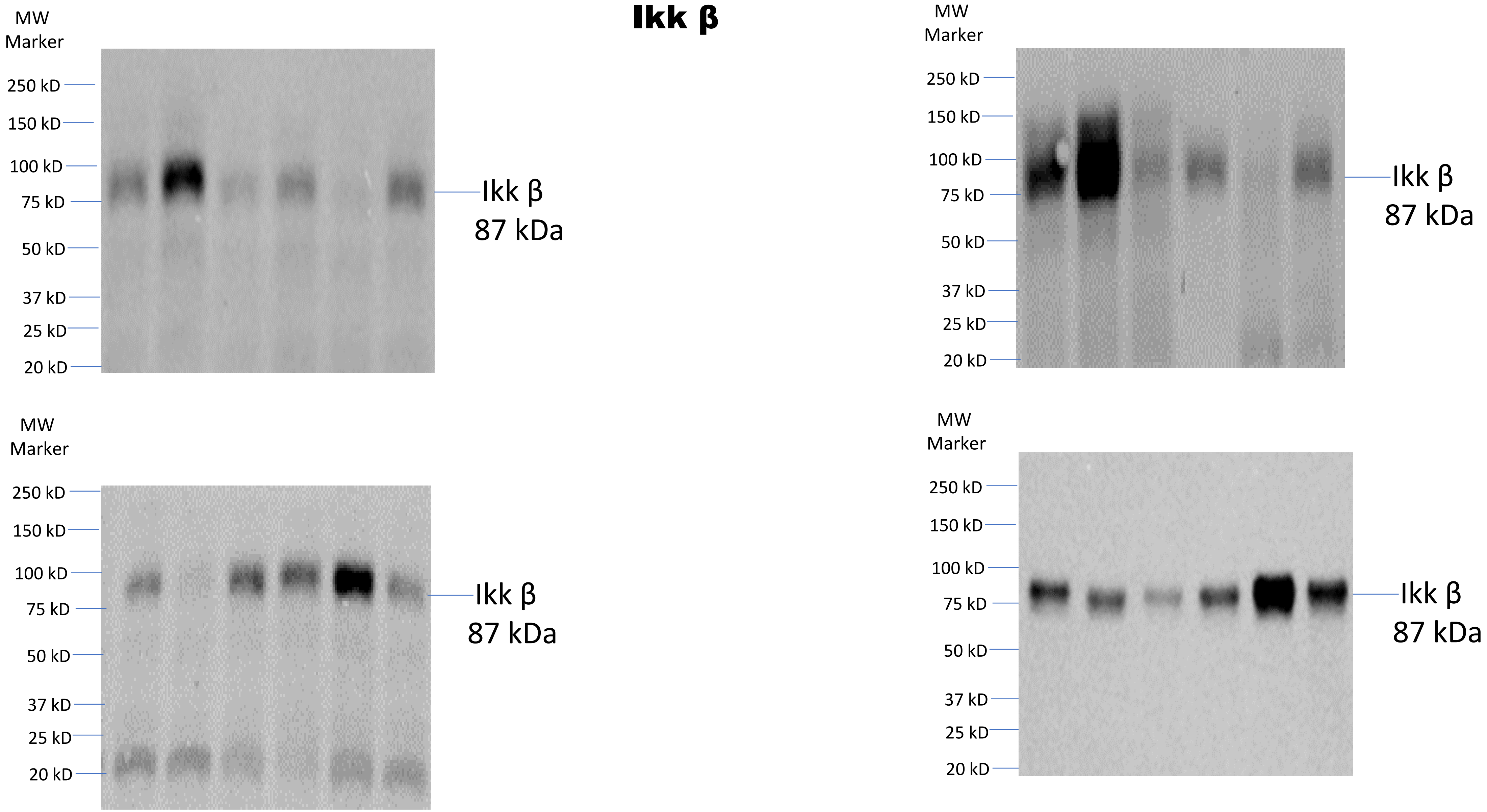

Figure S5A

β-Actin for Ikk β

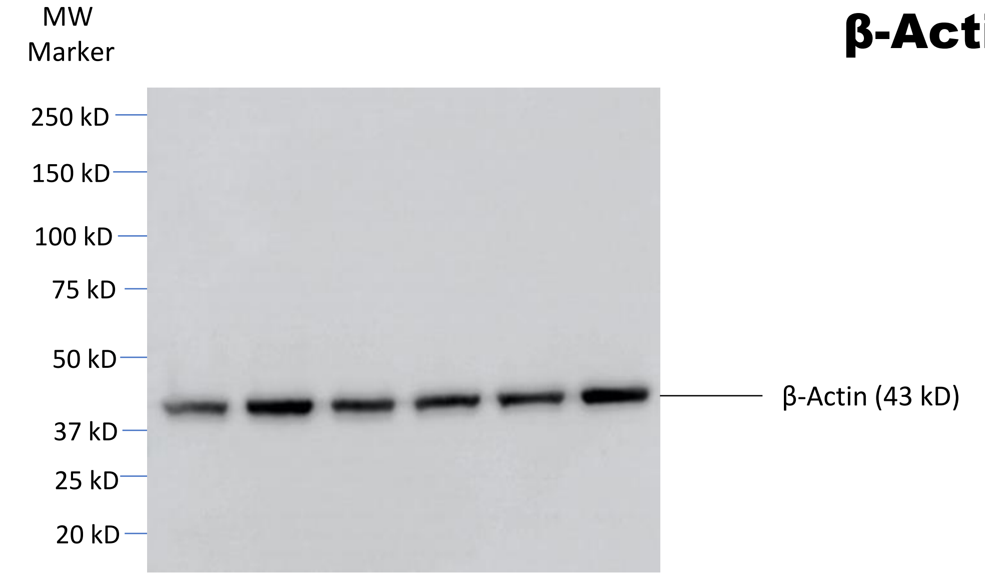

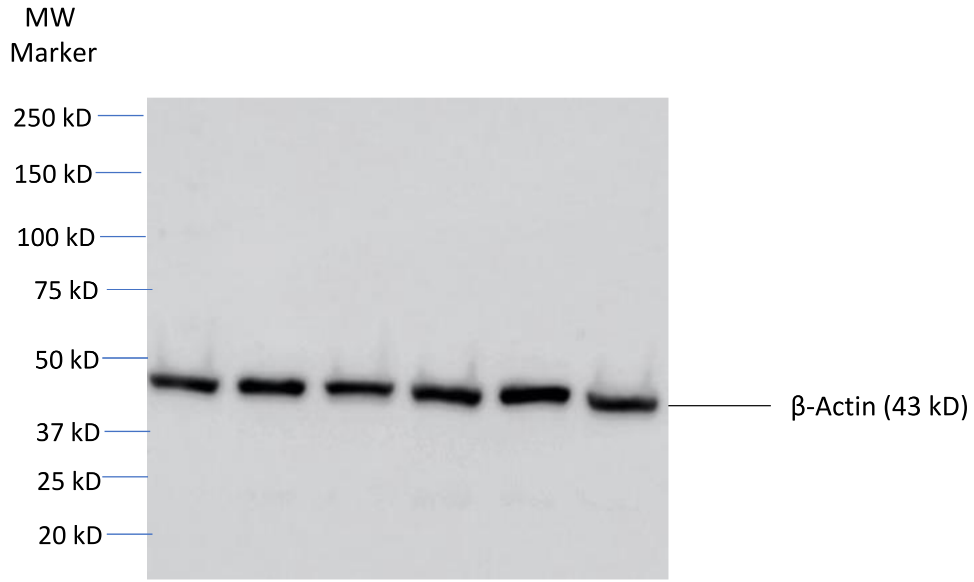

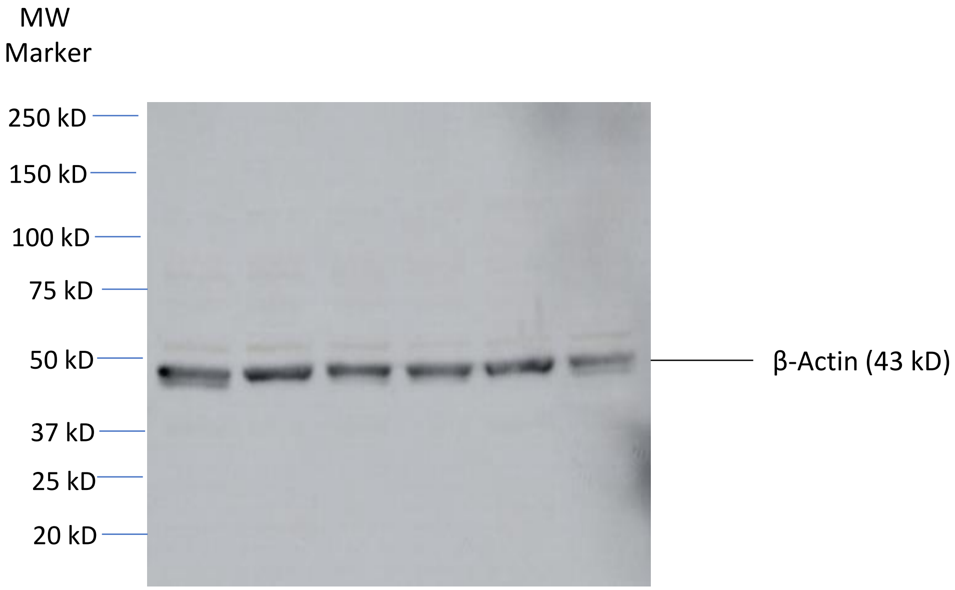

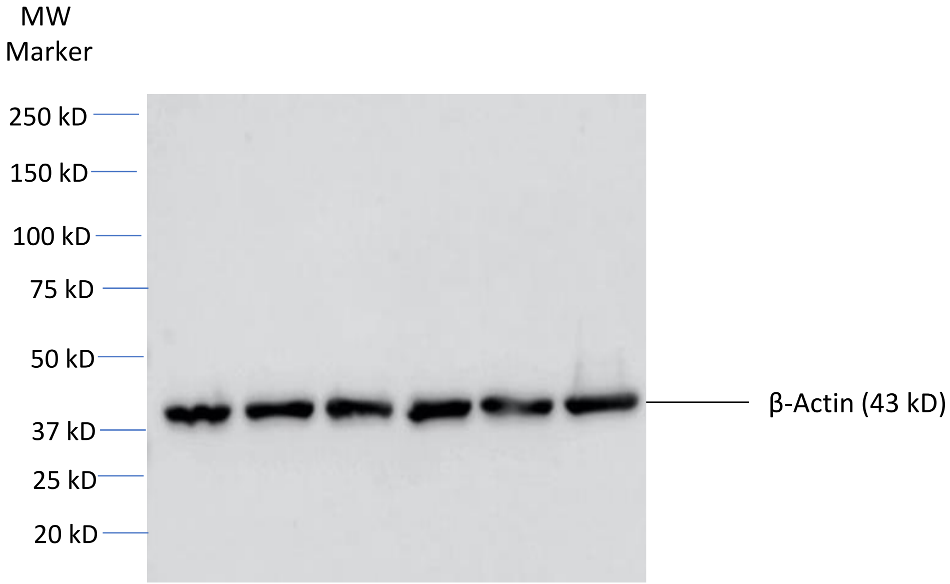

Figure S5B

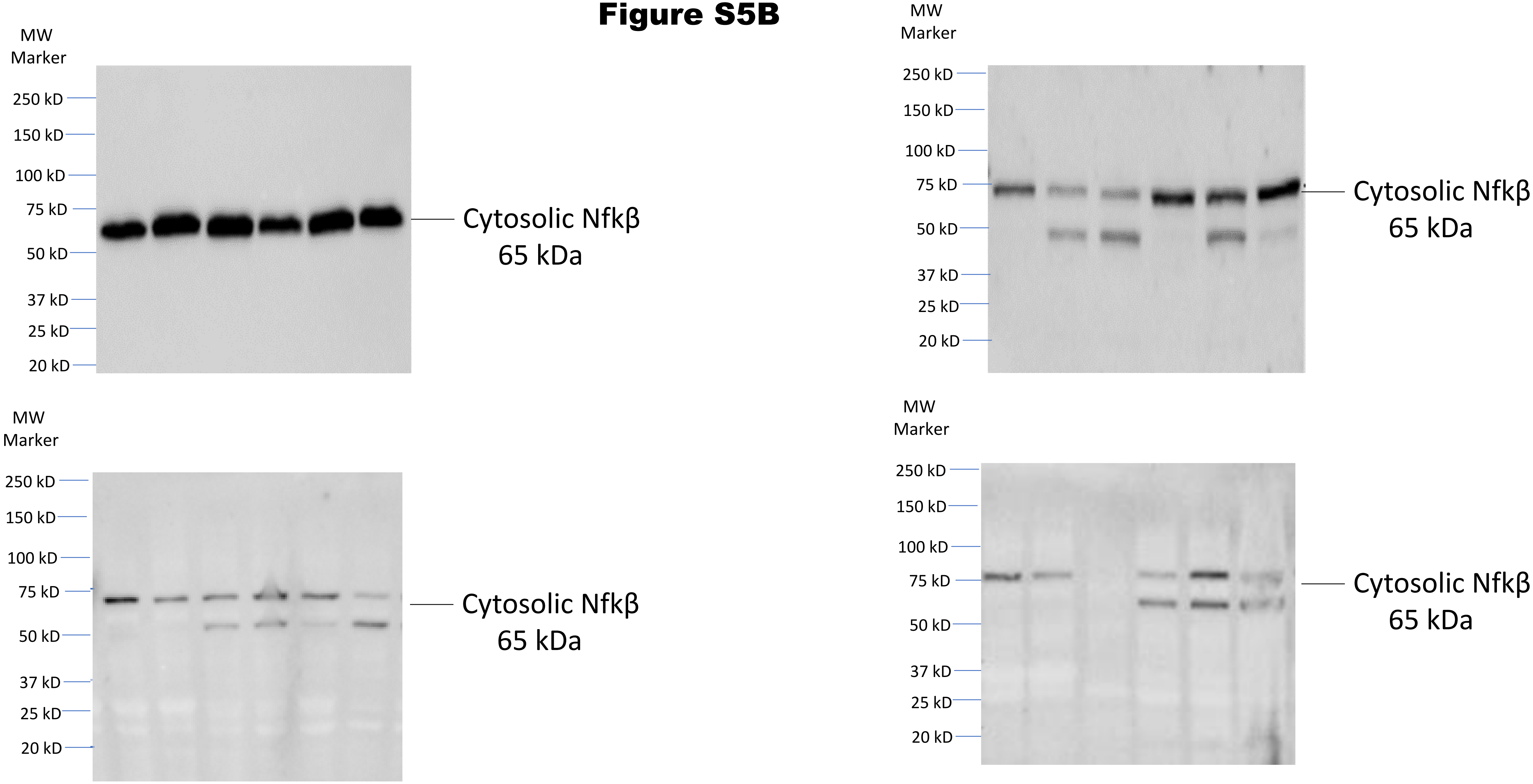
MW Marker
250 kD
150 kD
100 kD
75 kD
50 kD
37 kD
25 kD
20 kD
Cytosolic Nfkβ
65 kDa
MW Marker
250 kD
150 kD
100 kD
75 kD
50 kD
37 kD
25 kD
20 kD
Cytosolic Nfkβ
65 kDa
MW Marker
250 kD
150 kD
100 kD
75 kD
50 kD
37 kD
25 kD
20 kD
Cytosolic Nfkβ
65 kDa
MW Marker
250 kD
150 kD
100 kD
75 kD
50 kD
37 kD
25 kD
20 kD
Cytosolic Nfkβ
65 kDa

Figure S5B
β-Actin for Cytosolic NfkB

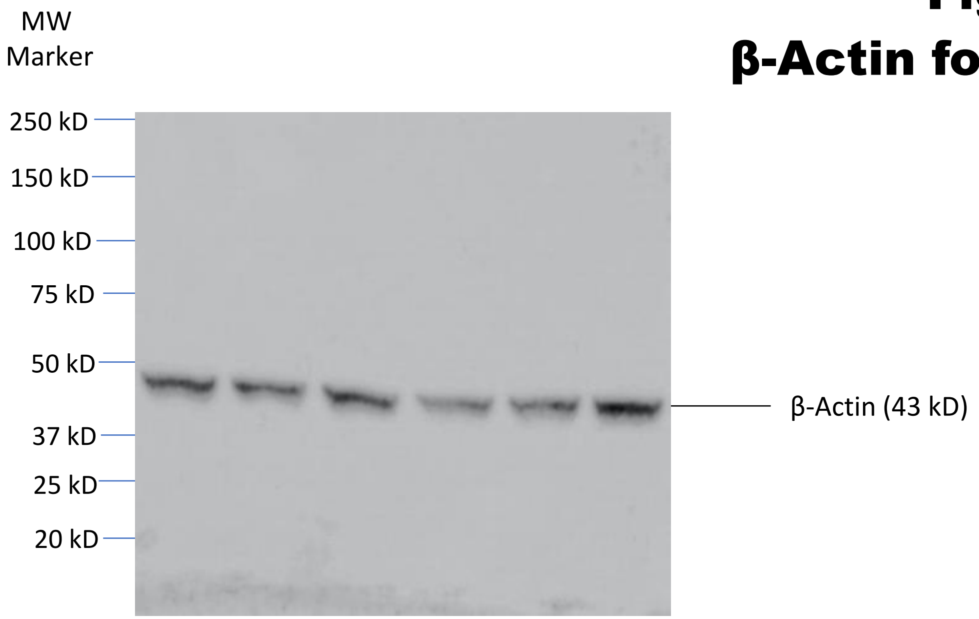

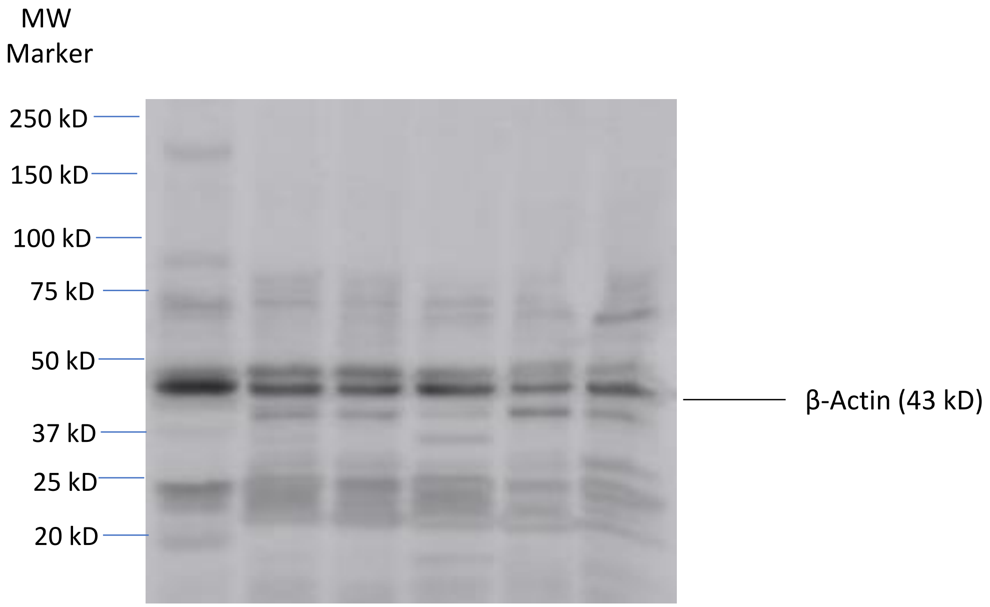

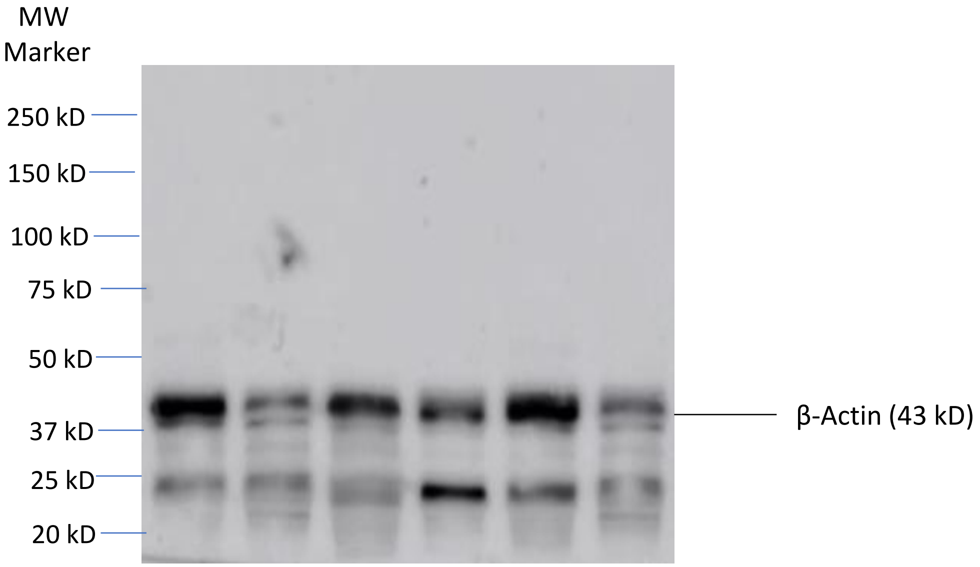

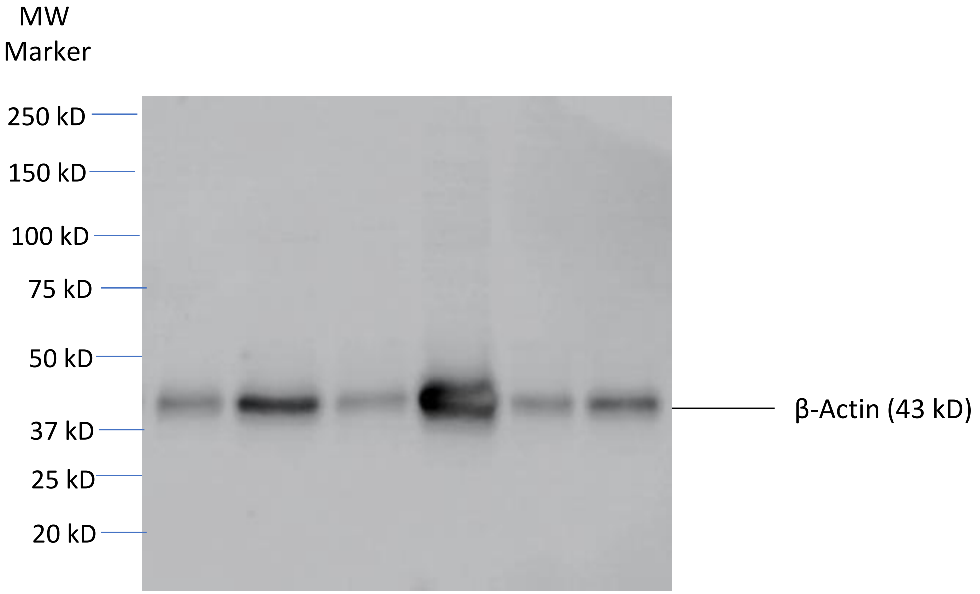

Figure S5B

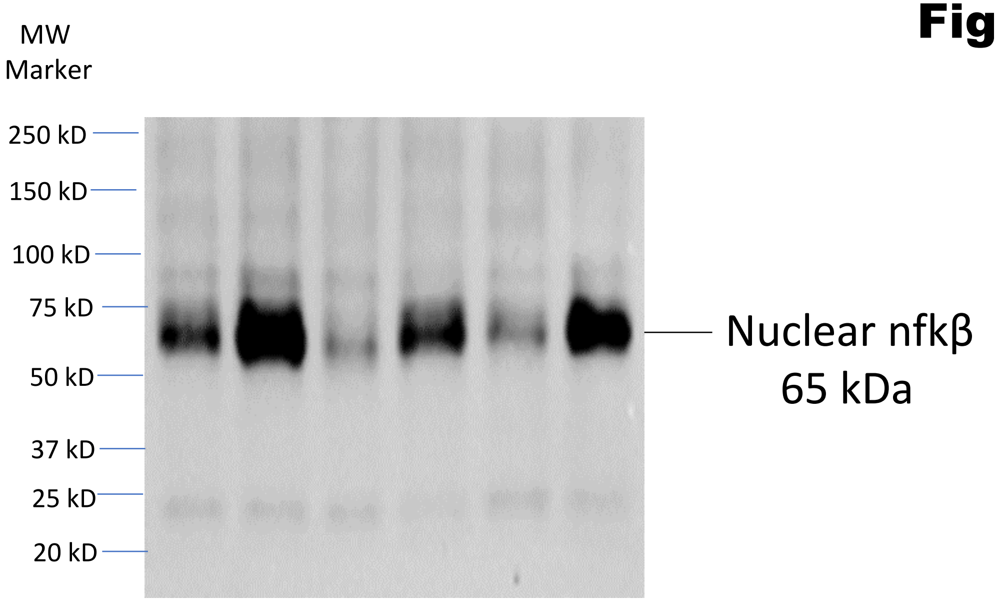
MW
Marker
250 kD
150 kD
100 kD
75 kD
50 kD
37 kD
25 kD
20 kD
Nuclear nfkβ
65 kDa

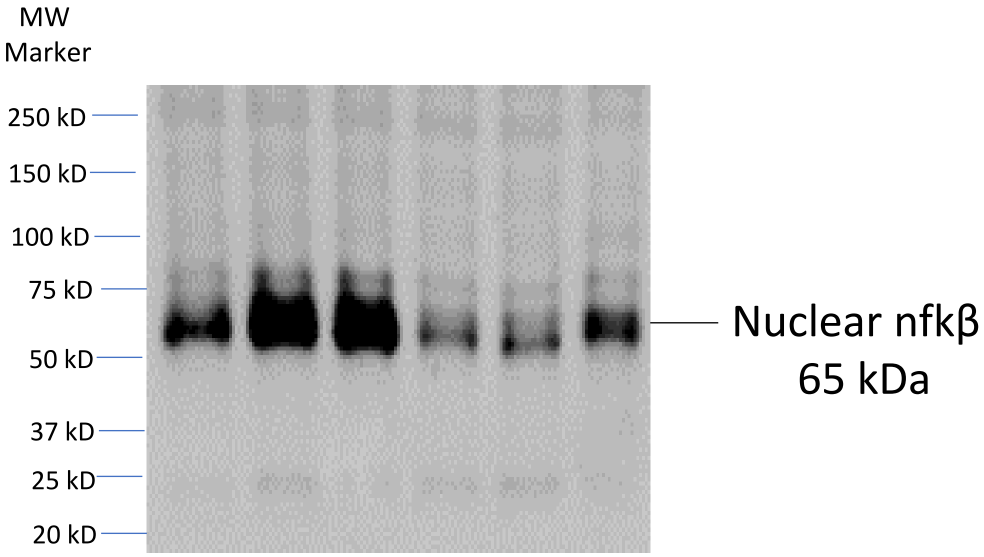
MW
Marker
250 kD
150 kD
100 kD
75 kD
50 kD
37 kD
25 kD
20 kD
Nuclear nfkβ
65 kDa

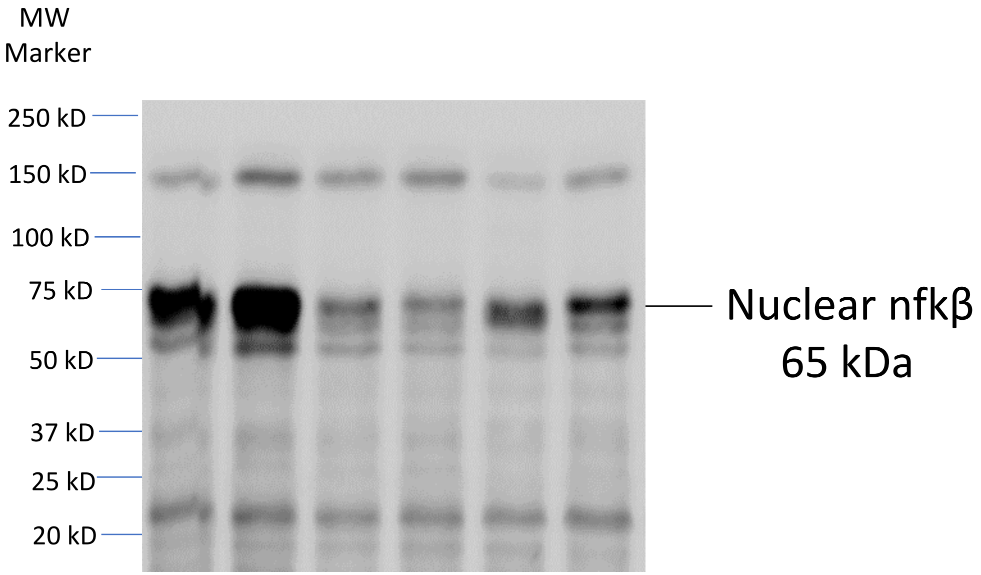
MW
Marker
250 kD
150 kD
100 kD
75 kD
50 kD
37 kD
25 kD
20 kD
Nuclear nfkβ
65 kDa

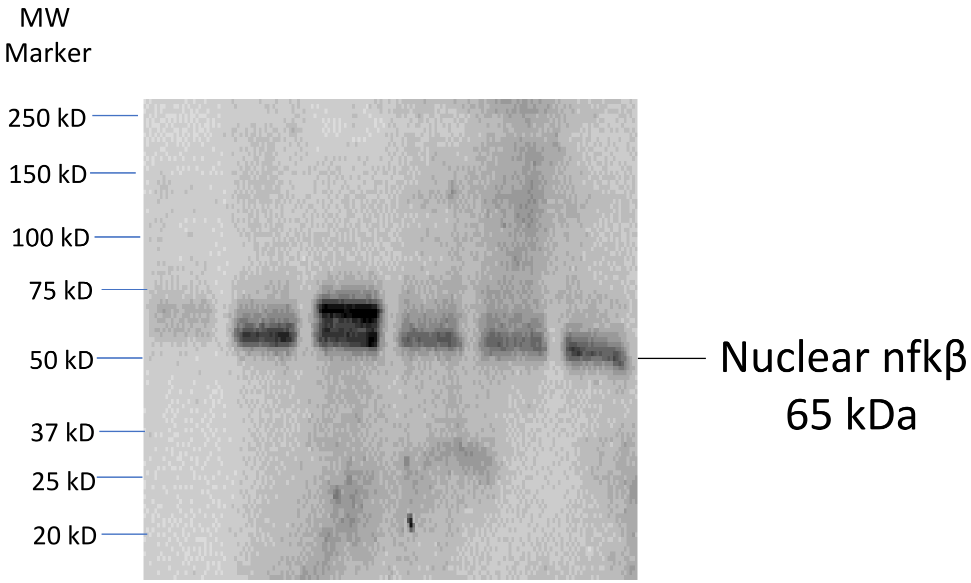
MW
Marker
250 kD
150 kD
100 kD
75 kD
50 kD
37 kD
25 kD
20 kD
Nuclear nfkβ
65 kDa

Figure S5C
Lamin B1 for Nuclear Nrf2

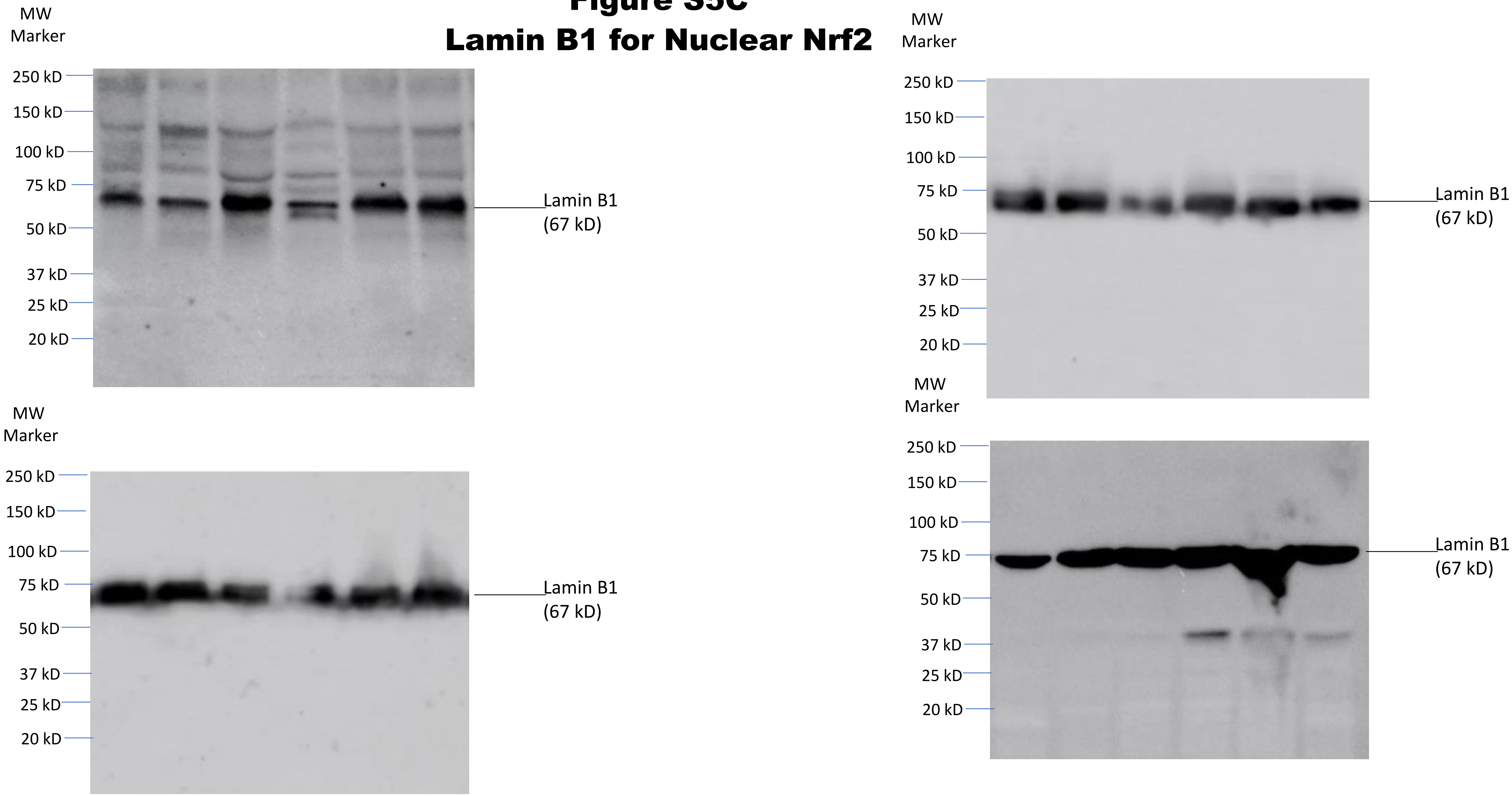

Figure S5E

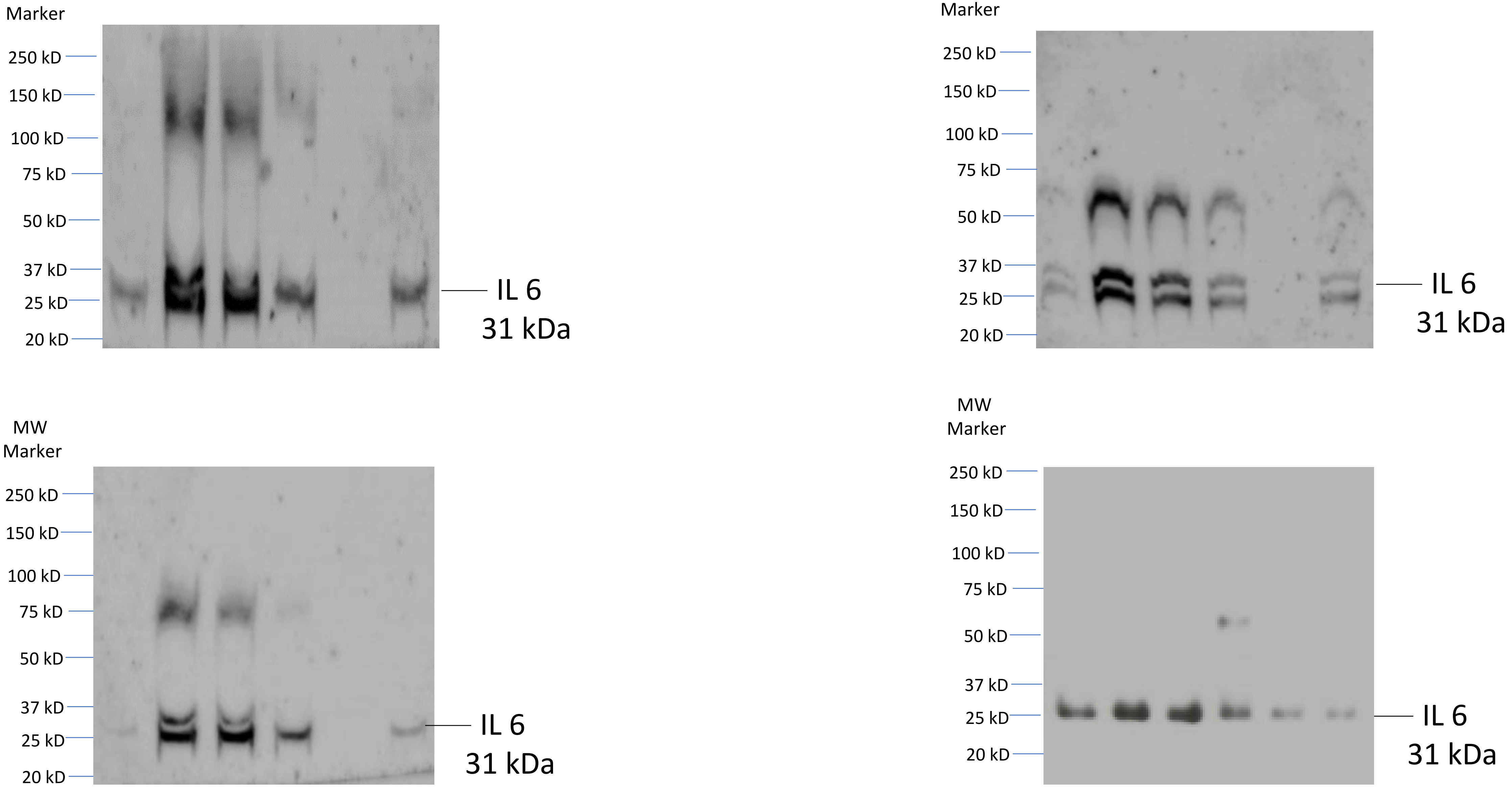
MW Marker
250 kD
150 kD
100 kD
75 kD
50 kD
37 kD
25 kD
20 kD
IL 6
31 kDa
MW Marker
250 kD
150 kD
100 kD
75 kD
50 kD
37 kD
25 kD
20 kD
IL 6
31 kDa
MW Marker
250 kD
150 kD
100 kD
75 kD
50 kD
37 kD
25 kD
20 kD
IL 6
31 kDa
MW Marker
250 kD
150 kD
100 kD
75 kD
50 kD
37 kD
25 kD
20 kD
IL 6
31 kDa

Figure S5E
β-Actin for IL 6

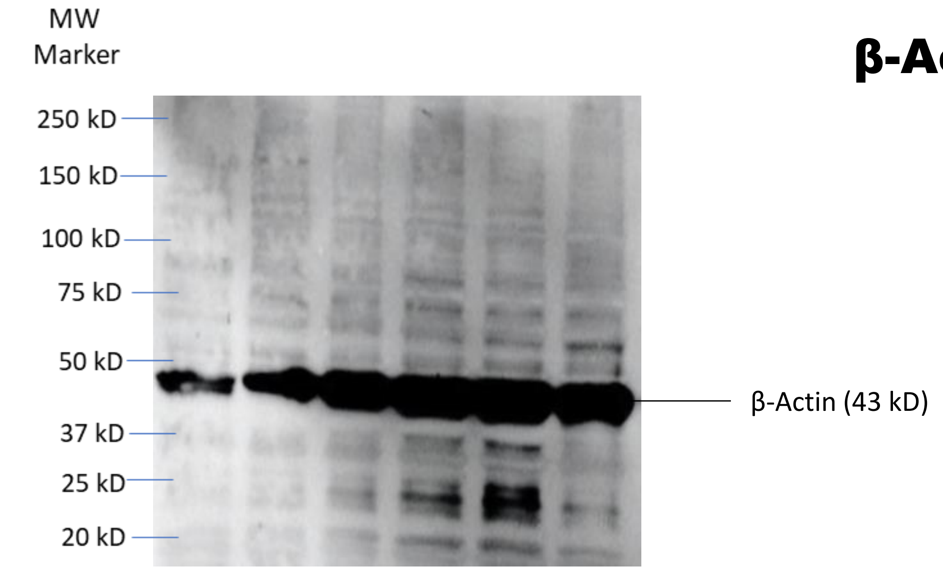

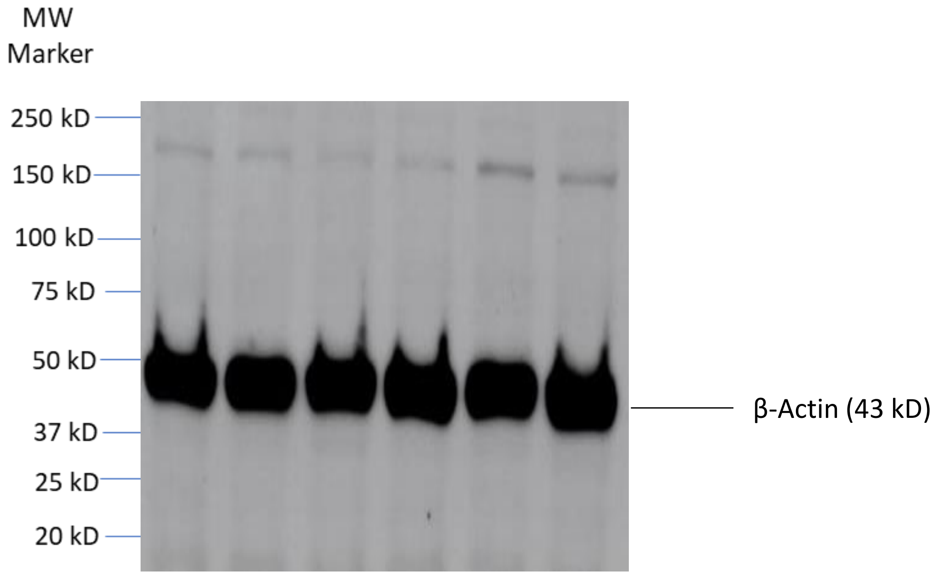

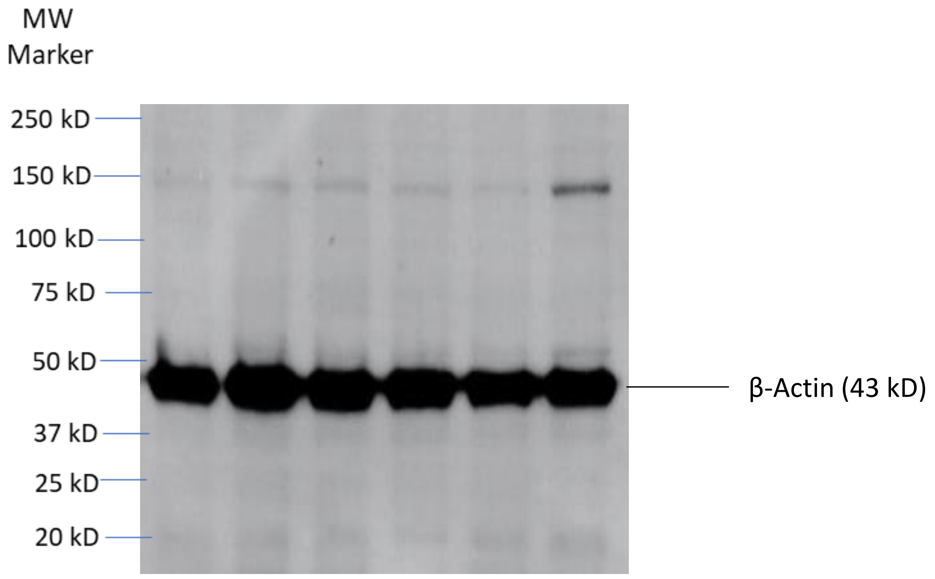

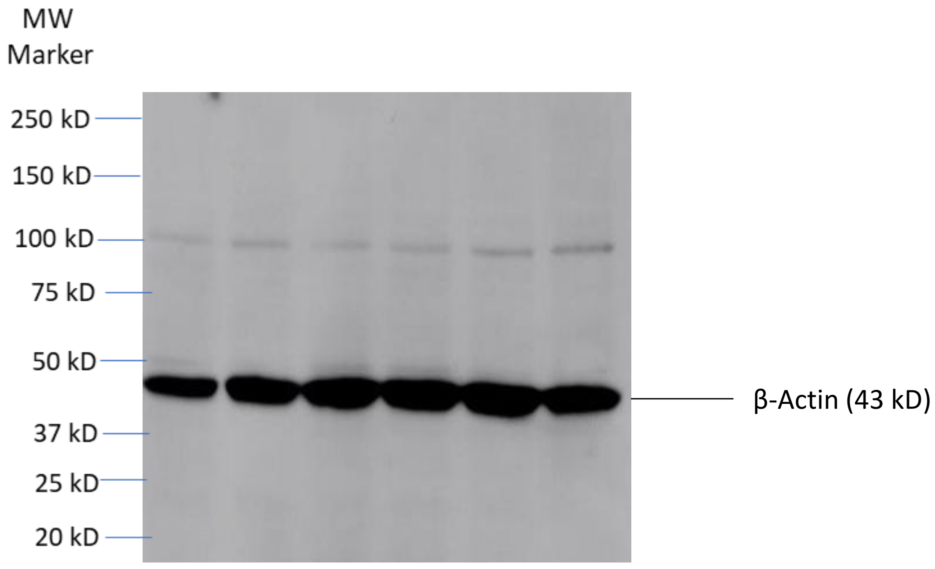

Figure S5F

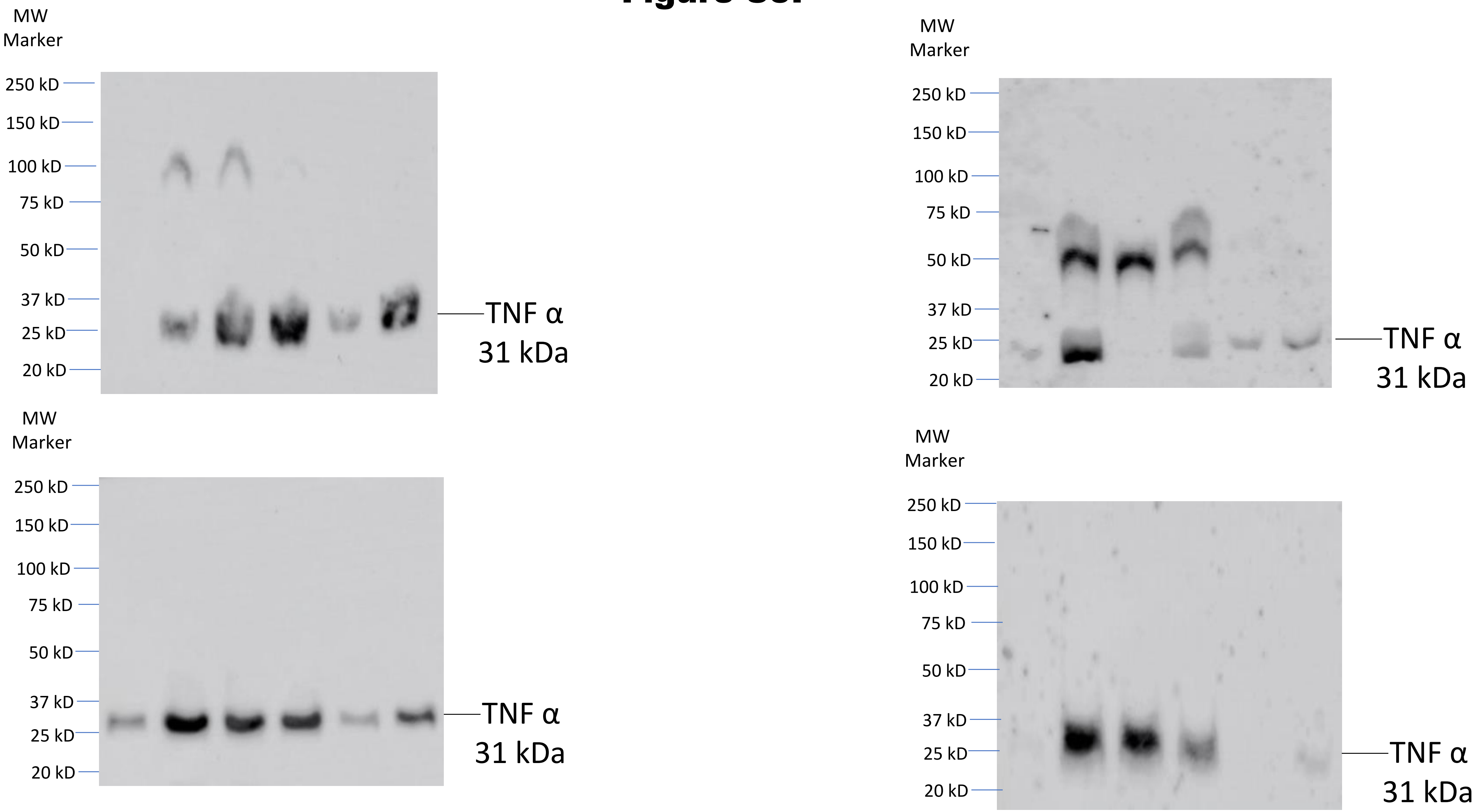
MW Marker
250 kD
150 kD
100 kD
75 kD
50 kD
37 kD
25 kD
20 kD
TNF α
31 kDa
MW Marker
250 kD
150 kD
100 kD
75 kD
50 kD
37 kD
25 kD
20 kD
TNF α
31 kDa
MW Marker
250 kD
150 kD
100 kD
75 kD
50 kD
37 kD
25 kD
20 kD
TNF α
31 kDa
MW Marker
250 kD
150 kD
100 kD
75 kD
50 kD
37 kD
25 kD
20 kD
TNF α
31 kDa

Figure S5F
β-Actin for TNF α

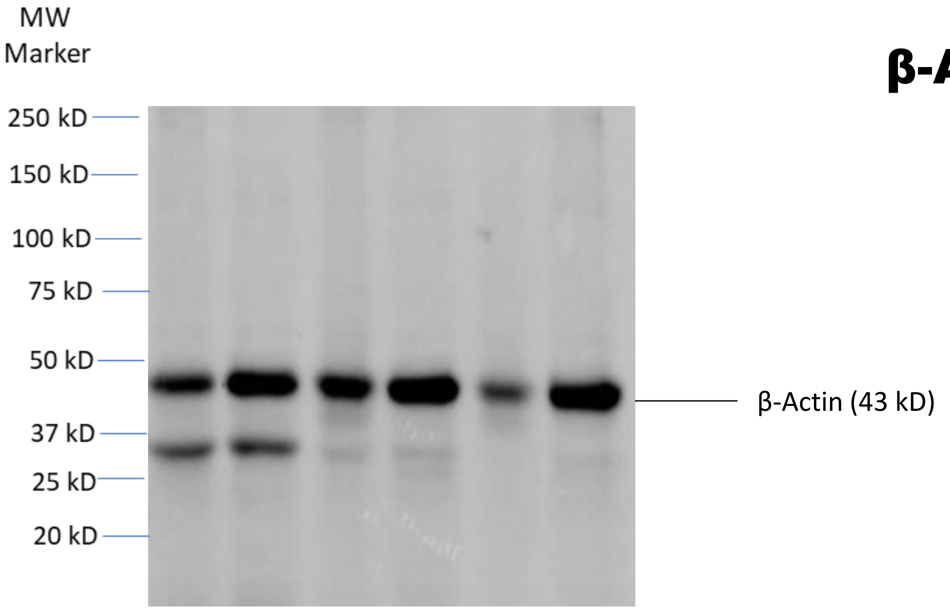

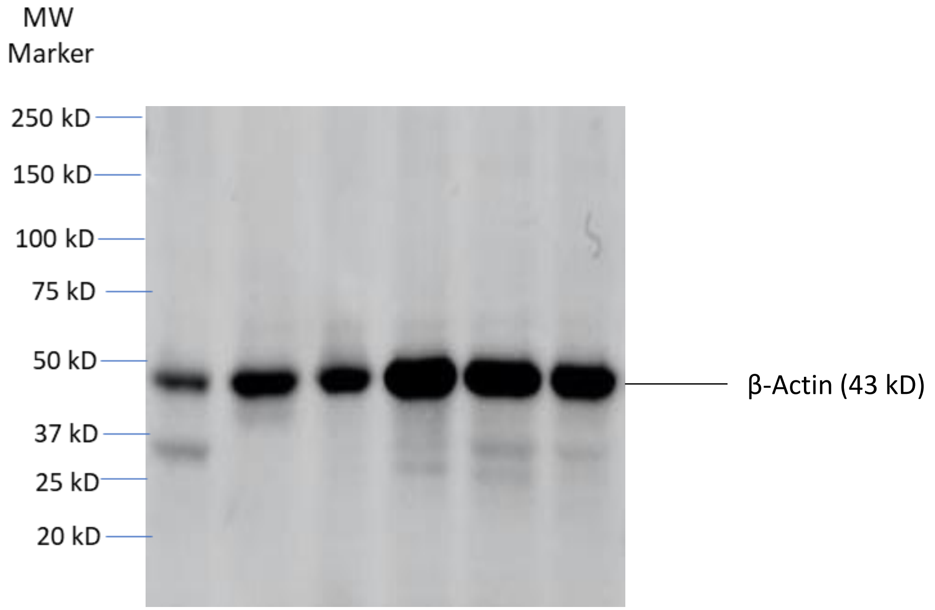

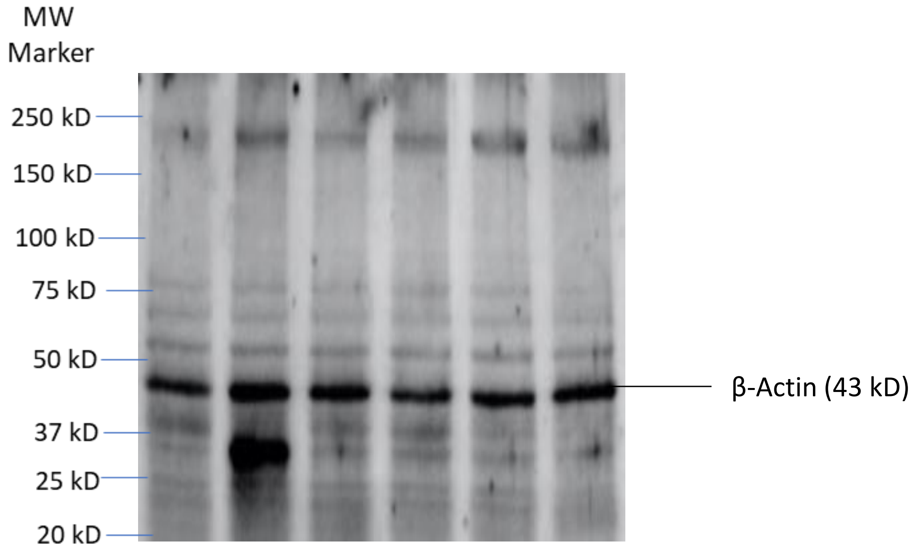

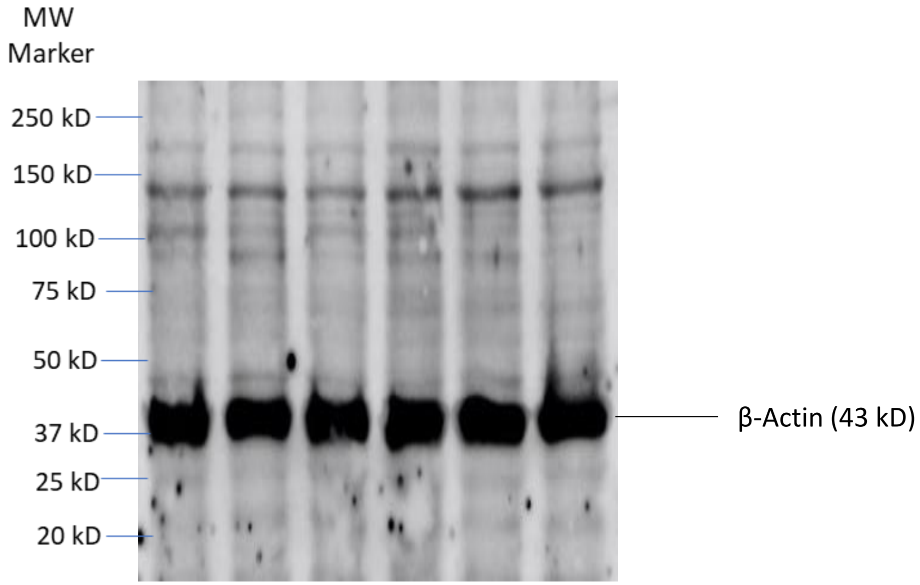

Figure S6A

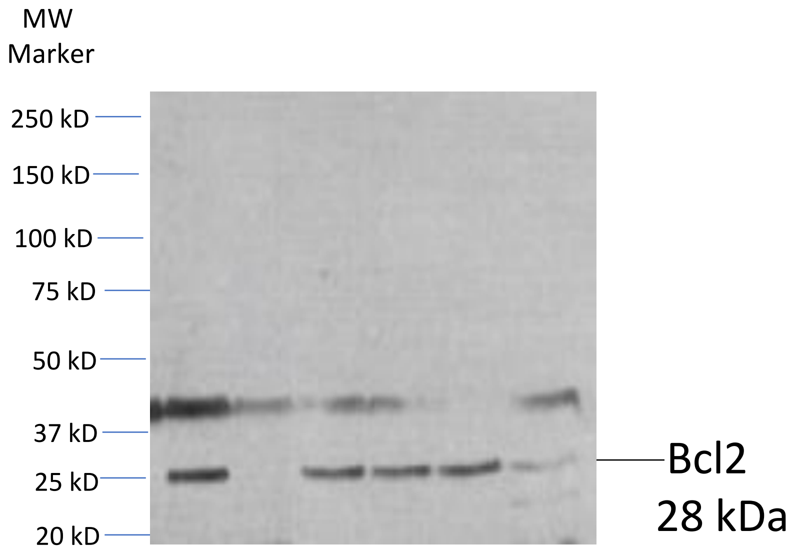
MW Marker
250 kD
150 kD
100 kD
75 kD
50 kD
37 kD
25 kD
20 kD
Bcl2
28 kDa

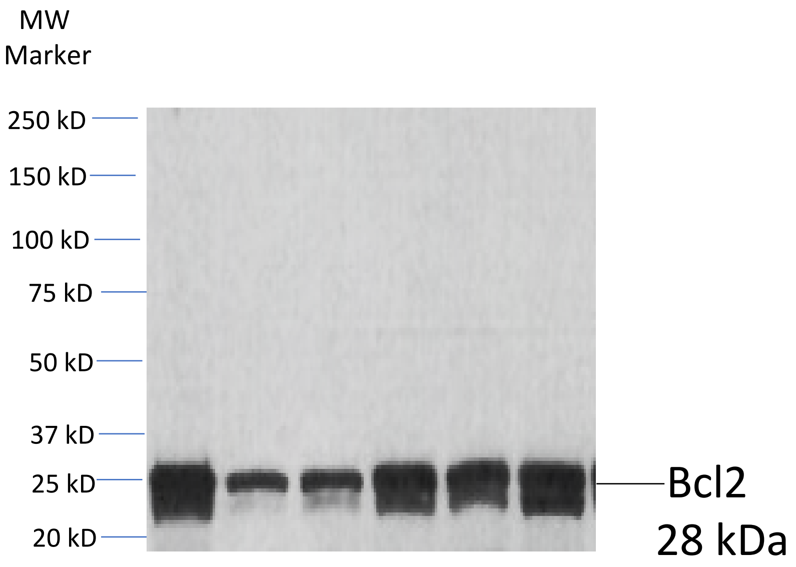
MW Marker
250 kD
150 kD
100 kD
75 kD
50 kD
37 kD
25 kD
20 kD
Bcl2
28 kDa

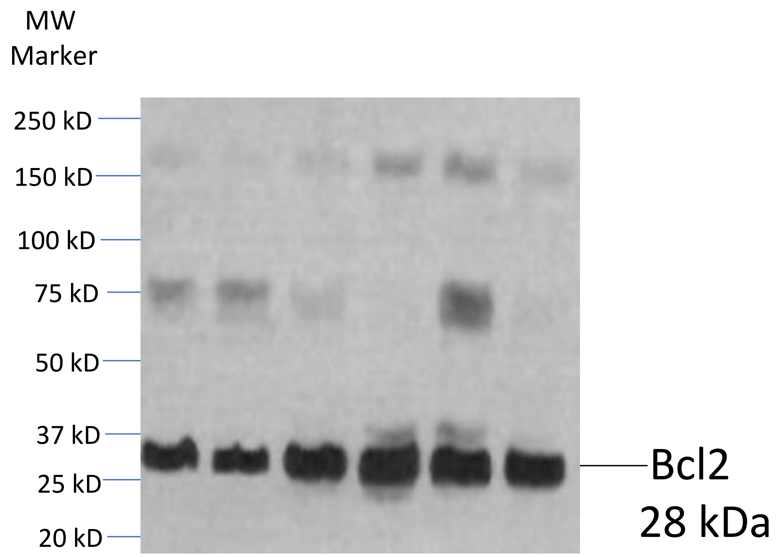
MW Marker
250 kD
150 kD
100 kD
75 kD
50 kD
37 kD
25 kD
20 kD
Bcl2
28 kDa

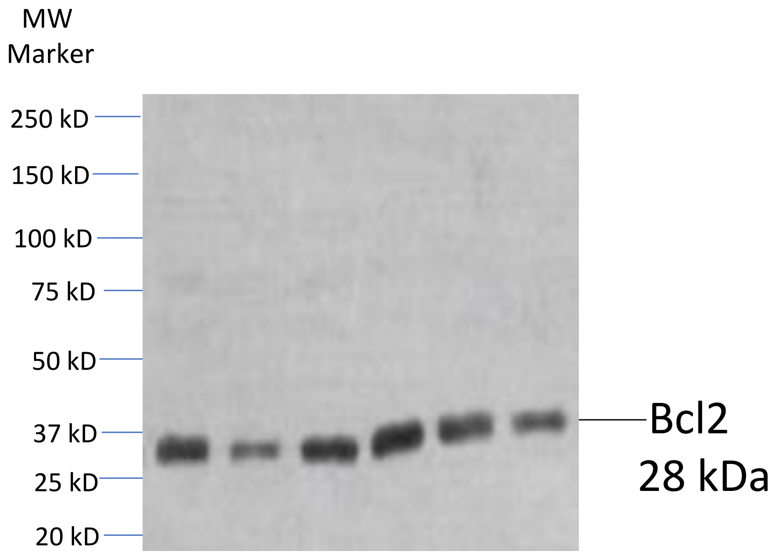
MW Marker
250 kD
150 kD
100 kD
75 kD
50 kD
37 kD
25 kD
20 kD
Bcl2
28 kDa

Figure S6A

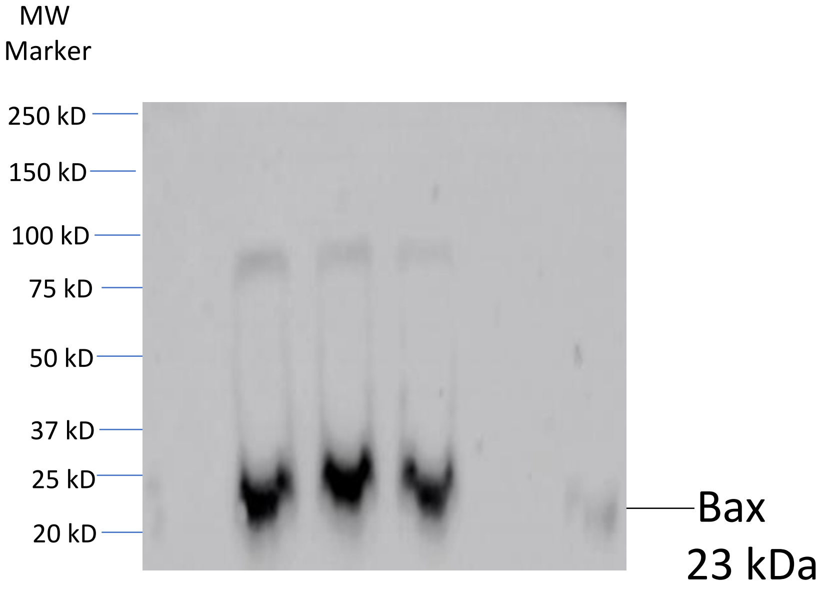
MW
Marker
250 kD
150 kD
100 kD
75 kD
50 kD
37 kD
25 kD
20 kD
Bax
23 kDa

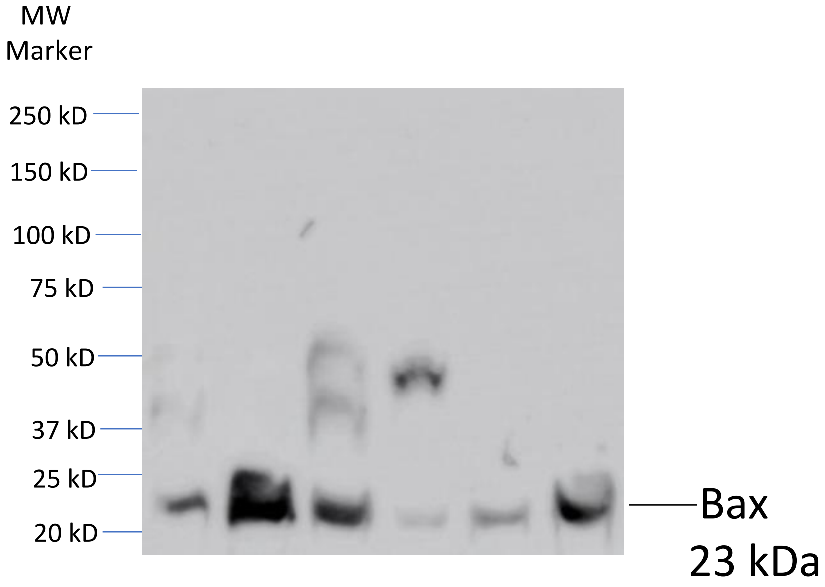
MW
Marker
250 kD
150 kD
100 kD
75 kD
50 kD
37 kD
25 kD
20 kD
Bax
23 kDa

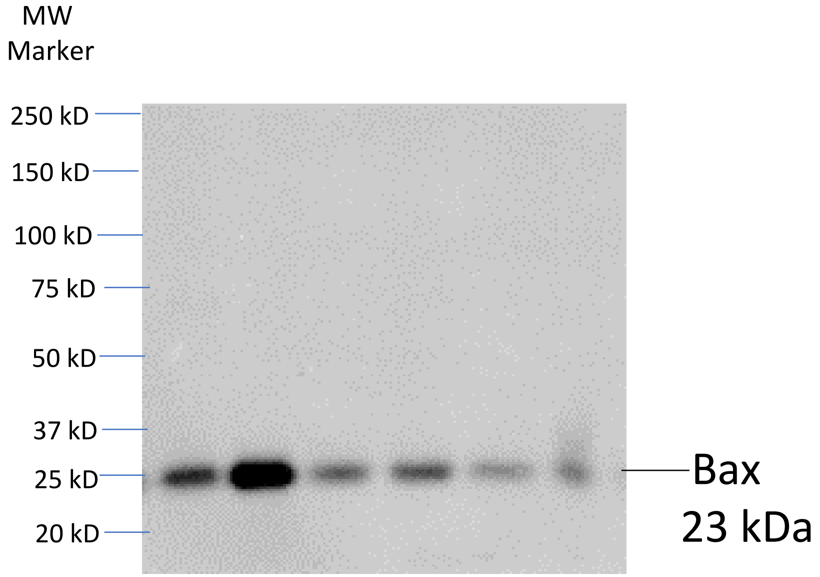
MW
Marker
250 kD
150 kD
100 kD
75 kD
50 kD
37 kD
25 kD
20 kD
Bax
23 kDa

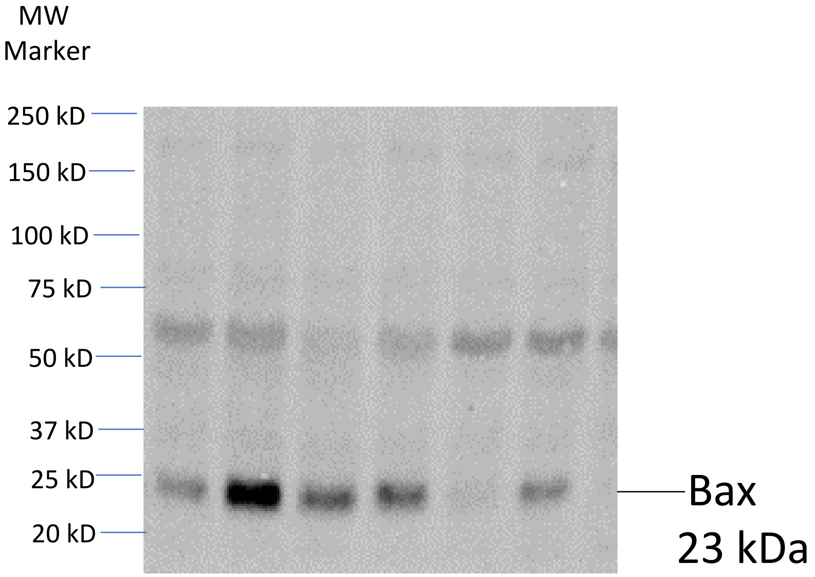
MW
Marker
250 kD
150 kD
100 kD
75 kD
50 kD
37 kD
25 kD
20 kD
Bax
23 kDa

Figure S6A

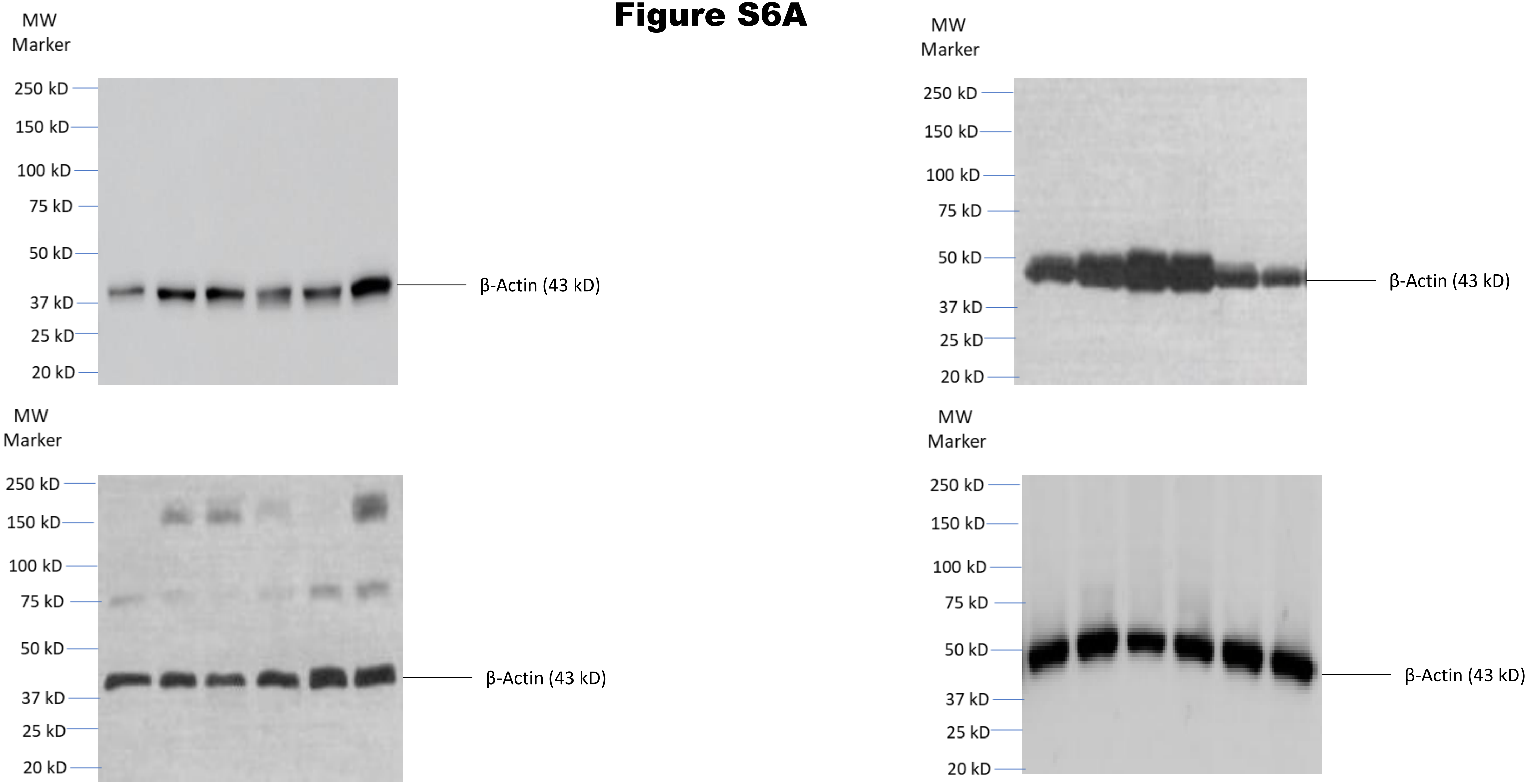
MW Marker
250 kD
150 kD
100 kD
75 kD
50 kD
37 kD
25 kD
20 kD
β-Actin (43 kD)
MW Marker
250 kD
150 kD
100 kD
75 kD
50 kD
37 kD
25 kD
20 kD
β-Actin (43 kD)
MW Marker
250 kD
150 kD
100 kD
75 kD
50 kD
37 kD
25 kD
20 kD
β-Actin (43 kD)
MW Marker
250 kD
150 kD
100 kD
75 kD
50 kD
37 kD
25 kD
20 kD
β-Actin (43 kD)

Figure S6B

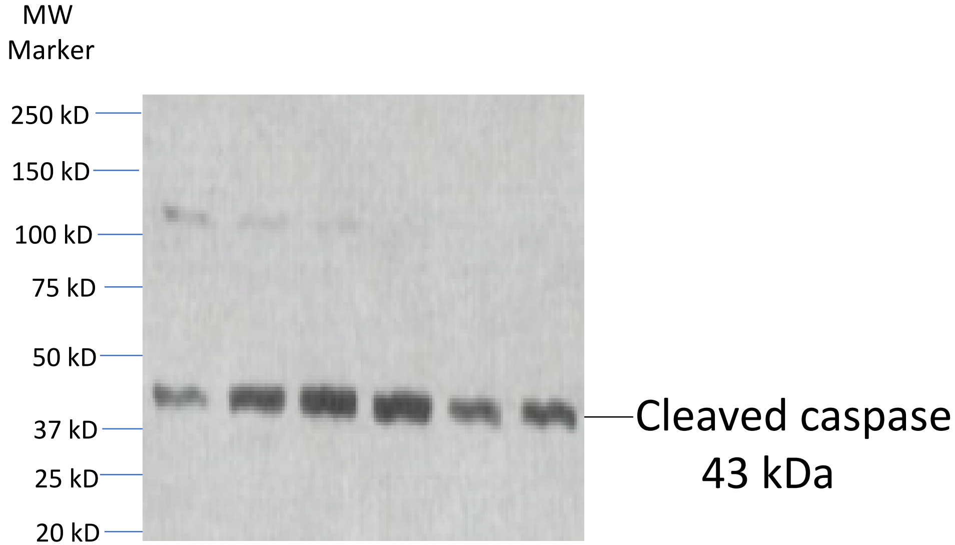
MW
Marker
250 kD
150 kD
100 kD
75 kD
50 kD
37 kD
25 kD
20 kD
Cleaved caspase
43 kDa

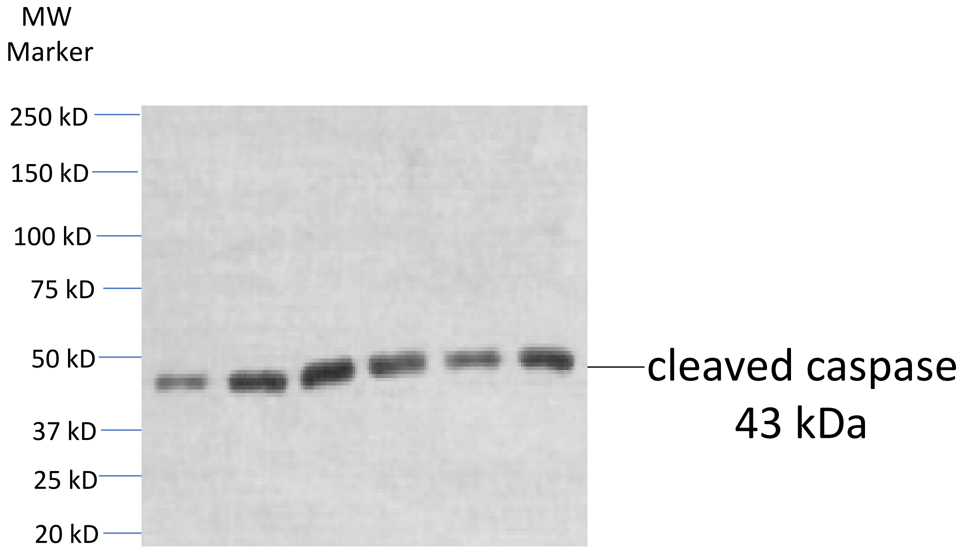
MW
Marker
250 kD
150 kD
100 kD
75 kD
50 kD
37 kD
25 kD
20 kD
cleaved caspase
43 kDa

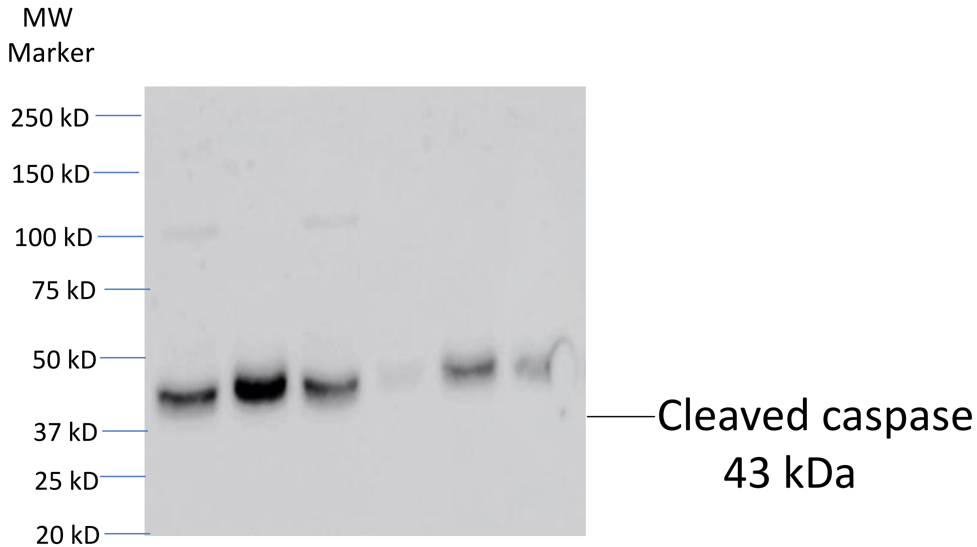
MW
Marker
250 kD
150 kD
100 kD
75 kD
50 kD
37 kD
25 kD
20 kD
Cleaved caspase
43 kDa

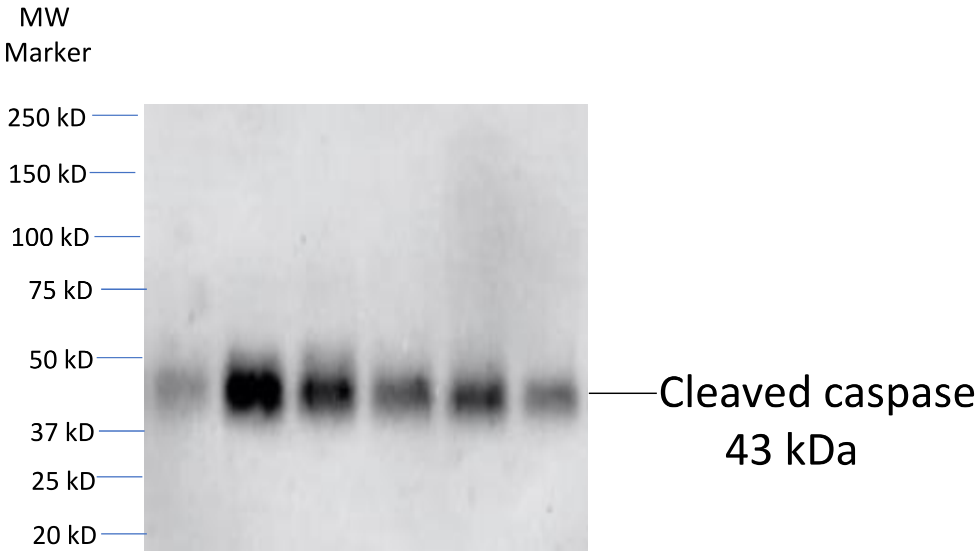
MW
Marker
250 kD
150 kD
100 kD
75 kD
50 kD
37 kD
25 kD
20 kD
Cleaved caspase
43 kDa

Figure S6B

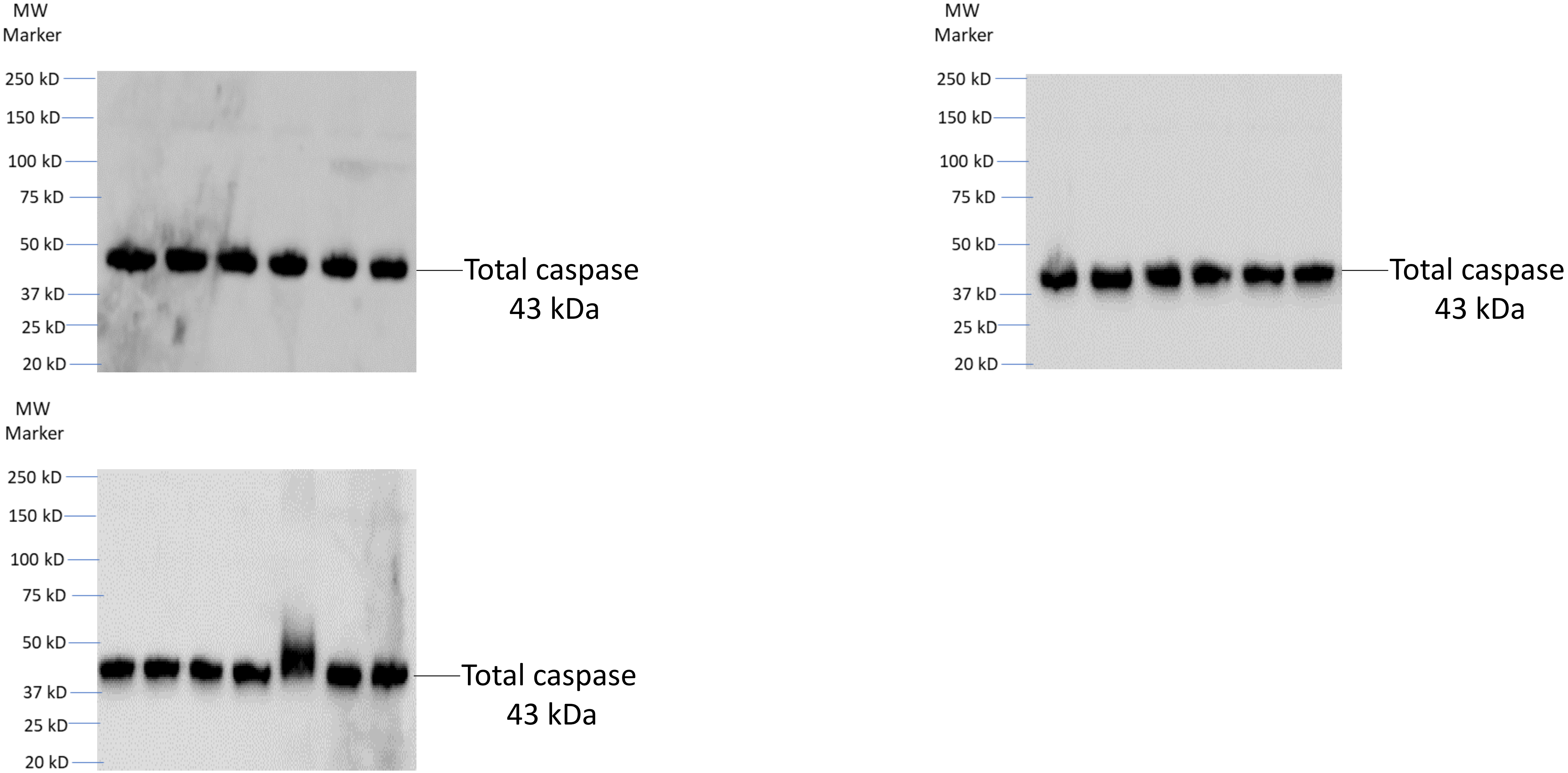
MW Marker
250 kD
150 kD
100 kD
75 kD
50 kD
37 kD
25 kD
20 kD
Total caspase 43 kDa
MW Marker
250 kD
150 kD
100 kD
75 kD
50 kD
37 kD
25 kD
20 kD
Total caspase 43 kDa
MW Marker
250 kD
150 kD
100 kD
75 kD
50 kD
37 kD
25 kD
20 kD
Total caspase 43 kDa
